# Luminescent Anionic Cyclometalated Organoplatinum (II) Complexes with Terminal and Bridging Cyanide Ligand: Structural and Photophysical Properties

**DOI:** 10.1021/acs.inorgchem.2c03668

**Published:** 2023-01-18

**Authors:** Mina Sadeghian, David Gómez de Segura, Mohsen Golbon Haghighi, Nasser Safari, Elena Lalinde, M. Teresa Moreno

**Affiliations:** † Department of Chemistry, Shahid Beheshti University, Evin, Tehran 19839-69411, Iran; ‡ Departamento de Química-Centro de Síntesis Química de La Rioja (CISQ), Universidad de La Rioja, 26006 Logroño, Spain

## Abstract

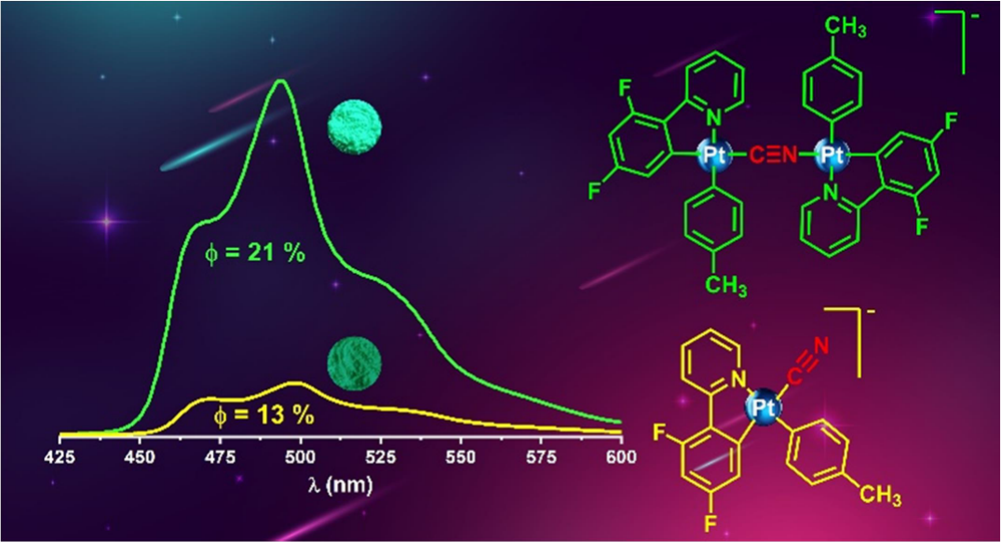

We present the synthesis and characterization of two series of mononuclear heteroleptic anionic cycloplatinated(II) complexes featuring terminal cyanide ligand Q^+^[Pt(C^N)(*p*-MeC_6_H_4_)(CN)]^−^ [C^N = benzoquinolate (bzq), Q^+^ = K^+^**1** and NBu_4_^+^**4**; 2-phenylpyridinate (ppy), Q^+^ = K^+^**2** and NBu_4_^+^**5** and 2-(2,4- difluorophenyl)pyridinate (dfppy), Q^+^ = K^+^**3** and NBu_4_^+^**6**] and a series of symmetrical binuclear complexes (NBu_4_)[Pt_2_(C^N)_2_(*p*-MeC_6_H_4_)_2_(μ-CN)] (C^N = bzq **7**, ppy **8**, dfppy **9**). Compounds **5**, **6**, and **7**–**9** were further determined by single-crystal X-ray diffraction. There are no apparent intermolecular Pt···Pt interactions owing to the presence of bulky NBu_4_^+^ counterion. Slow crystallization of K[Pt(ppy)(*p*-MeC_6_H_4_)(CN)] **2** in acetone/hexane evolves with formation of yellow crystals, which were identified by single-crystal X-ray diffraction methods as the salt complex {[Pt(ppy)(*p*-MeC_6_H_4_)(CN)]_2_K_3_(OCMe_2_)_4_(μ-OCMe_2_)_2_}[Pt(ppy)(*p*-MeC_6_H_4_)(μ-CN)Pt(ppy)(*p*-MeC_6_H_4_)]·2acetone (**10**), featuring the binuclear anionic unit **8^–^** neutralized by an hybrid inorganic–organometallic coordination polymer {[Pt(ppy)(*p*-MeC_6_H_4_)(CN)]_2_K_3_(OCMe_2_)_4_(μ-OCMe_2_)_2_}^+^. The photophysical properties of all compounds were recorded in powder, polystyrene film, and solution states with a quantum yield up to 21% for **9** in the solid state. All complexes displayed bright emission in rigid media, and for the interpretation of their absorption and emission properties, density functional theory (DFT) and time-dependent DFT calculations were applied.

## Abstract 2

This article reports heteroleptic mononuclear and binuclear cycloplatinated complexes with terminal or bridge cyanide ligands, respectively. Their synthesis, spectroscopic and X-ray characterization, optoelectronic properties, and theoretical calculations are highlighted.

## Introduction

Cyclometalated Pt(II) complexes have long been the subject of intensive investigation due to the myriad potential applications of their phosphorescence in many fields, such as organic light-emitting diodes (OLEDs),^1−5^ dye-sensitized solar cells,^6^ hydrogen production,^7^ chemical sensing,^8−12^ and bio-imaging.^13^ Their photophysical properties are strongly influenced by the spin-orbit coupling exerted by the Pt(II) core, which depend on the electronic properties of the cyclometalated and the auxiliary ligands.^14,15^ In these complexes, the strong ligand field exerted by the cyclometalated ligand, which suppresses the non-radiative decay from a dark d-d excited state, and the very fast singlet-triplet intersystem crossing facilitated by the 5d platinum lead to an efficient population of the lowest-lying triplet state and a subsequent high photoluminescent quantum efficiency.^16−18^ The result of density functional theory (DFT) and time-dependent DFT (TD-DFT) studies shows that the emission occurs mainly from ligand-centered (^3^LC) or mixed metal-to-ligand charge transfer/ligand-centered (^3^MLCT/^3^LC), or ligand-to-ligand charge transfer character depending on the auxiliary ligands.^19^ A remarkable feature of these d^8^ complexes is their ability to form excimers and aggregates in the solid state or concentrated solution through platinum–platinum contacts and/or π–π interactions. The generated self-assembled chromophores often exhibit new low-energy (LE) emissions, which are assigned to come from metal–metal to ligand charge transfer excited states arising from intermolecular coupling between *d*_z2_ orbitals protruding out of the platinum coordination planes.^19−24^ In the monomers, excited state tuning has proven to be quite feasible through modification of the cyclometalating ring systems, either by the addition of substituents expanding the size of the π system or by introducing heteroatoms. The effect of the auxiliary ligands has also been widely explored. These can be varied from monodentate to bidentate including a wide range of electron withdrawing/donating properties. In this context, coordination of strong field auxiliary ligands are desirable. The effect of the coordination by pentafluorophenyl,^25−27^ alkynyl (C≡CR),^28,29^ and isocyanide (C≡NR)^24,30−35^ as auxiliary ligands in heteroleptic cyclometalated [Pt(C^N)L_2_]^*n*^ (*n* = −1, 0, and +1) complexes have been explored by us and others, and some of these complexes have been proven to be excellent building blocks for the preparation of clusters,^26,36,37^ and extended aggregates^38,39^ with intriguing optical properties.^40−42^ Cyanide is another important strong field ligand with very strong σ-donor ability, which has been widely exploited not only in the Pt(II) chemistry but also with other d^6^ metal ions to form complexes that emit efficient blue phosphorescence.^43−47^ Furthermore, in accordance with the well-stablished capability of the cyanide ligand to act as a compact bridging ligand, some of these complexes have been successfully exploited to construct heteropolynuclear complexes in triangular, square, and hexagonal forms featuring bridging ligands^48^ or additionally stabilized by metallophillic interactions,^39^ which have attractive optical properties. In recent years, dicyanide cycloplatinated anionic units have been employed to form soft salts [Pt(C^N)(en)][Pt(C^N)(CN)_2_] (en = ethylenediamine) exhibiting interesting reversible excitation-wavelength-dependent behavior owing to the modulation of the Pt(II)···Pt(II) bond interaction by means of mechanical and vapor fuming.^49^ Recently, we have reviewed the literature on group 10 transition metal complexes featuring the cyanide ligand to highlight their structural and physical, chemical, and optical properties with potential applications in many fields.^50^ Among these complexes, simple alkali salts of dicyanide cycloplatinated compounds stand out due to their vapochromic properties. Sicilia and co-workers^51,52^ reported the formation of coordination polymers [K(H_2_O)][Pt(ppy)(CN)_2_] and [K(H_2_O)][M(bzq)(CN)_2_] (M = Pt, Pd), in which the K^+^ are bonded to cyanide and H_2_O molecules, which display vapochromic behavior upon drying and exposure to H_2_O. Recently, Shigeta et al. revealed that the potassium ions in K[Pt(Cl_2_ppy)(CN)_2_] act as vapor coordination sites toward *N*,*N*-dimethylacetamide and *N*,*N*-dimethylformamide vapors with structural and luminescence changes.^53^

As part of our interest was designing new photoluminescent complexes bearing cyanide ligands, in the present study, we present the synthesis and characterization of a series of anionic heteroleptic mononuclear complexes [Pt(C^N)(*p*-MeC_6_H_4_)(CN)]^−^ [C^N = benzoquinolate (bzq), phenylpyridinate (ppy), 2-(2,4-difluorophenyl)pyridinate) (dfppy)] with two different kinds of counterions, namely, potassium (**1**–**3**) and tetrabutylammonium (**4**–**6**). Slow crystallization of K[Pt(ppy)(*p*-MeC_6_H_4_)(CN)] **2** in acetone/hexane afforded yellow crystals, which were identified by single-crystal X-ray diffraction methods as the salt complex {[Pt(ppy)(*p*-MeC_6_H_4_)(CN)]_2_K_3_(OCMe_2_)_4_(μ-OCMe_2_)_2_}[Pt(ppy)(*p*-MeC_6_H_4_)(μ-CN)Pt(ppy)(*p*-MeC_6_H_4_)]·2acetone (**10**), featuring the new binuclear anion [Pt(ppy)(*p*-MeC_6_H_4_)(μ-CN)Pt(ppy)(*p*-MeC_6_H_4_)]^−^. Notably, reports on simple mono-bridged CN binuclear platinum complexes are rare,^54,55^ despite the wide range of ordered assemblies generated by cyanide bridges. Also, binuclear complexes (NBu_4_)[Pt(C^N)(*p*-MeC_6_H_4_)(μ-CN)Pt(C^N)(*p*-MeC_6_H_4_)] (**7**–**9**) were easily prepared and characterized by X-ray diffraction studies. The photophysical properties of these compounds are described in detail, in solution, solid state, and in polymer films, and DFT and TD-DFT calculations are applied to support the absorption and emission spectra of these complexes.

## Results and Discussion

### Synthesis and Characterization

**Scheme 1:**
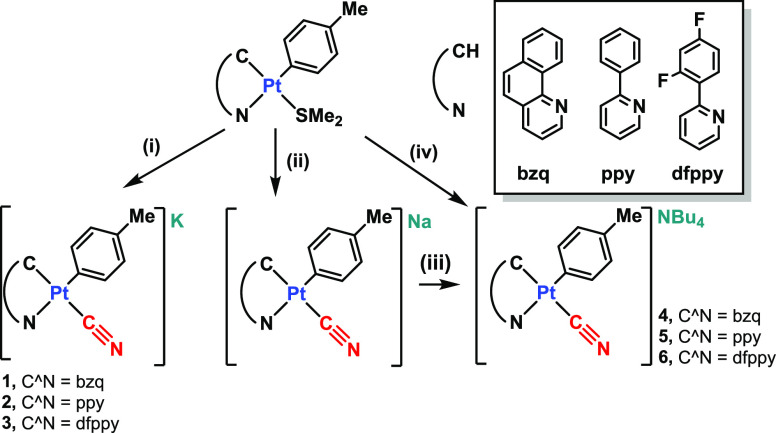
Synthesis of **1**–**6**, (i) KCN (0.97 equiv), MeOH/H_2_O, 298 K; (ii) NaCN (1.05 equiv), DMSO, 298 K; (iii) (NBu_4_)ClO_4_ (1 equiv), 298 K; and (iv) (NBu_4_)CN (1 equiv), Acetone, 298 K

The synthetic routes for the preparation of novel cyanide complexes Q^+^[Pt(C^N)(*p*-MeC_6_H_4_)(CN)]^−^ [C^N = bzq, Q^+^ = K^+^**1**, NBu_4_^+^**4**; ppy, Q^+^ = K^+^**2**, NBu_4_^+^**5**; dfppy, Q^+^ = K^+^**3**, NBu_4_^+^**6**] are summarized in Scheme 1. The precursors [Pt(C^N)(*p*-MeC_6_H_4_)(SMe_2_)] (C^N = bzq, ppy, dfppy) were prepared^56,57^ following reported procedures by refluxing of *cis*-[Pt(*p*-MeC_6_H_4_)_2_(SMe_2_)_2_] with the corresponding C^NH ligands in acetone for 12 h. In a second step, the displacement of the SMe_2_ ligand by cyanide, using KCN in a mixture MeOH/H_2_O, in the case of complexes **1**–**3** (Scheme 1i) or (NBu_4_)CN in acetone, for complexes **4**–**6** (Scheme 1iv), allows for the synthesis of the final compounds as yellow solids, in moderate to good yields. Complexes **4**–**6** can be also alternatively prepared by treatment of the in situ prepared [Pt(C^N)(*p*-MeC_6_H_4_)(CN)]^−^ complexes, by using NaCN (1.05 equiv) in dimethyl sulfoxide, with (NBu_4_)ClO_4_ (Scheme 1ii,iii) (see the Experimental Section for details). Complexes **1**–**3** were completely dried in an oven at 110 ° C to eliminate traces of retained water. In this process, no changes in color or emissions were observed, contrasting with previous reported behavior in dicyanoplatinates.^51−53^

The identity of all mononuclear complexes has been established in the solid state by elemental analysis, IR, and single-crystal X-ray for **5** and **6** and, in solution, using ESI-Mass spectra and ^1^H,^13^C{^1^H}, ^19^F{^1^H} (**3**, **6**) and ^195^Pt NMR spectroscopy. The signals were assigned on the basis of ^1^H-^1^H(COSY) and ^13^C-^1^H correlations (HSQC and HMBC). The spectra are included in Figures S1–S12. All complexes showed in their IR spectra one characteristic ν(C≡N) absorption band at 2087–2103 cm^–1^, confirming the terminal carbon-bonded cyanide ligand, and their ESI mass spectra, in the negative mode, exhibit the corresponding molecular peak [Pt(C^N)(p-C_6_H_4_Me)(CN)]^−^ (100% **1**, **3**, **4** and **6**) regardless of the cation (Figure S13). The ^1^H and ^13^C{^1^H} NMR spectra in MeOD show the expected signals for the cyclometalated, aryl, and NBu_4_^+^ groups (see the Experimental section). Coordination of the cyanide ligand is reflected in distinctive differences in the cyclometalated ring in relation to the corresponding precursors.^56,57^ Thus, the proton H^2^, adjacent to the N atom, is notably shifted to high frequencies (δ 9.31–9.58 vs 8.87–9.13 in precursors in MeOD), a fact attributable to the better electron-withdrawing capability of the CN^−^ in relation to the SMe_2_. In the ^13^C{^1^H} NMR spectra, the resonance of the cyanide was found in the range 154.9–157.4 ppm, although no platinum satellites were resolved. To further confirm and accurately assign the resonance of the cyanide, the ^13^C{^1^H} NMR spectrum of the in situ prepared mononuclear complex K[Pt(ppy)(*p*-MeC_6_H_4_)(^13^CN)] in MeOD, **2′**, was also recorded. **2′** displays a sharp resonance at δ 157.4 flanked with ^195^Pt satellites (^1^*J*_Pt–C_ = 882 Hz) (Figure S3). This signal and, particularly, the coupling constant compares well with the values reported for the resonance of the CN^−^ ligand *trans* to C_bzq_ in the compound (NBu_4_)[Pt(bzq)(^13^CN)_2_],^58^ (δ 144.2, ^1^*J*_Pt–C_ = 832 Hz). This is in accordance with the position of CN^−^ ligand *trans* to the metallated carbon, further supporting the position of the cyanide in their crystal structures. The ^195^Pt NMR spectra of complexes **1**–**6** exhibit one signal (δ −3706 to −3764) in the typical spectral range for related cyclometalated Pt(II) complexes.^33, 57, 59^ In the potassium complex **2**, upon long acquisition time, two small new signals, due to the formation of the corresponding binuclear species **8**, were observed.

**Figure 1:**
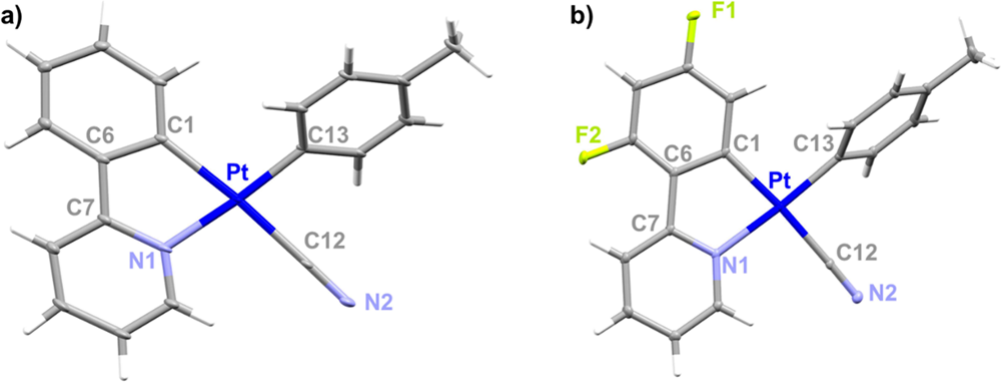
Molecular structures of the mononuclear platinum(II) anions for (a) **5** and (b) **6**.

**Figure 2:**
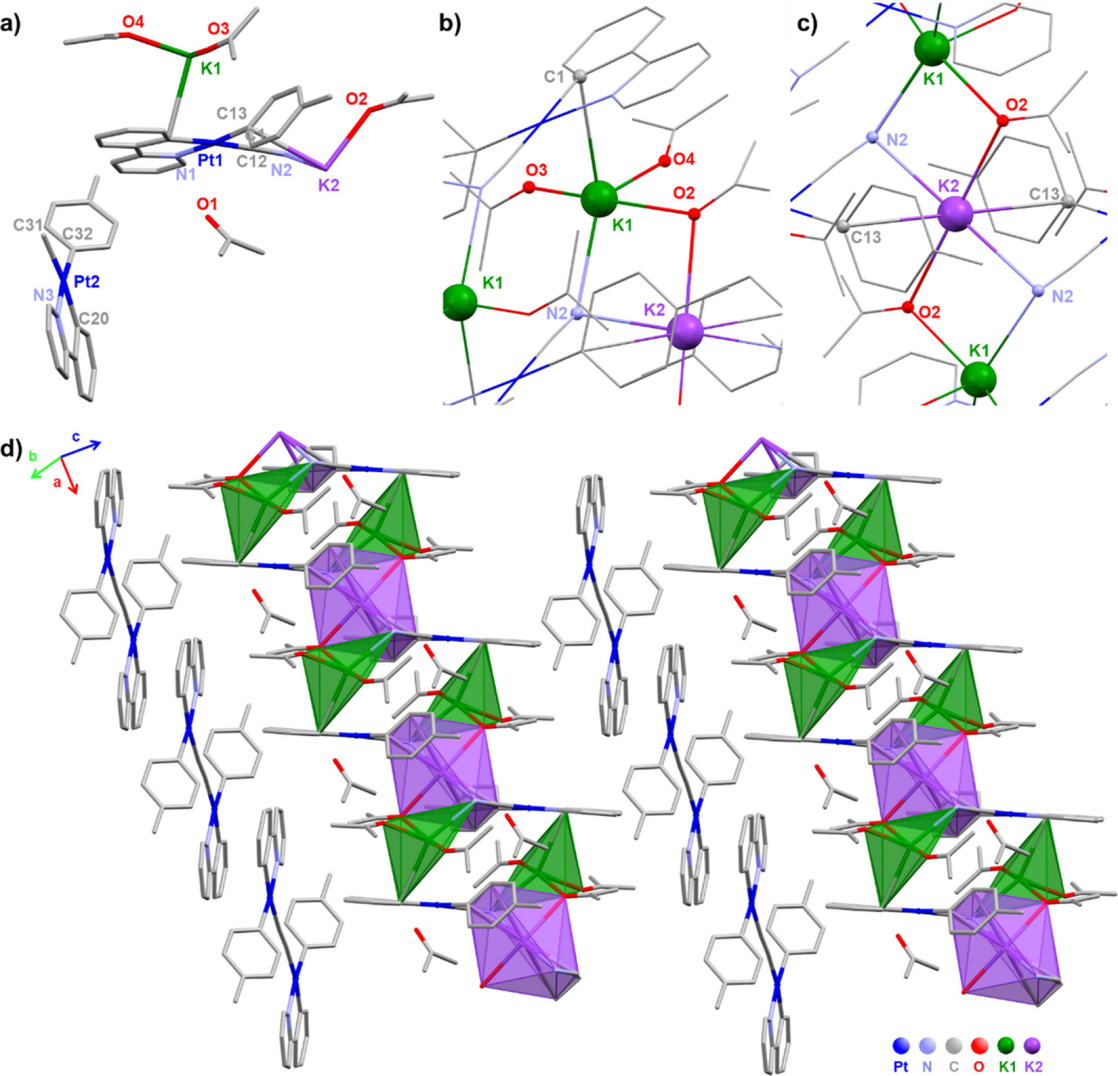
(a) Asymmetric unit of the molecular structure hybrid inorganic–organometallic polymer salt of complex **10**. Environments of (b) K (1) and (c) K (2). (d) Extended molecular packing.

For complexes **5** and **6**, yellow crystals suitable for X-ray diffraction studies were obtained by slow diffusion of *n-*hexane into a solution of the compounds in acetone. Structure refinement data and selected bond lengths and angles are given in Tables S1 and 1, respectively. The structures of the anions (Figure 1) confirm that their formation has taken place with retention of the stoichiometry of the precursor. Thus, the CN^−^ is in *trans* position to the carbon of the cyclometalated ligand. The Pt(II) center is located in distorted square-planar environments formed by the donor atoms of a cyclometalated group, the cyanide, and the *p*-MeC_6_H_4_ ligand, which is located tilted to the platinum plane (angles 47.32° **5** and 58.47° **6**). As predicted, the Pt–C_C^N_ bond length is significantly longer in complexes **5** and **6** than in the precursor complexes, [Pt(C^N)(*p*-MeC_6_H_4_)(SMe_2_)],^57,60^ which is in agreement with the stronger *trans* influence of cyanide ligand compared to the SMe_2_. In its turn, the Pt–C_cyanide_ [2.149 (2) **5**; 2.013 (3) Å **6**] are slightly longer than the Pt–C_tol_ distances [1.872 (2) **5**; 2.010 (3) Å **6**], reflecting the high *trans* influence of the metalated carbon, and are in line with earlier reports on cycloplatinated cyanometallates.^61^ The Pt-N distances are unexceptional and in the range expected for this type of bonds. The crystal packing only shows weak C–H···π interactions (2.804–2.952 Å) between the anions and the bulky tetrabutylammonium cations (Figure S14). Unfortunately, all attempts to obtain suitable crystals for the potassium salts were unsuccessful. Surprisingly, by slow diffusion of *n-*hexane into an acetone solution of **2** at low temperature (−30 °C), a small amount of yellow crystals were grown, which were identified by single-crystal X-ray diffraction methods as the unusual salt complex {[Pt(ppy)(*p*-MeC_6_H_4_)(CN)]_2_K_3_(OCMe_2_)_4_(μ-OCMe_2_)_2_}[Pt(ppy)(*p*-MeC_6_H_4_)(μ-CN)Pt(ppy)(*p*-MeC_6_H_4_)]·2acetone (**10**), featuring the new binuclear anion [Pt(ppy)(*p*-MeC_6_H_4_)(μ-CN)Pt(ppy)(*p*-MeC_6_H_4_)]^−^ (Figure 2 and Table 2). The cationic part is an hybrid inorganic–organometallic polymer generated by interaction of the expected anion **2^–^** with solvated potassium ions with different environments, {[Pt(ppy)(*p*-MeC_6_H_4_)(CN)]_2_K_3_(OCMe_2_)_4_(μ-OCMe_2_)_2_}^+^. Two of the K^+^ ions [K(1), drawn in green] exhibit a pseudo-trigonal bipyramid environment being bonded to the N(2) of the cyanide [acting as bridge, μ-N(2)–K(1):K(2)], to three oxygen atoms of acetones [two terminal, O(3,4) and one bridging, μ-O(2)–K(1):K(2)] and to the π electron density of the neighboring ppy ligand [K(1)–C(1) 3.149(4) Å]. The third potassium ion [K(2), drawn in purple] displays a pseudo-octahedral environment contacting weakly with two O(2) atoms of bridging acetones [μ-O(2)–K(1):K(2)], two N(2) of bridging cyanides [μ-N(2)–K(1):K(2)], and with the π electron density of two adjacent tolyl groups [K(2)–C(13) 3.263(4) Å]. The observed terminal and bridging K···O distances [K···O_t_ 2.642(5)–2.654 Å; K···O_b_ 2.773(4)–2.790(3) Å], the K···N_cyanide_ lengths [2.770(4)–2.954(4) Å], and K···N–C angles [86.9(2)–127.9(3) °] are comparable to those reported by Sicilia and co-workers^51^ in complex [K(OCMe_2_)_2_][Pt(ppy)(CN)_2_] and those reported in K[Pt(Cl_2_ppy)(CN)_2_]·3DMA (DMA = *N,N′-*dimethylacetamide).^53^ The ^1^H NMR spectrum of these crystals (**10**) in (CD_3_)_2_CO is rather complex but confirms the presence of three distinct ppy ligands visible in the most deshielded H^2^: one doublet signal at δ 9.48 ppm, close to that seen in **2**, which is ascribed to the monomer unit, and two doublet signals at δ 9.02 and 8.93 ppm for the unsymmetrical bimetallic anionic unit **8^–^** (Figure S12).

**Table 1:** Selected Distances (Å) and Angles (°) for Complexes **5** and **6**

parameter	5	6
Pt(1)–C_C^N_	2.179(2)	2.030(3)
Pt(1)–N(1)	1.9776(18)	2.094(2)
Pt(1)–C_cyanide_	2.149(2)	2.013(3)
Pt(1)–C_tol_	1.872(2)	2.010(3)
C≡N	1.236(3)	1.155(3)
C(1)–Pt(1)–N(1)	71.76(8)	80.08(10)

**Table 2:** Selected Distances (Å) and Angles (°) for **10**

Pt(1)–N(1)	2.101(3)	K(1)–O(4)	2.654
Pt(1)–C(1)	2.039(4)	K(1)–N(2)	2.770(4)
Pt(1)–C(12)	2.013(4)	K(1)–C(1)	3.149(4)
Pt(1)–C(13)	2.009(4)	K(2)–O(2)	2.790(3)
Pt(2)–N(3)	2.106(3)	K(2)–N(2)	2.954(4)
Pt(2)–C(20)	2.008(4)	K(2)–C(13)	3.263(4)
Pt(2)–C(31)	2.034(4)	K(1)–N(2)–C(12)	127.9(3)
Pt(2)–C(32)	2.001(4)	K(2)–N(2)–C(12)	89.77
K(1)–O(2)	2.774(3)	K(1)–O(2)–K(2)	94.38(9)
K(1)–O(3)	2.642(5)	K(1)–N(2)–K(2)	90.91

**Scheme 2:**
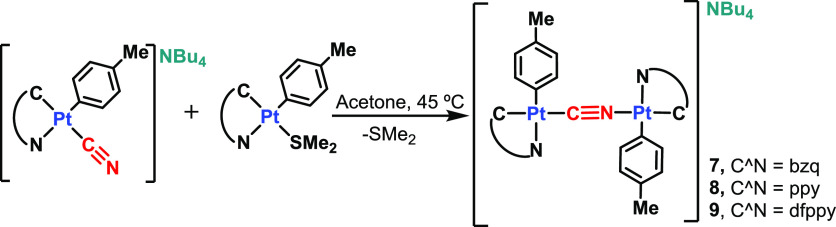
Synthesis of **7**–**9**

Notably, diplatinum complexes in which the metal centers are only connected by a cyanide ligand are rather rare. As far as we know, there are only two old reports on simple mono-bridged CN^−^ binuclear platinum complexes,^54,55^ and nothing is known about their optical properties. Therefore, we considered it of interest to explore its formation. First, it was observed that compounds **1**–**3** were stable in the solid state and in MeOD, but in acetone solvent evolve slowly giving rise, in the case of complex **2**, to a similar spectrum to that obtained for the yellow crystals of **10**. Therefore, we sought first to examine the reactivity of the monometallic (NBu_4_)[Pt(C^N)(*p*-MeC_6_H_4_)(CN)] derivatives **4**–**6** toward neutral substrates [Pt(C^N)(*p*-MeC_6_H_4_)(SMe_2_)], aiming to see if the terminal CN^−^ ligand in the anionic precursors is able to displace the SMe_2_ ligand in the neutral ones. As outlined in Scheme 2, discrete bimetallic anionic complexes (NBu_4_)[Pt_2_(C^N)_2_(*p*-MeC_6_H_4_)_2_(μ-CN)] (**7**–**9**) were easily prepared in high yields by treatment of (NBu_4_)[Pt(C^N)(*p*-MeC_6_H_4_)(CN)] (**4**–**6**) with the corresponding precursors [Pt(C^N)(*p*-MeC_6_H_4_)(SMe_2_)] (1:1 molar ratio) in acetone at 45–50 °C for 4 h. However, the attempts toward the synthesis of unsymmetrical binuclear platinum(II) complexes with various precursors such as (NBu_4_)[Pt(dfppy)(*p*-MeC_6_H_4_)(CN)] and [Pt(ppy)(*p*-MeC_6_H_4_)(SMe_2_)] and vice versa, under similar conditions, have been unsuccessful, and mixtures of possible symmetrical and unsymmetrical binuclear complexes were obtained.

**Figure 3:**
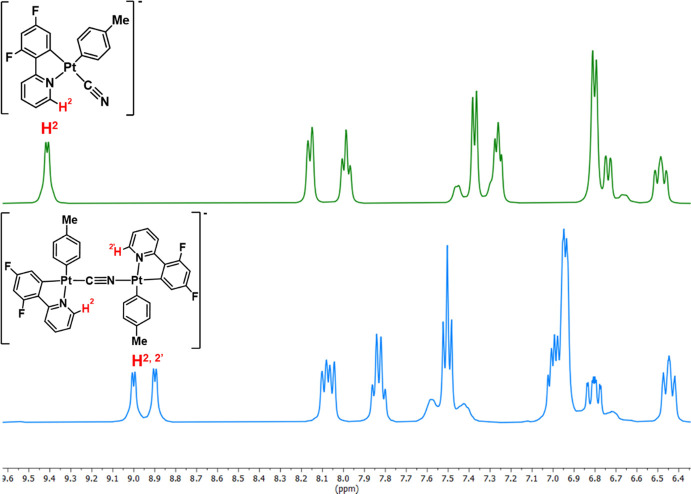
Aromatic region of the ^1^H NMR spectra of **6** (MeOD) and **9** (CD_2_Cl_2_) at 298 K.

The new complexes **7**–**9** were characterized by matrix-assisted laser desorption/ionization time-of-flight (MALDI-TOF) mass spectrometry, IR spectroscopy, 1D [^1^H, ^13^C{^1^H}, ^19^F{^1^H}, ^195^Pt] and 2D (^1^H -^1^H COSY, ^1^H - ^13^C HSQC, ^1^H- ^13^C HMBC) NMR spectroscopy, and single-crystal X-ray diffraction. The MALDI(−) mass spectra show the presence of the corresponding M^−^ molecular ion as the parent peak in all three complexes (Figure S13). The IR spectra in the solid state display the presence of one strong ν(C≡N) band shifted to higher frequencies compared to the terminal cyanide in the corresponding mononuclear complexes (2123–2130 vs 2087–2103 cm^–1^), in accordance with the bridging nature of the cyanide ligand.^54,62,63^ The ^1^H and ^13^C{^1^H} NMR spectra exhibit the presence of two sets of signals corresponding to cyclometalated ligands, clearly reflected in the low field *ortho* protons of the pyridine rings (H^2,2’^), shifted to low frequency in relation to the mononuclear precursors (Figure 3) in all three compounds, and the signals due to the tetrabutylammonium cation in the expected molar ratio. This fact is also reflected in the presence of four fluorine resonances in the ^19^F{^1^H} spectrum of complex **9** (Figure S11c). To accurately detect the carbon resonance of the cyanide ligand, the in situ formation of the related binuclear complex (NBu_4_)[Pt_2_(ppy)_2_(*p*-MeC_6_H_4_)_2_(μ-^13^CN)] in CDCl_3_, **8′**, starting from (NBu_4_)[Pt(ppy)(*p*-MeC_6_H_4_)(^13^CN)] **2′**, was carried out. The initial signal at δ 157.4 (**2′**) ppm flanked with ^195^Pt satellites (^1^*J*_Pt–C_ = 882 Hz) shifts to 154.6 in the bimetallic complex **8′**, but unfortunately, the expected two sets of platinum satellites were not resolved (Figure S10). Consistently, the ^195^Pt NMR spectra of **7**–**9** show two signals, one in the same region as the corresponding mononuclear complexes (δ −3721 to −3770), ascribed to the formally anionic Pt coordinated to the C_CN_, and the second, downfield-shifted (δ −3515 to −3578), corresponding to the formally neutral Pt coordinated to the N_CN_.

**Figure 4:**
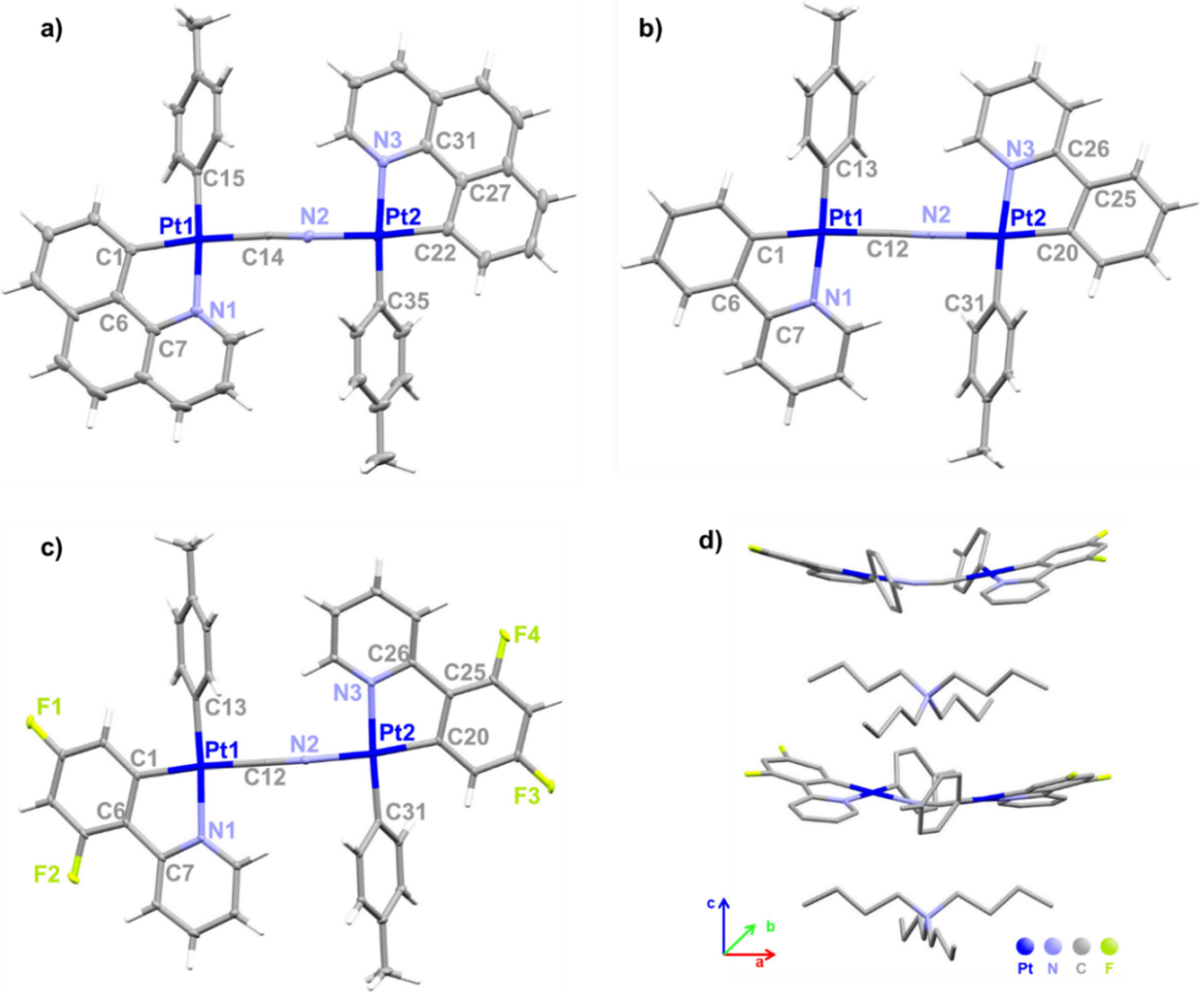
Molecular structures of the binuclear platinum(II) complex anions for (a) **7**, (b) **8**, and (c) **9** and (d) packing structure of complex **9** illustrating the alternation of cations and anions.

The X-ray diffraction analysis was used to confirm the structure of the complexes **7**–**9**. Suitable crystals were obtained by slow diffusion of *n-*hexane into an acetone solution of the corresponding complex. The plot of the structures is shown in Figure 4, whereas the obtained values of the main bond lengths and bond angles are shown in Table 3. As shown in Figure 4, the results of the X-ray analysis confirm the bimetallic nature of the anion in complexes **7**–**9**, in which two similar [Pt(C^N)(*p*-MeC_6_H_4_)] units adopting an *anti*-conformation are connected by a bridging cyanide ligand. The torsion angles between two platinum planes [Pt (1)–C_cyanide_–N (2)–Pt (2)] are 42.74° in **7**, 33.18° in **8**, and 12.39° in **9**. In each complex, the Pt(1)–C_C^N_ [1.947 (2)–2.008 (4) Å] distance, *trans* to the C≡N, is identical within the experimental error to the corresponding Pt(2)–C_C^N_ [1.948 (2)–2.016 (4) Å] distances *trans* to the N≡C and, in average, slightly shorter to those found in mononuclear complexes [2.179 (2) **5**, 2.030 (3) Å **6**]. As expected, the bond lengths of bridging CN^−^ [1.144 (3) **8**, 1.111 (3) Å **9**] are slightly shorter to those seen for the terminal cyanide in the mononuclear complexes [1.236 (3) **5**, 1.155 (3) Å **6**] and comparable to those reported in square platinum complexes with μ-CN^−^ ligands.^64,65^ The C^N ligand bite angles (81.25°, 80.88° **7**, 79.28°, 79.24° **8**; 81.91°, 81.93° **9**) are comparable to those seen in **5** and **6**. In the crystal packing, the anions are separated from each other with the bulky tetrabutylammonium cations interacting through weak C–H···π interactions (2.697–2.968 Å) (Figures 4d and S15).

**Table 3:** Selected Distances (Å) and Angles (°) for Binuclear Complexes **7**–**9**

parameter	**7**	**8**	**9**
Pt(1)–C_C^N_	2.008(4)	2.008(3)	1.947(2)
Pt(1)–N(1)	2.116(3)	2.038(2)	2.0760(19)
Pt(1)–C_cyanide_	2.029(3)	2.016(2)	1.9586 (19)
Pt(1)–C_tol_	2.017(3)	1.949(2)	1.996(2)
C≡N	1.152(4)	1.144(3)	1.111(3)
Pt(2)–C_C^N′_	2.016(4)	2.007(3)	1.948(2)
Pt(2)–N(3)	2.108(3)	2.038(2)	2.0794(19)
Pt(2)–N(2)	2.029(3)	2.021(2)	1.960(2)
Pt(2)–C_tol′_	2.018(4)	1.948(2)	1.997(2)
C_C^N_-Pt(1)–N(1)	81.26(13)	79.28(10)	81.91(9)
C_C^N′_-Pt(2)–N(3)	80.90(15)	79.25(10)	81.93(9)
Pt(1)–C_cyanide_–N(2)–Pt(2)	42.74	33.18	12.39

### Photophysical Studies and Theoretical Calculations

#### Absorption Spectra

**Figure 5:**
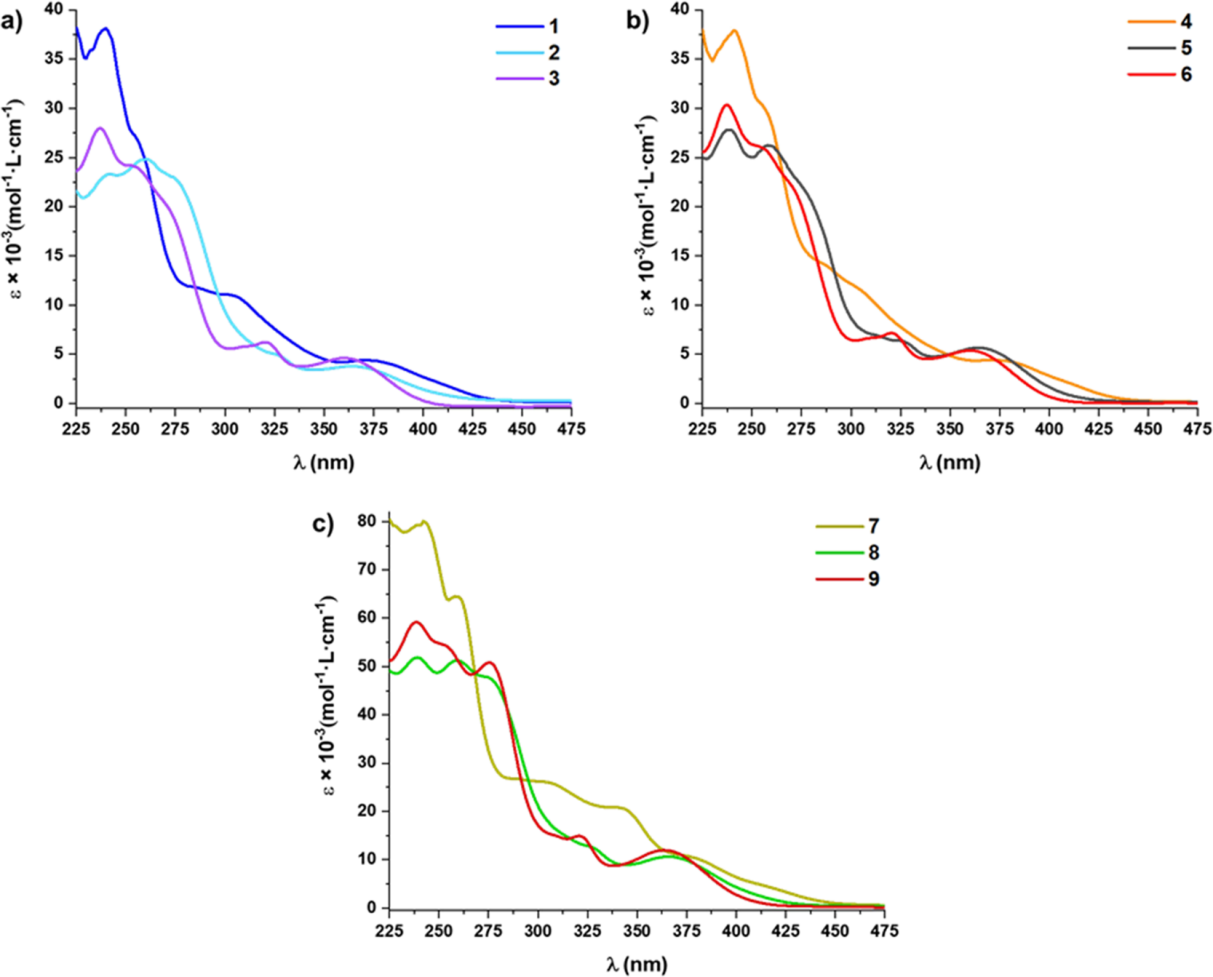
UV–vis absorption spectra of (a) **1**–**3**, (b) **4**–**6** in MeOH, and (c) **7**–**9** (MeOH/CH_2_Cl_2_) (5 × 10^–5^ M) at 298 K.

**Figure 6:**
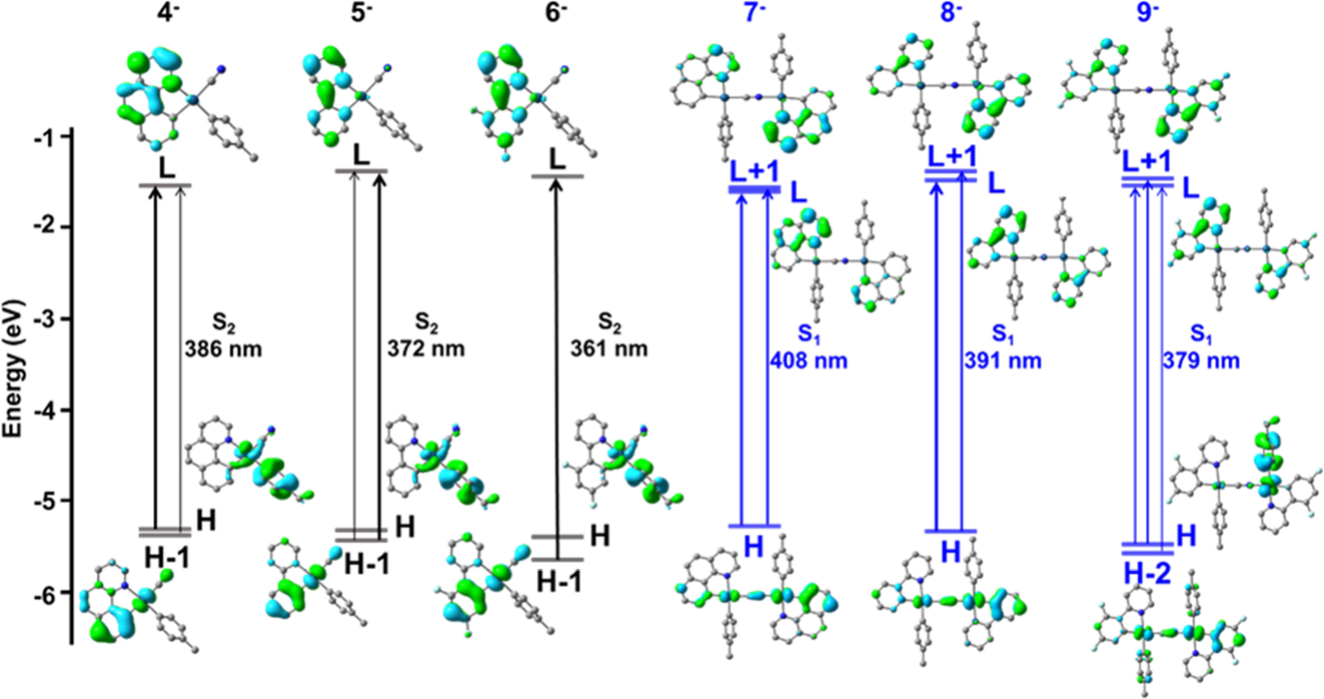
Schematic representation of selected frontier orbitals and excitations of the anions **4^–^**–**9^–^**. Transitions with the highest *f* (oscillator strength) in the LE region.

The UV–vis absorption spectra of mononuclear complexes were recorded in MeOH (5 × 10^–5^ M) and of those binuclear complexes, due to solubility reasons, in a mixture of MeOH/CH_2_Cl_2_ (80/20) at 298 K. The data are summarized in Table S2, and the spectra are given in Figure 5. As can be seen, the UV–vis spectra of **1**–**6** containing the same cyclometalated ligand are essentially identical (Figure 5a,b), indicating the negligible influence of the cation on the electronic transitions. Therefore, only the corresponding anionic unit has been considered for the DFT and TD-DFT calculations [MeOH and gas phase (not included)]. Consistent with previous assignments in analogous cycloplatinated complexes, the high energy bands (204–330 nm) are attributed to metal-perturbed π–π* ligand-centered transitions (^1^LC) located on the C^N and *p*-MeC_6_H_4_ ligands.^24,27,51,59,66−68^ According to TD-DFT calculations, the less intense energy bands appearing at λ > 330 nm have mostly contributions from admixture ^1^LC (C^N)/^1^MLCT [dσ(Pt) → π*(C^N)] and ^1^L′LCT [π(*p-*MeC_6_H_4_) → π*(C^N)] transitions. The cyanide ligand has a minor influence on the absorption features. Thus, in accordance with the larger delocalization and stabilization of the target orbital, in complexes with bzq ligand (**1**, **4**) the LE absorption is red-shifted in relation to complexes with ppy (**2**, **5**).^51,60,69−71^ However, in complexes **3** and **6**, the absorption maximum is blue-shifted in relation to the non-fluorinated-ppy in complexes **2** and **5**, due to the stabilization of the highest occupied molecular orbital (HOMO), which provokes a larger band gap (Figure 6).

In the binuclear complexes (**7**–**9**), the lowest-energy absorptions appear subtle red-shifted and exhibit a higher extinction coefficient compared with the mononuclear analogues (Figure 5c), a fact attributed to the incorporation of two chromophores which are involved electronically.^72^ In the binuclear complexes **7** and **8**, the LE absorption has mainly ^1^LC/^1^MLCT admixture with ^1^L″LCT [π(CN^−^) → π*(C^N)] contribution, whereas in the case of **9** has mixed ^1^LC/^1^MLCT/^1^L′LCT [π(*p-*MeC_6_H_4_) → π*(C^N)] configuration. The calculations suggest that the bimetallic Pt–CN–Pt unit enables an increase of the ^1^MLCT contribution in the LE band along with a decrease of the ^1^LC contribution in relation to the monomers.

Energy level diagrams of selected frontier molecular orbitals (FMOs) in the ground state together with the most intense LE excitations are shown in Figure 6. The lowest-energy transitions of the anionic mononuclear complexes (**1**–**6**) are ascribed to the HOMO→LUMO and HOMO-1 → LUMO transitions. The main contribution to the HOMOs comes from the *p*-MeC_6_H_4_ group (66% **1**, **4**; 67% **2**, **5**; 70% **3**, **6**) and the platinum (25% **1**, **4**; 24% **2**, **5**; 23% **3**, **6**), whereas the HOMOs-1 are constructed from the cyclometalating ligand (62% **1**, **4**; 52% **2**, **5**; 49% **3**, **6**) and d(Pt) orbitals (31% **1**, **4**; 39% **2**, **5**; 42% **3**, **6**) with minor participation of CN^−^ (6% **1**, **4**; 8% **2**, **5**; 9% **3**, **6**) (Tables S3 and S4 and Figure S16). As the lowest unoccupied molecular orbitals (LUMOs) have dominant contributions from π* orbitals of the cyclometalated ligand (95% bzq; 91% ppy; 90% dfppy), the lowest-energy absorptions possess mixed transitions ^1^LC/^1^MLCT/^1^L′LCT (L = C^N; L′ = *p*-MeC_6_H_4_) with negligible ^1^L″LCT (L″ = CN^−^) character.

In the binuclear complexes (**7**–**9**), the lowest-energy absorption peak was essentially induced by the transitions from HOMO→LUMO and HOMO→LUMO + 1, although in **9**, the HOMO-2 → LUMO configuration is also important (Tables S5 and S6 and Figure S17**)**. In **7** and **8**, the HOMOs are delocalized on both Pt-cyclometalating units with contribution of the cyanide (C^N/Pt/CN^−^: 55/39/5% bzq/Pt/CN^−^**7**; 46/47/6% ppy/Pt/CN^−^**8**), while in **9**, the HOMO and HOMO-1, which are nearly degenerated, localize the electron density over the two Pt-(*p*-MeC_6_H_4_) units and the cyanide linker (*p*-MeC_6_H_4_/Pt/CN^−^/dfppy 59/31/2/8%). The LUMOs and LUMOs + 1 are delocalized over the two cyclometalated ligands; therefore, the lowest-energy absorptions of binuclear complexes **7** and **8** can be assigned to a ^1^LC/^1^MLCT admixture with reduced participation of L″LCT (L″ = CN^−^), whereas complex **9**, as in the mononuclear precursor **6**, has an important ^1^L′LCT (L′ = *p*-MeC_6_H_4_) character.

The solid diffuse reflectance UV–vis spectra of compounds (**1**–**9**) have been also examined, and they are depicted in Figure S18 and Table S2. The LE absorption band, responsible for their colors, are red-shifted when compared to solution, following a similar tendency to those found in solution. The pristine solid **9** (Figure S18c) revealed blue-shifted band up to 465 nm in relation to the pristine solids **7** and **8**, with tails extending to 490 nm, in coherence with their color and in accordance with the calculations.

### Emission Spectra

**Figure 7:**
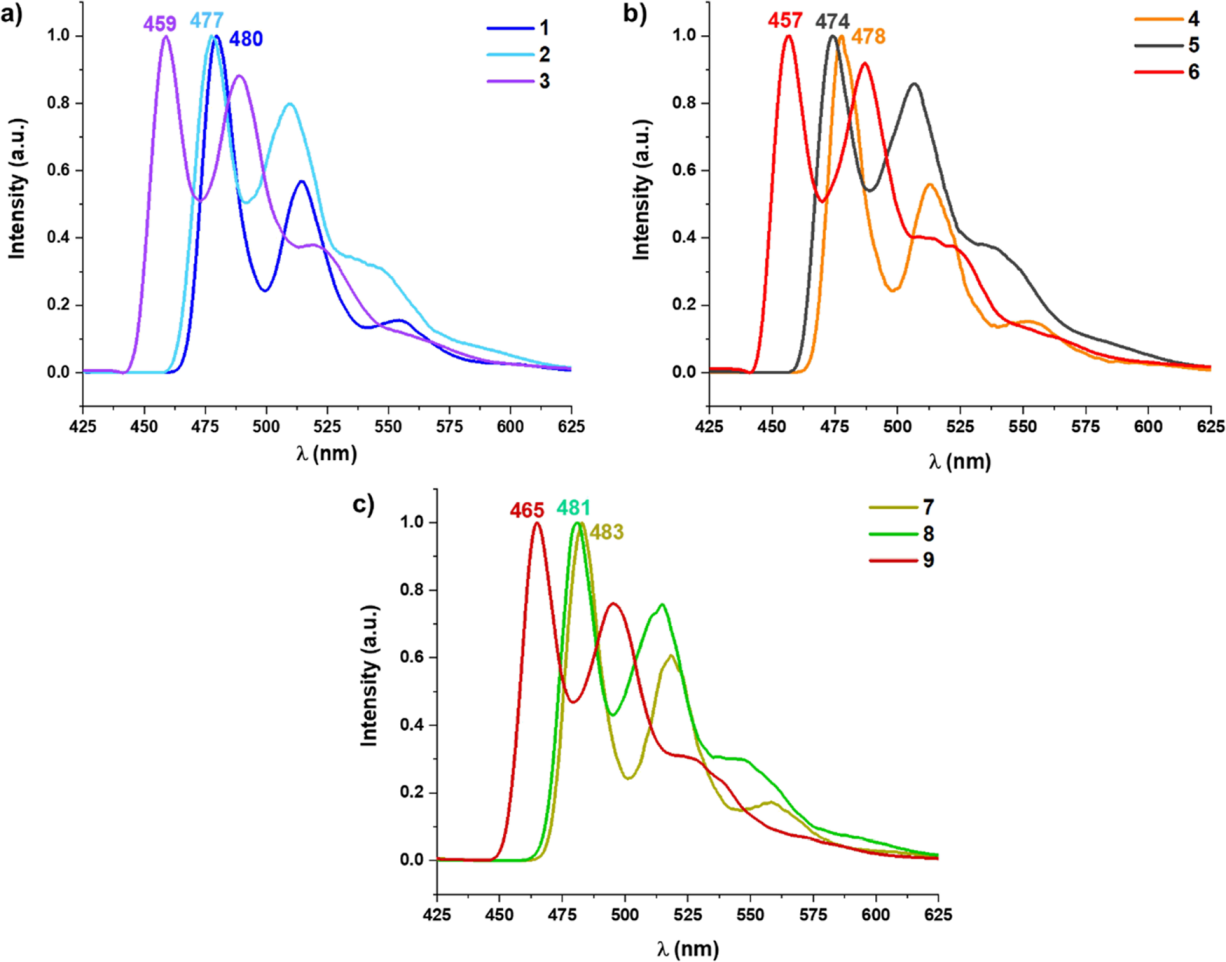
Normalized emission spectra of (a) **1**–**3**, (b) **4**–**6** in MeOH, and (c) **7**–**9** in MeOH/CH_2_Cl_2_ (5 × 10^–5^ M) at 77 K.

The photoluminescence emission properties of all complexes were studied in deoxygenated solution at 298 and 77 K (MeOH, **1**–**6**; MeOH/CH_2_Cl_2_**7**–**9**) in a doped polystyrene (PS) matrix (1 wt % for **1**–**3** and **7**–**9**, 1–20 wt % for **4**–**6**) at 298 K and in the solid state at 298 and 77 K. The photophysical data, upon excitation at 365 nm, are summarized in Table 4, representative spectra are provided in Figures 7 and S19–S22, and lifetimes decays are shown in Figures S23–S31. The complexes are not emissive in fluid solution at room temperature, probably due to an easy quenching process of the excited states caused by (a) molecular motions in the solution, (b) collisional interactions with solvent molecules, (c) energy transfer to triplet ^3^O_2_ facilitated by the long lifetimes, or (d) thermally activated deactivation through the ^3^MC excited state.^73^ However, all complexes exhibit strong luminescence in the rigid matrix (glassy solution and PS) and in the solid state with lifetimes in the range of microsecond (μs) regime, confirming the involvement of the triplet excited state in the emission.^74,75^ The potassium (**1**–**3**) and tetrabutylammonium derivatives (**4**–**6**) exhibit similar structured emission bands in glassy solution and PS film, the latter being slightly red-shifted, suggesting some interactions between relatively well-separated ions. The emissions in glassy solutions do not reveal any systematic dependence on the solvent (Figure S19 for **6** and **9**). These emissions are typical of metal-perturbed C^N ^3^(π → π*) excited states and, accordingly, the energy peak maximum is subtly red-shifted on going from dfppy to ppy and to bzq derivatives (MeOH, 77 K: 456 **6**; 474 **5**; 477 nm **4** and 458 **3**; 478 **2**; 479 nm **1**), in line with the more delocalized bzq target ligand and the presence of electron-acceptor fluorine atoms in the phenyl ring.^31,43,57,76,77^ According to DFT calculations,^78,79^ these emissions are ascribed to ^3^LC with ^3^MLCT character. For the bzq complexes, the lifetimes are extremely longer than those measured for the ppy and dfppy (MeOH 77 K, 323.2 **1**; 289.9 μs **4**). This fact has been previously observed by us in other Pt(bzq) compounds^80^ and can be attributed to smaller spin–orbit coupling due to lower platinum contribution into the excited state and also to the stronger structural rigidity of the bzq compared to arylpyridinate cyclometalated groups, which inhibits nonradiative decay. In accordance, the calculated values of *K*_nr_ in PS are significantly lower in the benzoquinolate complexes **1** and **4** compared to the related **2**, **3** and **4**, **5** (Table 4). The quantum yields measured in PS range from 3.3 to 16.2% for the potassium derivatives (**1**–**3**) and from 2.3 to 8.6% for the tetrabutylammonium compounds (**4**–**6**) without a clear tendency between them.

**Table 4:** Photoluminescence Properties of Complexes **1**–**9**

compound	medium	*T*(K)	λ_max_/nm	Φ_PL_	τ/μs	*k*_r_^a^/s^–1^	*k*_nr_^b^/s^–1^
**1**	Solid	298	500_sh_, 593,635_sh_	2.5	74.8 (28%), 17.3 (72%)	7.5 × 10^2^	2.9 × 10^4^
		77	504		26.2 (45%), 152.1 (55%)		
	PS^c^	298	490	3.3	48.0 (36%), 17.1 (64%)	11.7 × 10^2^	3.4 × 10^4^
	MeOH	77	479		323.2		
**2**	Solid	298	495 [365]	1.8	0.2 (76%), 1.2 (24%) [500]	4.1 × 10^4^	2.2 × 10^6^
			575 [490]	2.3	0.1 (78%), 1.0 (22%) [575]	7.7 × 10^4^	3.3 × 10^6^
		77	494		11.4		
	PS^c^	298	486	16.2	0.5 (66%), 5.6 (34%)	7.3 × 10^4^	3.8 × 10^5^
	MeOH	77	478		13.8		
**3**	Solid	298	470	0.7	0.1 (78%), 1.4 (22%)	1.8 × 10^4^	2.6 × 10^6^
		77	470		10.7		
	PS^c^	298	469	12.5	0.6 (66%), 4.5 (34%)	6.5 × 10^4^	4.5 × 10^5^
	MeOH	77	458		16.5		
**4**	Solid	298	490	3.9	0.1 (60%), 0.9 (40%)	9.3 × 10^4^	2.3 × 10^6^
		77	497		124.4 (38%), 50.9 (62%)		
	PS^d^	298	503	8.6	0.6 (81%), 7.5 (19%)	4.5 × 10^4^	4.8 × 10^5^
	MeOH	77	477		289.9		
**5**	Solid	298	489	19.4	0.02 (64%), 0.5 (36%)	10.1 × 10^5^	4.2 × 10^6^
		77	494		14.2		
	PS^d^	298	494	2.3	4.7 (10%), 0.5 (89%)	2.5 × 10^4^	1.1 × 10^6^
	MeOH	77	474		15.7		
**6**	Solid	298	471	13.3	0.4 (53%), 3.7 (47%)	6.8 × 10^4^	4.4 × 10^5^
		77	474		10.7		
	PS^d^	298	476	6.3	0.3 (78%), 3.0 (22%)	7.0 × 10^4^	1.0 × 10^6^
	MeOH	77	456		15.3		
**7**	Solid	298	496, 577	3.4	0.29 (80%), 2.3 (20%) [496]	4.9 × 10^4^	1.4 × 10^6^
					15.2 [577]	2.2 × 10^3^	6.4 × 10^4^
		77	513		26.5 (82%), 75.8 (18%)		
	PS^c^	298	496, 575^br^	7.4	15.3	4.8 × 10^3^	6.1 × 10^4^
	MeOH	77	482		173.5		
**8**	Solid	298	484	3.5	0.3 (52%), 0.99 (48%)	5.5 × 10^4^	1.5 × 10^6^
		77	489		9.8		
	PS^c^	298	496	8.6	0.24 (78%), 3.6 (22%)	8.8 × 10^4^	9.3 × 10^5^
	MeOH	77	480		13.8		
**9**	Solid	298	469	21.0	0.54 (21%), 2.2 (79%)	1.1 × 10^5^	4.3 × 10^5^
		77	461		9.3		
	PS^c^	298	478	20.0	0.5 (49%), 2.9 (51%)	1.2 × 10^5^	4.6 × 10^5^
	MeOH	77	464		14.3		

a *k*_r_ = ϕ/τ_average_.

b *k*_nr_ = (1 – ϕ)/τ_average_.

c 1 wt % in polystyrene film.

d 1–20 wt % in polystyrene film.

**Figure 8:**
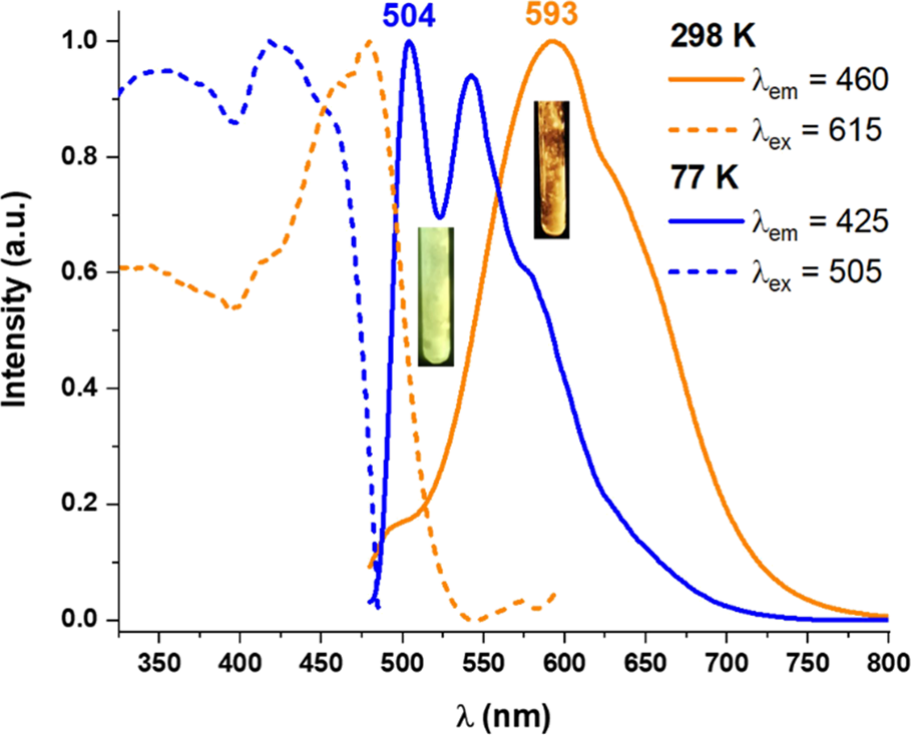
Thermochromic behavior of **1** in the solid state.

The most notable difference between the potassium and tetrabutylammonium derivatives was found in the solid state. Thus, all tetrabutylammonium complexes **4**–**6** exhibit similar monomer emissions to those seen in PS and glassy state coming from isolated chromophores, likely due to the presence of a bulky NBu_4_^+^ cation, which prevents intermolecular Pt···Pt and/or π–π interactions, as has been previously observed in related anionic complexes.^43,44,47,51,81^ In the case of potassium compounds, only K[Pt(dfppy)(*p-*MeC_6_H_4_)(CN)] (**3**), featuring the bulkier F substituents, displays monomer emission, as a vibronic band at λ_max_ 470 nm and with lower quantum yield to that seen in PS matrix (ϕ 0.7 solid vs 12.5 PS), suggesting some quenching behavior. By contrast, complex **1** displays luminescence thermochromism, with a remarkable blue shift upon cooling due to the presence of two distinct emission bands (Figures 8 and S21). Thus, at 298 K, solid complex **1** exhibits a main broad long-lived (τ_av_ 33.3 μs) LE orange emission (λ_max_ 593 nm), which is ascribed to an excimer-like excited state associated to the occurrence of Pt···Pt/π–π intermolecular interactions in the solid state, as frequently observed for neutral cycloplatinated complexes.^16,24^ A small high-energy (HE) shoulder at 500 nm, associated to ^3^LC excited state of the monomer, is also detected. Upon cooling to 77 K, a remarkable blue shift is visually observed with a change to a green emission due to the presence of the structured HE emission (λ_max_ 504 nm) with an extremely long-life time (τ_av_ 95.5 μs). As is usual in this type of systems, thermally induced switching of the luminescence is governed by the controllable population of two different excited states.^82^ At room temperature, the thermal energy (*kT*) seems to be higher than the energy barrier (*E*_a_) between both HE and LE excited states, allowing the population of the LE excited state, which is also favored by the relatively long lifetime. At low temperature (77 K), the thermal energy is not enough to pass the *E*_a_ barrier, which finally prevents populating the LE excited state. For complex **2** (see Figure S22), its emission profile depends on the excitation wavelength, which is indicative of site heterogeneity. By excitation at λ ∼ 365 nm, solid **2** displays a broad green-yellow emission attributed to monomer (495 nm) with contribution from LE excimer emission (575 nm), which is primarily developed by exciting at lower wavelength. Unfortunately, complexes **1**–**3** do not exhibit vapochromism or vapoluminescent behavior upon Et_2_O, CHCl_3_, CH_2_Cl_2_, or acetone fuming or solvatochromic effect by treatment of the solids with a drop of these solvents and drying, in contrast with the strong vapochromic behavior of some potassium-cycloplatinated compounds previously reported.^51−53^

**Figure 9:**
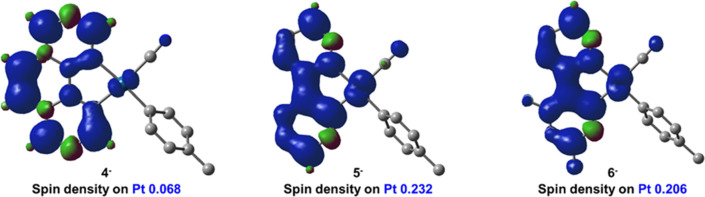
Spin distribution for the lowest triplet excited state in the mononuclear anions **4^–^**–**6^–^**.

The nature of the emissions in the mononuclear complexes was investigated via TD-DFT calculations of the lowest triplet excited-state (T_1_), for the T_1_ → S_0_ transition, and the spin density distribution, which was fully optimized based on the geometry of S_0_ and T_1_, starting from X-ray structures. The SOMOs-1 differ from the HOMOs being located on the C^N with remarkable contribution of the Pt and a negligible participation of the cyanide and the tolyl groups. The SOMOs in all mononuclear anions are formed by the C^N ligands, thus supporting emission of ^3^LC nature with minor ^3^MLCT (L = C^N) character. The spin density distributions of the three anions are depicted in Figure 9 and reveal that the lowest Pt contribution occurs in the anion with benzoquinolate ligand and the highest in [Pt(ppy)(*p*-MeC_6_H_4_)(CN)]^−^ (11.6% ppy **2**, **5** > 10.3% dfppy **3**, **6** > 3.4% bzq **1**, **4**); therefore, the contribution of the MLCT follows the tendency **1**, **4** < **3**, **6** < **2**, **5**. The calculated emission wavelengths [**3^–^**, **6^–^** (515) < **2^–^**, **5^–^** (537) < **1^–^**, **4^–^** (553 nm)] (Table S9) correlate well with the observed emission in glassy solution and PS [glass **3**, **6** (458) 456 < **2**, **5** (478) 474 < **1**, **4** (479) 477 nm].

The emission spectra of the binuclear complexes **7**–**9** in glassy solution (MeOH-CH_2_Cl_2_, Figure 7c) show a similar trend to those of the mononuclear **4**–**6**, with the energy of the emission depending on the cyclometalated ligand (glass, 77 K: λ_max_ 465 **9** dfppy >480 **8** ppy > 492 nm **7** bzq), being slightly red-shifted in comparison to the mononuclear species (Figure 7). This fact points to a similar ^3^LC/^3^MLCT emissive state with predominantly ^3^LC character, which is further supported by calculations. Similar structured emissions due to monomer species were observed for complexes **8** and **9** in diluted PS films (1 wt %), whereas the benzoquinolate complex **7** showed, in addition to the monomer (496 nm), an LE feature (∼575 nm) due to aggregates species in a low contribution (Figure S20c). Even in the presence of bulky NBu_4_^+^ counterions, the tendency to form aggregates for **7** is clearly reflected in the solid state at room temperature, as this complex exhibits an emissive profile with a major LE excimer like band at 577 nm (Figure S22). However, upon decreasing the temperature to 77 K, the thermal energy is not enough to pass the *E*_a_ barrier, preventing the access to this LE excited state, and thus the complex shows only the structured monomer emission of ^3^LC nature. For the dfppy complex **9**, the quantum yield is clearly enhanced (ϕ ∼ 20–21% in solid and PS) in relation to the precursor monomer **6** (ϕ 13% solid and 6% PS). A similar tendency is observed in the corresponding value of *K*_r_ and *K*_nr_ in PS, which increases and decreases, respectively, in **9** in relation to **6** (Table 4). However, no similar effect is observed for complexes **7** (solid and PS) and **8** (solid), which display efficiencies similar to those of the precursors **4** and **5**, although for the bzq complex **7**, the *K*_r_ and *K*_nr_ decrease one order in relation to **4**.

**Figure 10:**
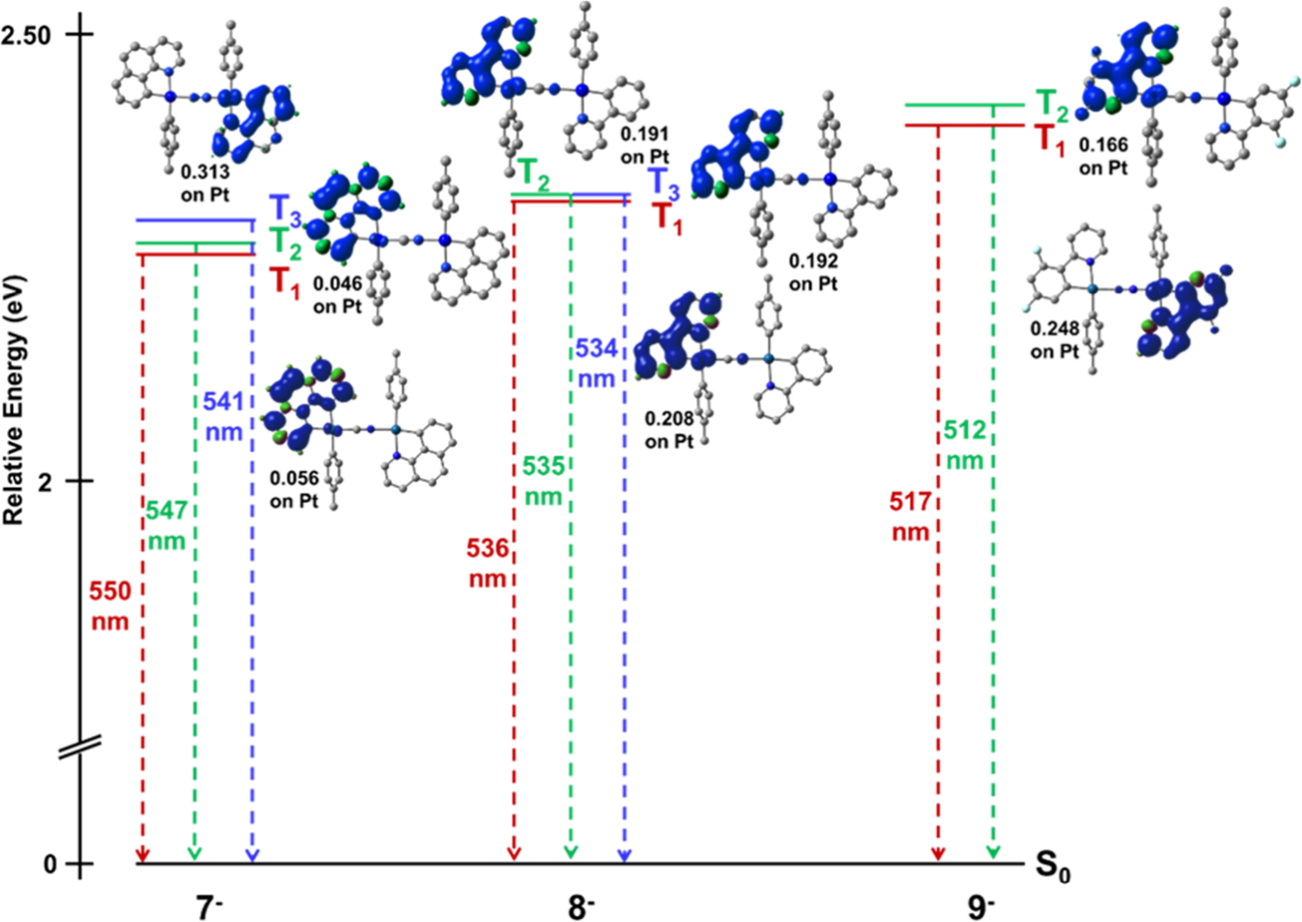
Spin density distribution and relative energies for the triplet excited states T_1_–T_3_ in the binuclear anions **7^–^**–**9^–^**.

**Figure 11:**
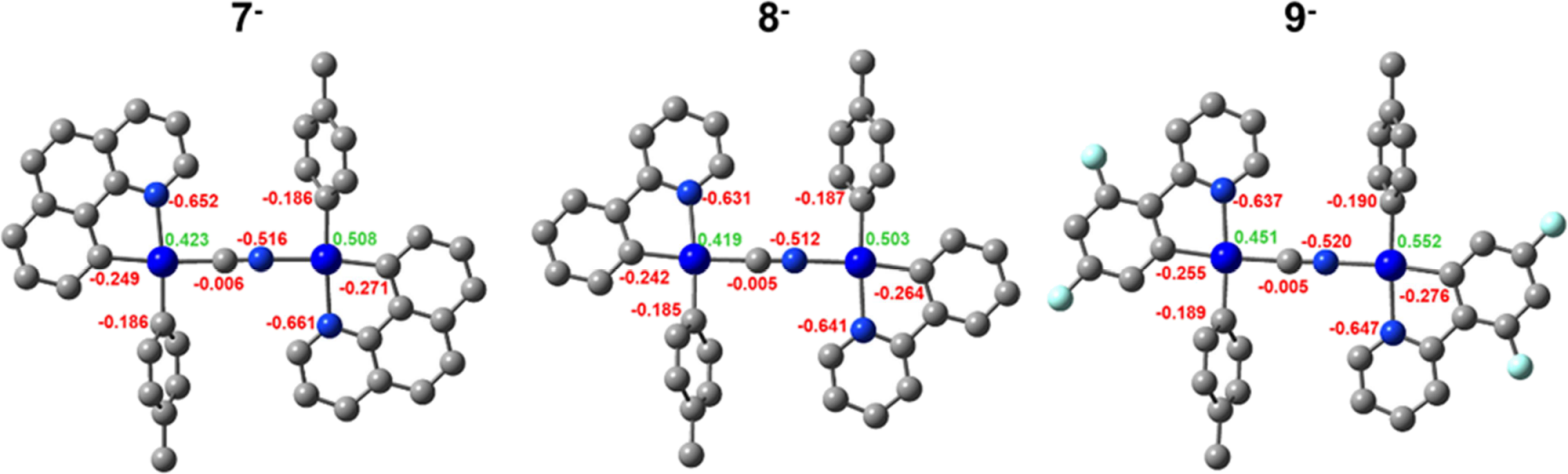
Mulliken Charge Distribution in the optimized S_0_ state for the binuclear anions **7^–^−9^–^**.

The nature of the monomer emissions was examined through the calculation of the spin density distribution for the triplet excited state (T_1_) based on its corresponding optimized T_1_ geometry. In contrast to the HOMO and LUMO, which are contributed from the two Pt units, upon excitation, the electron density moves and the composition of the spin density of the low-lying triplet emissive state (Figure 10) is mainly localized on one of the platinum chromophores. The composition of the SOMO and SOMO-1 in the anions of complexes **7** and **8** is mainly contributed by the Pt(C^N) unit located *trans* to the C of the cyanide bridge, whereas **9^–^** is formed from orbitals of the Pt(dfppy) *trans* to the N of the cyanide (Table S8). The SOMO-1 has a minor contribution of the cyanide bridge (1% **7**, **9**; 2% **8**). Aiming to understand the difference in the localization of the SOMO in **9** in relation to **7** and **8** in T_1_, the two following triplets T_2_ and T_3_ were optimized and the distribution of charge in the S_0_ was also calculated. As can be seen in Figure 10, the close T_2_-excited state is similar in all complexes, being located in the anionic platinum fragment *trans* to the C_CN_ bridging. For the ppy complex **8**, T_2_ and T_3_ are nearly degenerate and close to T_1_, being also located on the anionic (ppy)Pt fragment located *trans* to the C_CN_, whereas in the bzq derivative **7**, T_2_ is similar to T_1_ but T_3,_ only 302 cm^–1^ above T_1,_ is focused on the neutral (bzq)Pt *trans* to the N_CN_. This result indicates that these excited states are very close in energy. For **9**, the T_3_ is very distorted due to high Pt contribution and, therefore, is not considered. The reason why T_1_ is located on the formally neutral fragment in complex **9** is not clear but could be related to the greater positive charge difference between the formally neutral and anionic platinum center (0.085 in **7**, 0.084 **8**; 0.101 **9**), which likely stabilizes the neutral fragment (Figure 11).

## Conclusions

In summary, two series of photoluminescent mono- and binuclear anionic heteroleptic cyclometalated platinum(II) complexes, Q^+^[Pt(C^N)(*p*-MeC_6_H_4_)(CN)]^−^ (Q^+^ = K^+^ and NBu_4_^+^) and (NBu_4_)[Pt_2_(C^N)_2_(*p*-MeC_6_H_4_)_2_(μ-CN)], respectively, incorporating different cyclometalated ligands [C^N = 2-phenylpyridinate (ppy), benzoquinolate (bzq), dfppy = 2-(2,4- difluorophenyl)pyridinate] and featuring terminal and bridging cyanide ligand are reported. The bimetallic derivatives represent one of the scarce examples of mono-bridged CN^−^ binuclear Pt complexes. The tetrabutylammonium complexes have been characterized by X-ray diffraction studies. Attempts to obtain monocrystals for the potassium derivatives have been unsuccessful and, surprisingly, slow crystallization of the ppy complex **2** evolves with partial elimination of KCN, giving rise to the salt complex (**10**) featuring the diplatinum anionic unit [Pt(ppy)(*p*-MeC_6_H_4_)(μ-CN)Pt(ppy)(*p*-MeC_6_H_4_)]^−^ neutralized by an hybrid inorganic–organometallic coordination polymer {[Pt(ppy)(*p*-MeC_6_H_4_)(CN)]_2_K_3_(OCMe_2_)_4_(μ-OCMe_2_)_2_}^+^.

These complexes are not emissive in fluid solution but display moderated efficiencies in rigid media, with ϕ up to 20% in PS film, attributed to mixed ^3^LC/^3^MLCT, in which the contribution of the MLCT mainly depends on the cyclometalated ligand following the tendency bzq < dfppy < ppy. The analysis of the photoluminescent properties and TD-DFT calculations indicates that the role of the cyanide ligand in the electronic transitions seems to be relatively small. In the solid state, the potassium complexes **1** and **2** show tendency to form aggregates, particularly enhanced in the bzq compound **1**, which leads to a clear thermochromic behavior due to a switch on of an LE emission at room temperature, which is prevented at 77 K.

This work opens a new avenue to the design of anionic cycloplatinated complexes, other than the more well-studied neutral [Pt(C^N)XL] systems. Finally, and not least important, we offer a method to prepare anionic diplatinum cyanide cyclometalated complexes, which can be very efficient as synthons for creating novel complexes of heteropolynuclear systems. This is currently being conducted in the research group and it is envisaged to revive the interest for the synthesis of new types of supramolecular extended networks.

## Experimental Section

### General Comments

The precursor complexes [Pt(C^N)(*p*-MeC_6_H_4_)(SMe_2_)] [C^N = benzoquinolate (bzq), phenylpyridinate (ppy), 2-(2,4-difluorophenyl)pyridinate) (dfppy)] were prepared according to literature procedures.^56,57,83^ The microanalyses were carried out with a EA FLASH 2000 (Thermo Fisher Scientific) or a Perkin-Elmer CHNS/O 2400 Series II elemental analyzer. The IR spectra were measured using a PerkinElmer Spectrum UATR Two FT-IR Spectrometer with the diamond crystal ATR accessory (ATR in the range of 400–4000 cm^–1^). Electrospray ionization mass spectrometry (ESI-MS) measurements were recorded by electrospray ionization on a Bruker Microtof-Q spectrometer in the negative ion mode in MeOH (**1–3**) and in CH_2_Cl_2_ (**4–6**) and a Microflex MALDI-TOF Bruker spectrometer in the negative ion mode in CH_2_Cl_2_ (**7–9**). The solution NMR spectra were recorded on a Bruker Avance ARX 400 MHz spectrometer at 298 K. The chemical shifts (δ) relative to external standards (TMS for ^1^H and ^13^C{^1^H}, CFCl_3_ for ^19^F{^1^H} and K_2_PtCl_4_ in D_2_O for ^195^Pt) and coupling constant or *J* constant were expressed in units of parts per million (ppm) and hertz (Hz), respectively. The UV–vis absorption spectra were measured with a Hewlett-Packard 8453 spectrophotometer. Diffuse reflectance UV–vis spectra were carried out in SiO_2_ pellets, using a Shimazdu UV-3600 spectrophotometer with a Harrick Praying Mantis accessory, and recalculated following the Kubelka–Munk function. Excitation and emission spectra were obtained in a Shimazdu RF-60000. The measurements in solid state and PS films were carried out on air and in solutions under a N_2_ atmosphere. The lifetime measurements up to 10 μs at 298 K at all samples at 77 K were performed with a Jobin Yvon Horiba Fluorolog operating in the phosphorimeter mode (with an F1-1029 lifetime emission PMT assembly, using a 450 W Xe lamp) and the Jobin Yvon software packing, which works with Origin 6.0. The decay data were analyzed by tail fitting to the functions “One-phase exponential decay function with time constant parameter” (ExpDec1) and “Two-phase exponential decay function with time constant parameters (ExpDec2).” The lifetimes below 10 μs at 298 K were measured with a Datastation HUB-B with a nanoLED controller, using the technique “Time Correlated Single Photon Counting” (TCSPC). Quantum yields were measured using a Hamamatsu Absolute PL Quantum Yield Measurement System C11347-11.

### Synthesis of K[Pt(bzq)(*p*-MeC_6_H_4_)(CN)] (**1**)

KCN (26 mg, 0.39 mmol) was added to a suspension of [Pt(bzq)(*p*-MeC_6_H_4_)(SMe_2_)] (211 mg, 0.40 mmol) in a mixture of MeOH/H_2_O (10/2 mL). After 4 h stirring at room temperature, MeOH solvent was evaporated under vacuum, then the mixture was filtered through celite, and the yellow aqueous resulting solution was evaporated to dryness. The residue was washed with Et_2_O (2 × 10 mL) to give a yellow solid identified as **1**·2H_2_O (analysis and NMR in DMSO, data not shown) (total yield: 169 mg, 79%). Elem. Anal. Calcd for C_21_H_15_KN_2_Pt (529.55): C, 47.63; H, 2.86; N, 5.29. Found: C, 47.32; H, 3.03; N, 4.98; IR (ν(CN), cm^–1^): 2087. ESI-MS(−): *m/z* (%): 490.08 [Pt(bzq)(*p*-MeC_6_H_4_)(CN)]^−^ (100), 954.17 ([Pt_2_(bzq)_2_(*p*-MeC_6_H_4_)_2_(CN)]^−^ (15.5); ^1^H NMR [400 MHz, MeOD, δ]: 9.55 (d, ^3^*J*_H–H_ = 5, ^3^*J*_Pt–H_ = 23, H^2^), 8.41 (d, ^3^*J*_H–H_ = 8, H^4^), 7.74 (d, ^3^*J*_H–H_ = 8.5, H^5^), 7.57 (d, ^3^*J*_Pt–Ho_ = 68.9, 2H^*o*^), 7.60–7.51 (m, 3H, H^3,6,7^) 7.47 (d, ^3^*J*_H–H_ = 8, H^9^), 7.41 (t, ^3^*J*_H–H_ = 8, H^8^) 6.85 (d, ^3^*J*_H–H_ = 7.5, 2H^*m*^), 2.27 (s, 3H, Me); ^13^C{^1^H} NMR [100.6 MHz, MeOD, δ]: 164.0 (s, ^1^*J*_Pt–C_ = 921.3, C^10^), 157.3 (s, ^2^*J*_Pt–C_ = 63.8, C^11^), 156.5 (s, CN^−^), 151.3 (s, ^2^*J*_Pt–C_ = 28.1, C^2^), 146.1 (s, ^2^*J*_Pt–C_ = 11, C^13/14^), 140.4 (s, ^2^*J*_Pt–C_ = 45.6, C^*o*^), 139.1 (s, ^1^*J*_Pt–C_ = 1020, C^ipso^), 137.9 (s, C^4^), 135.9 (s, ^2^*J*_Pt–C_ = 97.3, C^9^), 134.8 (s, ^2^*J*_Pt–C_ = 29.1, C^12^), 131.0 (s), 130.6 (s, C^5^), 130.0 (s, ^3^*J*_Pt–C_ = 64, C^8^), 128.8 (s, C^*p*^), 128.5 (s, ^3^*J*_Pt–C_ = 77.9, C^*m*^), 128.1 (s, C^13/14^), 123.9 (s, C^7^), 123.1 (s, ^3^*J*_Pt–C_ = 13.7, C^3^), 122.5 (s, C^6^), 21.14 (s, Me); ^195^Pt NMR [85.6 MHz, MeOD, δ]: −3764 (m).

### Synthesis of K[Pt(ppy)(*p*-MeC_6_H_4_)(CN)] (**2**)

Complex **2** was obtained as a yellow solid following a similar procedure to complex **1** (total yield: 164 mg, 74%) starting from [Pt(ppy)(*p*-MeC_6_H_4_)(SMe_2_)] (220 mg, 0.435 mmol) and KCN (27.4 mg, 0.420 mmol). Elem. Anal. Calcd for C_19_H_15_KN_2_Pt (505.53): C, 45.14; H, 2.99; N, 5.54. Found: C, 44.96; H, 2.88; N, 5.67; IR (ν(CN), cm^–1^): 2090; ESI-MS(−): *m/z* (%): 466 [Pt(ppy)(*p*-MeC_6_H_4_)(CN)]^−^ (38); ^1^H NMR [400 MHz, MeOD, δ]: 9.31 (d, ^3^*J*_H–H_ = 4.6, ^3^*J*_Pt–H_ = 25.6, H^2^), 7.92 (m, H^4^, H^5^), 7.64 (m, H^7^), 7.44 (d, ^3^*J*_H–H_ = 7, ^3^*J*_Pt–H_ = 69.48, 2H^*o*^), 7.20 (m, H^3^, H^9^), 6.99 (m, H^6^,H^8^), 6.79 (d, ^3^*J*_H–H_ = 6.5, 2H^*m*^), 2.23 (s, 3H, Me); ^13^C{^1^H} NMR [100.6 MHz, MeOD, δ]: 168.0 (s, ^1^*J*_Pt–C_ = 70.3, C^12^), 165.3 (s, C^10^), 157.4 (s, CN^−^), 152.6 (s, ^2^*J*_Pt–C_ = 25.2, C^2^), 149.6 (s, C^11^), 140.6 (s, C^*p*^), 140.1 (s, ^2^*J*_Pt–C_ = 44.3, C^*o*^), 138.9 (s, C^4^/C^5^), 138.3 (s, C^9^), 130.5 (s, C^ipso^), 130.1 (s, C^8^), 128.4 (s, ^3^*J*_Pt–C_ = 78.1, C^*m*^), 124.1–123.8 (m, C^3^,C^6–7^), 119.7 (s, C^4^/C^5^), 21.1 (s, Me); ^195^Pt NMR [85.6 MHz, MeOD, δ]: −3736 (m).

### Synthesis of K[Pt(dfppy)(*p*-MeC_6_H_4_)(CN)] (**3**)

Complex **3** was obtained as a yellow solid (total yield: 153 mg, 75%) following the same procedure as **1** starting from [Pt(dfppy)(*p*-MeC_6_H_4_)(SMe_2_)] (200 mg, 0.37 mmol) and KCN (23.5 mg, 0.36 mmol). Elem. Anal. Calcd for C_19_H_13_F_2_KN_2_Pt (541.50): C, 42.14; H, 2.42; N, 5.17. Found: C,42.28; H, 2.79; N, 5.02; IR (ν(CN), cm^–1^): 2090; ESI-MS(−): *m/z* (%): 502 [Pt(dfppy)(*p*-MeC_6_H_4_)(CN)]^−^ (100), 411 ([Pt(dfppy)(CN)] (5.1). ^1^H NMR [400 MHz, MeOD, δ]: 9.37 (d, ^3^*J*_H–H_ = 5.4, ^3^*J*_Pt–H_ = 26.5, H^2^), 8.14 (d, ^3^*J*_H–H_ = 8.2, H^5^), 7.96 (t, ^3^*J*_H–H_ = 7.8, H^4^), 7.38 (d, ^3^*J*_H–H_ = 7.7, ^3^*J*_Pt–H_ = 68.1, 2H^*o*^), 7.23 (t, ^3^*J*_H–H_ = 6.4, H^3^), 6.82 (d, ^3^*J*_H–H_ = 7.4, 2H^*m*^), 6.68 (dd, ^4^*J*_H–H_ = 2.3, ^3^*J*_H–F_ = 9.2, ^3^*J*_Pt–H_ = 62.1, H^9^), 6.47 (ddd, ^3^*J*_H–F_ = 8.8, ^4^*J*_H–H_ = 2.3, H^7^), 2.25 (s, 3H, Me); ^13^C{^1^H} NMR [100.6 MHz, MeOD, δ]: 172.2 (m, ^2^*J*_Pt–C_ = 924, C^10^), 165.1 (dd, ^1^*J*_C–F_ = 254, ^3^*J*_Pt–C_ = 11.7, C^6^), 164.2 (d, ^2^*J*_Pt–C_ = 69, ^2^*J*_C–F_ = 7.3, C^11^), 163.7 (s, C^12^), 162.3 (dd, ^1^*J*_C–F_ = 258, ^3^*J*_Pt–C_ = 11.3, C^8^), 155.1 (m, CN^−^), 152.8 (s, ^2^*J*_Pt–C_ = 29.1, C^2^), 140.2 (s, ^1^*J*_Pt–C_ = 1002, C^ipso^), 139.6 (s, ^2^*J*_Pt–C_ = 42.2, C^*m*^), 139.3 (s, C^4^), 132.1 (m), 130.9 (s,^1^*J*_Pt–C_ = 11.7, C^*p*^), 128.6 (s, ^3^*J*_Pt–C_ = 76.1, C^*o*^), 123.9 (s, ^3^*J*_Pt–C_ = 12, C^3^), 123.5 (d, ^4^*J*_C–F_ = 21.6, ^3^*J*_Pt–C_ = 35.8, C^5^), 119.5 (dd, ^2^*J*_C–F_ = 16.4, ^4^*J*_C–F_ = 2.5, ^2^*J*_Pt–C_ = 62.2, C^9^), 99.2 (t, ^2^*J*_C–F_ = 27.6, C^7^), 21.0 (s, Me); ^19^F{^1^H} NMR [376.5 Mz, MeOD, δ]: −111.7 (d, ^4^*J*_F–Pt_ = 64, F^8^), −113.2 (d, ^4^*J*_F–Pt_ = 52, F^6^); ^195^Pt NMR [85.6 MHz, MeOD, δ]: −3711 (m).

### Synthesis of (NBu_4_)[Pt(bzq)(*p*-MeC_6_H_4_)(CN)] (**4**)

#### Method a

(NBu_4_)CN (61 mg, 0.227 mmol) was added to a suspension of [Pt(bzq)(*p*-MeC_6_H_4_)(SMe_2_)] (120 mg, 0.227 mmol) in acetone (25 mL). After 4 h of stirring at room temperature, a yellow solution was obtained. The solvent was evaporated to dryness, and the resulting solid was treated with Et_2_O (30 mL) to give **4** as a yellow solid (total yield: 163 mg, 98%).

#### Method b

NaCN (6.8 mg, 0.138 mmol) and [Pt(bzq)(*p*-MeC_6_H_4_)(SMe_2_)] (70 mg, 0.132 mmol) were dissolved in DMSO (5 mL) after 3 h stirring at room temperature; (NBu_4_)ClO_4_ (45.4 mg, 0.132 mmol) was added to yellow solution and stirred for 2 h. To reaction mixture was added H_2_O (100 mL) and extracted into CH_2_Cl_2_ (3 × 20 mL). Then CH_2_Cl_2_ solution was washed with H_2_O (3 × 25 mL). The organic layer was dried with MgSO_4_ and filtered through celite, and the filtrate was evaporated to dryness to give **4** as a yellow solid. (total yield: 59 mg, 61%). Elem. Anal. Calcd for C_37_H_51_N_3_Pt (732.92): C, 60.64; H, 7.01; N, 5.73. Found: C, 61.03; H, 7.08; N, 5.57; IR (ν(CN), cm^–1^): 2097; ESI-MS(−): *m/z* (%): 490.08 [Pt(bzq)(*p*-MeC_6_H_4_)(CN)]^−^ (100), 1222.44 [{Pt(bzq)(*p*-MeC_6_H_4_)(CN)}_2_(NBu_4_)]^−^ (10.7); ^1^H NMR [400 MHz, MeOD, δ]: 9.58 (d, ^3^*J*_H–H_ = 5, ^3^*J*_Pt–H_ = 19, H^2^), 8.44 (d, ^3^*J*_H–H_ = 8, H^4^), 7.76 (d, ^3^*J*_H–H_ = 8.5, H^5^), 7.56 (d, ^3^*J*_H–H_ = 8, ^3^*J*_Pt–H_ = 68.4, 2H^*o*^) 7.66–7.46 (m, 4H, H^3,6,7,9^), 7.41 (t, ^3^*J*_H–H_ = 5, H^8^) 6.81 (d, ^3^*J*_H–H_ = 8, 2H^*m*^), 2.96 (m, 8H, N–CH_2_ (NBu_4_))_,_ 2.26 (s, 3H, Me (*p*-MeC_6_H_4_)), 1.39 (q, 8H, ^3^*J*_H–H_ = 8, N–CH_2_–CH_2_– (NBu_4_)), 1.17 (sx, 8H, ^3^*J*_H–H_ = 8, −CH_2_–CH_3_ (NBu_4_)), 0.85 (t, 12H, ^3^*J*_H–H_ = 8, −CH_3_ (NBu_4_)); ^13^C{^1^H} NMR [100.6 MHz, MeOD, δ]: 164.0 (s, ^1^*J*_Pt–C_ = 931.5, C^10^), 157.3 (s, ^2^*J*_Pt–C_ = 63.8, C^11^), 156.5 (s, CN^−^), 151.3 (s, ^2^*J*_Pt–C_ = 28.06, C^2^), 146.1 (s, ^2^*J*_Pt–C_ = 11.3, C^13/14^), 140.4 (s, ^2^*J*_Pt–C_ = 45.6, C^*o*^), 139.1 (s, ^1^*J*_Pt–C_ = 1021, C^ipso^), 137.9 (s, C^4^), 135.9 (s, ^2^*J*_Pt–C_ = 98.0, C^9^), 134.8 (s, ^2^*J*_Pt–C_ = 29.4, C^12^), 130.7 (s, C^5^), 130.6 (s), 129.9 (s, ^3^*J*_Pt–C_ = 61.9, C^8^), 128.8 (s, C^*p*^), 128.5 (s, ^3^*J*_Pt–C_ = 77.9, C^*m*^), 128.1 (s, C^13/14^), 123.9 (s, C^6/7^), 123.1 (s, ^3^*J*_Pt–C_ = 13.5, C^3^), 122.5 (s, C^6/7^), 21.1 (s, Me), 59.3 (m, N–CH_2_ (NBu_4_)), 24.7 (s, N–CH_2_–CH_2_– (NBu_4_)), 21.2 (s, Me), 20.5 (s, −CH_2_–CH_3_ (NBu_4_)), 13.9 (s, −CH_3_ (NBu_4_)); ^195^Pt NMR [85.6 MHz, MeOD, δ]: −3760 (m).

### Synthesis of (NBu_4_)[Pt(ppy)(*p*-MeC_6_H_4_)(CN)] (**5**)

Complex **5** was obtained as a yellow solid (total yield: 210 mg, 88%) following the same procedure as **4** (Method a) starting from [Pt(ppy)(*p*-MeC_6_H_4_)(SMe_2_)] (156 mg, 0.310 mmol) and (NBu_4_)CN (83 mg, 0.310 mmol). (Method b) [Pt(ppy)(*p*-MeC_6_H_4_)(SMe_2_)] (120 mg, 0.238 mmol), NaCN (12.2 mg, 0.25 mmol), NBu_4_ClO_4_ (81.6 mg, 0.238 mmol), (total yield: 110 mg, 65%). Elem. Anal. Calcd for C_35_H_51_N_3_Pt (708.90): C, 59.30; H, 7.25; N, 5.93. Found: C, 58.98; H, 7.31; N, 5.92; IR (ν(CN), cm^–1^): 2094; ESI-MS(−): *m/z* (%): 466 [Pt(ppy)(*p*-MeC_6_H_4_)(CN)]^−^ (48); ^1^H NMR [400 MHz, MeOD, δ]: 9.33 (d, ^3^*J*_H–H_ = 5.3, ^3^*J*_Pt–H_ = 26.55 Hz, H^2^), 7.91 (m, H^4^, H^5^), 7.64 (m, H^7^), 7.42 (d, ^3^*J*_H–H_ = 7.4, ^3^*J*_Pt–H_ = 69.86, 2H^*o*^), 7.21 (m, H^3^, H^9^), 6.98 (m, H^6^, H^8^), 6.76 (d, ^3^*J*_H–H_ = 7, 2H^*m*^), 3.13 (m, 8H, N–CH_2_(NBu_4_)), 2.21 (s, 3H, Me (*p*-MeC_6_H_4_)), 1.55 (q, 8H, ^3^*J*_H–H_ = 7.4, N–CH_2_–CH_2_– (NBu_4_)), 1.32 (sx, 8H, ^3^*J*_H–H_ = 7.6, −CH_2_–CH_3_ (NBu_4_)), 0.95 (t, 12H, ^3^*J*_H–H_ = 7.3, −CH_3_ (NBu_4_)); ^13^C{^1^H} NMR [100.6 MHz, MeOD, δ]: 168.0 (s, C^12^), 166.1 (s, ^1^*J*_Pt–C_ = 920.4, C^10^), 157.0 (s, CN^−^), 152.7 (s, ^2^*J*_Pt–C_ = 28.3, C^2^), 149.7 (s, C^11^), 140.7 (s, ^2^*J*_Pt–C_ = 43.6, C^*o*^), 138.9 (s, C^4^/C^5^), 138.4 (s, C^9^), 130.4 (s, C^*p*^), 130.1 (s, C^8^), 128.3 (s, ^3^*J*_Pt–C_ = 78.5, C^*m*^), 124.3 (s, C^7^), 123.9 (d, C^3^, C^6–7^), 119.8 (s, C^4^/C^5^), 59.3 (m, N–CH_2_(NBu_4_)), 24.8 (s, N–CH_2_–CH_2_– (NBu_4_)), 21.2 (s, Me), 20.6 (s, −CH_2_–CH_3_ (NBu_4_)), 13.9 (s, −CH_3_ (NBu_4_)); ^195^Pt NMR [85.6 MHz, MeOD, δ]: −3731 (m).

### Synthesis of (NBu_4_)[Pt(dfppy)(*p*-MeC_6_H_4_)(CN)] (**6**)

Complex **6** was obtained as a yellow solid (total yield: 154 mg, 93%) following the same procedure as **4** (Method a) starting from [Pt(dfppy)(*p*-MeC_6_H_4_)(SMe_2_)] (120 mg, 0.223 mmol) and (NBu_4_)CN (59.8 mg, 0.223 mmol). Elem. Anal. Calcd for C_35_H_49_F_2_N_3_Pt (744.86): C, 56.43; H, 6.64; N, 5.64. Found: C,56.51; H, 6.64; N, 5.39; IR (ν(CN), cm^–1^): 2103; ESI-MS(−): *m/z* (%): 502 [Pt(dfppy)(*p*-MeC_6_H_4_)(CN)]^−^ (100), 411 [Pt(dfppy)(CN)] (5.1); ^1^H NMR [400 MHz, MeOD, δ]: 9.41 (d, ^3^*J*_H–H_ = 5.2, ^3^*J*_Pt–H_ = 24.9, H^2^), 8.16 (d, ^3^*J*_H–H_ = 8.2, H^5^), 7.99(t, ^3^*J*_H–H_ = 7.6, H^4^), 7.37 (d, ^3^*J*_H–H_ = 7.4, ^3^*J*_Pt–H_ = 67.1, 2H^*o*^), 7.26 (t, ^3^*J*_H–H_ = 6.3, H^3^), 6.80 d, ^3^*J*_H–H_ = 7.1, 2H^*m*^), 6.74 (dd, ^4^*J*_H–H_ = 2.2, ^3^*J*_H–F_ = 9.2, ^3^*J*_Pt–H_ = 61.4, H^9^), 6.48 (ddd, ^3^*J*_H–F_ = 8.8, ^4^*J*_H–H_ = 2, H^7^), 3.11 (m, 8H, N–CH_2_(NBu_4_)), 2.24 (s, 3H, Me (*p*-MeC_6_H_4_)), 1.54 (q, 8H, ^3^*J*_H–H_ = 7.5, N–CH_2_–CH_2_– (NBu_4_)), 1.32 (sx, 8H, ^3^*J*_H–H_ = 7.3, −CH_2_–CH_3_ (NBu_4_)), 0.94 (t, 12H, ^3^*J*_H–H_ = 7.2, −CH_3_ (NBu_4_)); ^13^C{^1^H} NMR [100.6 MHz, MeOD, δ]: 172.7 (m, ^2^*J*_Pt–C_ = 929, C^10^), 165.0 (dd, ^1^*J*_C–F_ = 254, ^3^*J*_Pt–C_ = 11, C^6^), 164.3 (d, ^3^*J*_C–F_ = 7, C^12^), 163.7 (dd, ^1^*J*_C–F_ = 258, ^3^*J*_Pt–C_ = 11.5, C^8^), 161.1 (d, 11.4, C^11^), 154.9 (m, CN^−^), 152.9 (s, ^2^*J*_Pt–C_ = 29.3, C^2^), 140.2 (s, C^ipso^), 140.1 (s, ^2^*J*_Pt–C_ = 41.4, C^*m*^), 139.3 (s, C^4^), 132.2 (m), 130.8 (s,^1^*J*_Pt–C_ = 10.9, C^*p*^), 128.5 (s, ^3^*J*_Pt–C_ = 75, C^*o*^), 124.0 (s, ^3^*J*_Pt–C_ = 13.8, C^3^), 123.5 (d, ^4^*J*_C–F_ = 21.6, ^3^*J*_Pt–C_ = 42.3, C^5^), 119.6 (dd, ^2^*J*_C–F_ = 16.5, ^4^*J*_C–F_ = 2.7, ^2^*J*_Pt–C_ = 69.3, C^9^), 99.2 (t, ^2^*J*_C–F_ = 27.5, C^7^), 59.4 (m, N–CH_2_(NBu_4_)), 24.8 (s, N–CH_2_–CH_2_– (NBu_4_)), 21.1 (s, Me (*p*-MeC_6_H_4_)), 20.6 (s, −CH_2_–CH_3_ (NBu_4_)), 13.9 (s, −CH_3_ (NBu_4_)); ^19^F{^1^H} NMR [376.5 MHz, MeOD, δ]: −111.6 (d, ^4^*J*_F–Pt_ = 64, F^8^), −113.1 (d, ^4^*J*_F–Pt_ = 51, F^6^); ^195^Pt NMR [85.6 MHz, MeOD, δ]: −3706 (m).

### Synthesis of (NBu_4_)[Pt_2_(bzq)_2_(*p*-MeC_6_H_4_)_2_(μ*-*CN)] (**7**)

Complex [Pt(bzq)(*p*-MeC_6_H_4_)(SMe_2_)] (87 mg, 0.165 mmol) was added to a solution of **4** (121 mg, 0.165 mmol) in acetone (25 mL). After 4 h of stirring at 45–50 °C, a yellow solution was obtained. The solvent was evaporated to dryness, and the resulting solid was treated with Et_2_O (15 mL) and cold CHCl_3_ (5 mL) to give **7** as a yellow solid (total yield: 176 mg, 89%). Elem. Anal. Calcd for C_57_H_66_N_4_Pt_2_ (1197.33): C, 57.17; H, 5.57; N, 4.68. Found: C, 57.49; H, 5.63; N, 4.54; IR (ν(CN), cm^–1^): 2127; MALDI-TOF (−): *m/z* (%): 954 [Pt_2_(bzq)_2_(*p*-MeC_6_H_4_)_2_(CN)]^−^ (100); ^1^H NMR [400 MHz, CD_2_Cl_2_, δ]: 9.33 (d, ^3^*J*_H–H_ = 4.5, ^3^*J*_Pt–H_ = 19.9, H^2^/H^2′^), 9.21 (d, ^3^*J*_H–H_ = 4.5, ^3^*J*_Pt–H_ = 19.9, H^2^/H^2′^), 8.30 (d, ^3^*J*_H–H_ = 8.2, H^4^/H^4′^), 8.27 (d, ^3^*J*_H–H_ = 8.2, H^4^/H^4′^), 7.76–7.70 (m, H^*o*^/H^*o*′^, H^5^/H^5^′), 7.56–7.33 (m, H^3^/ H^3′^, H^6–9^/ H^6′–9′^), 7.01 (d, ^3^*J*_H–H_ = 2.7, H^*m*^/H^*m*′^), 6.99 (d, ^3^*J*_H–H_ = 2.8, H^*m*^/H^*m*′^), 2.77 (m, 8H, N–CH_2_(NBu_4_)), 2.38 (d, 6H, Me (*p*-MeC_6_H_4_)), 1.15 (qu, 8H, ^3^*J*_H–H_ = 7.6, N–CH_2_–CH_2_– (NBu_4_)), 0.85 (sx, 8H, ^3^*J*_H–H_ = 7.3, −CH_2_–CH_3_ (NBu_4_)), 0.58 (t, 12H, ^3^*J*_H–H_ = 7.2, −CH_3_ (NBu_4_)). ^13^C{^1^H} NMR [100.6 MHz, CD_2_Cl_2_, δ]: 163.9 (s), 156.4 (s), 155.0 (s), 153.8 (s), 151.3 (s, C^2^/C^2^′), 148.5 (s, C^2^/C^2′^), 147.4 (s), 145.2 (s), 144.0 (s), 141.8 (s), 140.2 (d, C*^o^, C*^*o*′^), 139.5 (s), 136.4 (d, C^4^, C^4′^), 134.7 (d), 133.8 (s), 130.5 (s), 130.2 (s), 129.8 (s), 129.5 (s), 129.3 (d, C^5^,C^5^′), 128.1 (s), 127.7 (s, C^*m*^, C^*m*^′), 127.0 (s), 126.5 (s), 123.2 (d), 122.4 (s), 121.8 (s), 121.6 (s), 120.4 (s), 58.8 (s, N–CH_2_(NBu_4_)), 24.2 (s, N–CH_2_–CH_2_– (NBu_4_)), 21.3 (s, Me), 19.7 (s, −CH_2_–CH_3_ (NBu_4_)), 13.6 (s, −CH_3_ (NBu_4_)); ^195^Pt NMR [85.6 MHz, CD_2_Cl_2_, δ]: −3578 (m, Pt-NC), −3770 (m, Pt–CN).

### Synthesis of (NBu_4_)[Pt_2_(ppy)_2_(*p*-MeC_6_H_4_)_2_(μ*-*CN)] (**8**)

Complex **8** was obtained as a light green solid (total yield: 161 mg, 84%) following the same procedure as **7** starting from [Pt(ppy)(*p*-MeC_6_H_4_)(SMe_2_)] (83 mg, 0.166 mmol) and **5** (118 mg, 0.166 mmol). Elem. Anal. Calcd for C_53_H_66_N_4_Pt_2_ (1149.29): C, 55.38; H, 5.80; N, 4.88. Found: C, 55.90; H, 5.77; N, 4.69. IR (ν(CN), cm^–1^): 2123; MALDI-TOF (−): *m/z* (%): 906 [Pt_2_(ppy)_2_(*p*-MeC_6_H_4_)_2_(CN)]^−^ (100); ^1^H NMR [400 MHz, CDCl_3_, δ]: 9.11 (d, ^3^*J*_H–H_ = 4.8, ^3^*J*_Pt–H_ = 18.7, H^2^/H^2′^), 9.01 (d, ^3^*J*_H–H_ = 5.2, ^3^*J*_Pt–H_ = 16.8, H^2^/H^2′^), 7.75–6.91 (other aromatic region), 6.86 (d, ^3^*J*_H–H_ = 4.6, H^*m*^/ H^*m*′^), 6.84 (d, ^3^*J*_H–H_ = 4.6, H^*m*^/H^*m*′^), 2.72 (m, 8H, N–CH_2_(NBu_4_)), 2.27 (d, 6H, Me (*p*-MeC_6_H_4_)), 1.12 (qu, 8H, ^3^*J*_H–H_ = 7.05, N–CH_2_–CH_2_– (NBu_4_)), 0.81 (sx, 8H, ^3^*J*_H–H_ = 7.4, −CH_2_–CH_3_ (NBu_4_)), 0.57 (t, 12H, ^3^*J*_H–H_ = 7.2, −CH_3_ (NBu_4_)); ^13^C{^1^H} NMR [100.6 MHz, CDCl_3_, δ]: 166.6 (s), 165.2 (s), 164.6 (s), 154.2 (s, CN^−^), 152.5 (s, C^2^ / C^2^′), 149.3 (s, C^2^ /C^2^′), 148.7 (s), 148.2 (s), 146.7 (s), 143.0 (s, C^*p*^), 140.5 (s), 139.7 (d, C^*o*^, C^*o*^′), 137.5 (s), 137.2 (s), 137.1 (d), 129.7 (s), 129.3 (t, C^ipso^), 127.3 (d, C^*m*^, C^*m*^′), 122.9 (t), 122.6 (s), 121.9 (d), 117.9 (d), 58.3 (m, N–CH_2_(NBu_4_)), 31.05 (s, acetone), 24.0 (s, N–CH_2_–CH_2_– (NBu_4_)), 21.2 (s, Me), 19.4 (s, −CH_2_–CH_3_ (NBu_4_)), 13.6 (s, −CH_3_ (NBu_4_)); ^195^Pt NMR [85.6 MHz, CDCl_3_, δ]: −3558 (m, Pt-NC), −3759 (m, Pt–CN).

### Synthesis of (NBu_4_)[Pt_2_(dfppy)_2_(*p*-MeC_6_H_4_)_2_(μ*-*CN)] (**9**)

Complex **9** was obtained as a greenish yellow solid (total yield: 106 mg, 89%) following the same procedure as **7** starting from [Pt(dfppy)(*p*-MeC_6_H_4_)(SMe_2_)] (65 mg, 0.121 mmol) and **6** (90 mg, 0.121 mmol). Elem. Anal. Calcd for C_53_H_62_F_4_N_4_Pt_2_ (1221.25): C, 52.12; H, 5.13; N, 4.59. Found: C, 52.36; H, 5.13; N, 4.42. IR (ν(CN), cm^–1^): 2130; MALDI-TOF (−): *m/z* (%): 978 [Pt_2_(dfppy)_2_(*p*-MeC_6_H_4_)_2_(CN)]^−^ (100); ^1^H NMR [400 MHz, CD_2_Cl_2_, δ]: 8.89 (d, ^3^*J*_H–H_ = 5.2, ^3^*J*_Pt–H_ = 22.9, H^2^/H^2′^), 8.90 (d, ^3^*J*_H–H_ = 5.2, ^3^*J*_Pt–H_ = 21.7, H^2^/H^2′^), 8.09 (d, ^3^*J*_H–H_ = 8.4, H^5^/H^5′^), 8.05 (d, ^3^*J*_H–H_ = 8.4, H^5^/H^5′^), 7.84 (t, ^3^*J*_H–H_ = 8.2, H^4^/H^4′^), 7.82 (t, ^3^*J*_H–H_ = 8.2, H^4^/H^4′^), 7.50 (t, ^3^*J*_H–H_ = 8.0, ^3^*J*_Pt–H_ = 62.8, 2H^*o*^, 2H^*o′*^), 6.99 (m, ^3^*J*_H–H_ = 6.4, H^3^, H^3′^), 6.95 (d, ^3^*J*_H–H_ = 2.6, 2H^*m*^/H^*m*′^), 6.95 (d, ^3^*J*_H–H_ = 2.6, 2H^*m*^/H^*m*′^), 6.82 (dd, ^4^*J*_H–H_ = 2.2, ^3^*J*_H–F_ = 9.8, H^9^/H^9′^), 6.78 (dd, ^4^*J*_H–H_ = 2.2, ^3^*J*_H–F_ = 9.2, H^9^/ H^9′^), 6.45 (t, H^7^/H^7′^), 6.45 (t, H^7^/H^7′^), 2.88 (m, 8H, N–CH_2_(NBu_4_)), 2.33 (d, 6H, Me (*p*-MeC_6_H_4_)), 1.36 (qu, 8H, ^3^*J*_H–H_ = 7.6, N–CH_2_–CH_2_– (NBu_4_)), 1.06 (sx, 8H, ^3^*J*_H–H_ = 7.3, −CH_2_–CH_3_ (NBu_4_)), 0.74 (t, 12H, ^3^*J*_H–H_ = 7.1, −CH_3_ (NBu_4_)). ^13^C{^1^H} NMR [100.6 MHz, MeOD, δ]: 152.8 (m, C^2^, C^2′^), 149.4 (s), 142.9 (s, C^*p*^), 139.4 (d, C^*o*^, C^*o′*^), 138.1 (dd, C^4^, C^4′^), 130.7 (s), 130.4 (s, C^ipso^), 128.2 (s), 127.8 (s, C^*m*^, C^*m′*^), 123.2 (s), 122.6 (s, C^3^, C^3′^), 97.8 (s, C^7^, C^7′^), 59.1 (m, N–CH_2_(NBu_4_)), 24.3 (s, Me), 21.1 (d, N–CH_2_–CH_2_– (NBu_4_)), 19.9 (s, −CH_2_–CH_3_ (NBu_4_)), 13.6 (s, −CH_3_ (NBu_4_)). ^19^F{^1^H} NMR [376.5 MHz, CD_2_Cl_2_, δ]: −110.4 (d, ^4^*J*_F–Pt_ = 65.1, F^8^/F^8′^), −110.6 (d, ^4^*J*_F–Pt_ = 84.0, F^8^/F^8′^), −111.8 (d, ^4^*J*_F–Pt_ = 54.2, F^6^/F^6′^), −112.2 (d, ^4^*J*_F–Pt_ = 70.8, F^6^/F^6′^); ^195^Pt NMR [85.6 MHz, CD_2_Cl_2_, δ]: −3515 (m, Pt-NC), −3721 (m, Pt–CN).

### X-ray Diffraction

X-ray intensity data were collected using molybdenum graphite monochromatic (Mo-K_α_) radiation with a Bruker APEX-II diffractometer at a temperature of 100 K using APEX-II programs for all complexes. Structures were solved by Intrinsic Phasing using SHELXT^84^ with the WinGX graphical user interface.^85^ Multi-scan absorption corrections were applied to all the data sets and refined by full-matrix least squares on *F*^2^ with.^84^ Hydrogen atoms were positioned geometrically, with isotropic parameters *U*_iso_ = 1.2 *U*_eq_ (parent atom) for aromatic hydrogens and CH_2_ and *U*_iso_ = 1.5 *U*_eq_ (parent atom) for methyl groups. Finally, the structures show some residual peaks in the vicinity of the platinum atoms but with no chemical meaning.

### Computational Details

Calculations were performed with the program suite Gaussian 16^86^ on complexes with Becke’s three-parameter functional combined with Lee-Yang-Parr’s correlation functional.^87,88^ Optimizations on the singlet state (S_0_) were performed using as a starting point the molecular geometry obtained through X-ray diffraction analysis for complexes **5–9** or with the simulated one for **4** with no symmetry restriction. No imaginary frequency was found in the vibrational frequency analysis of the final equilibrium geometries. The LANL2DZ basis set was used for the platinum centers, and all-electron 6-31G (d, p) basis set was applied for other atoms.^89^ Solvent effects were taken into consideration by the polarizable continuum model^90^ implemented in the Gaussian 16 software, in the presence of methanol. The TD-DFT method was employed for calculations of the electronic absorption spectra. The predicted emission wavelengths were calculated by the energy difference between the triplet state at its optimized geometry and the singlet state at the triplet geometry. The results were visualized with GaussView 6. Overlap populations between molecular fragments were calculated using the GaussSum 3.0 program.^91^

## Supporting Information Available

Characterization of complexes (NMR spectra and crystal data), photophysical properties, and computational details (PDF)
