# Supplementary material for: Luminescent Anionic Cyclometalated Organoplatinum (II) Complexes with Terminal and Bridging Cyanide Ligand: Structural and Photophysical Properties
Source: Inorg Chem. 2023 Jan 18;62(4):1513–29. doi: 10.1021/acs.inorgchem.2c03668 (PMC9890487; doi:10.1021/acs.inorgchem.2c03668)
Supplement: Supplementary file 1 — ic2c03668_si_001.pdf [file ic2c03668_si_001.pdf]

Supporting Information

Luminescent Anionic Cyclometalated Organoplatinum (II) Complexes with Terminal and Bridging Cyanide Ligand: Structural and Photophysical Properties

Mina Sadeghian,[a, b] David Gómez de Segura,[b] Mohsen Golbon Haghighi,*[,a] Nasser Safari,[a] Elena Lalinde*[,b] and M. Teresa Moreno*[,b]

[a] Department of Chemistry, Shahid Beheshti University, Evin, Tehran 19839-69411, Iran. E-mail: m_golbon@sbu.ac.ir

[b] Departamento de Química-Centro de Síntesis Química de La Rioja (CISQ), Universidad de La Rioja, 26006 Logroño, Spain. E-mail: elena.lalinde@unirioja.es; teresa.moreno@unirioja.es

Contents: Page

1.- NMR Spectra.. S2
2.- Mass Spectra.. S19
3.- Crystal Structures.. S22
4.- Photophysical Properties and Theoretical calculations........................... S25

1.- NMR Spectra

a)

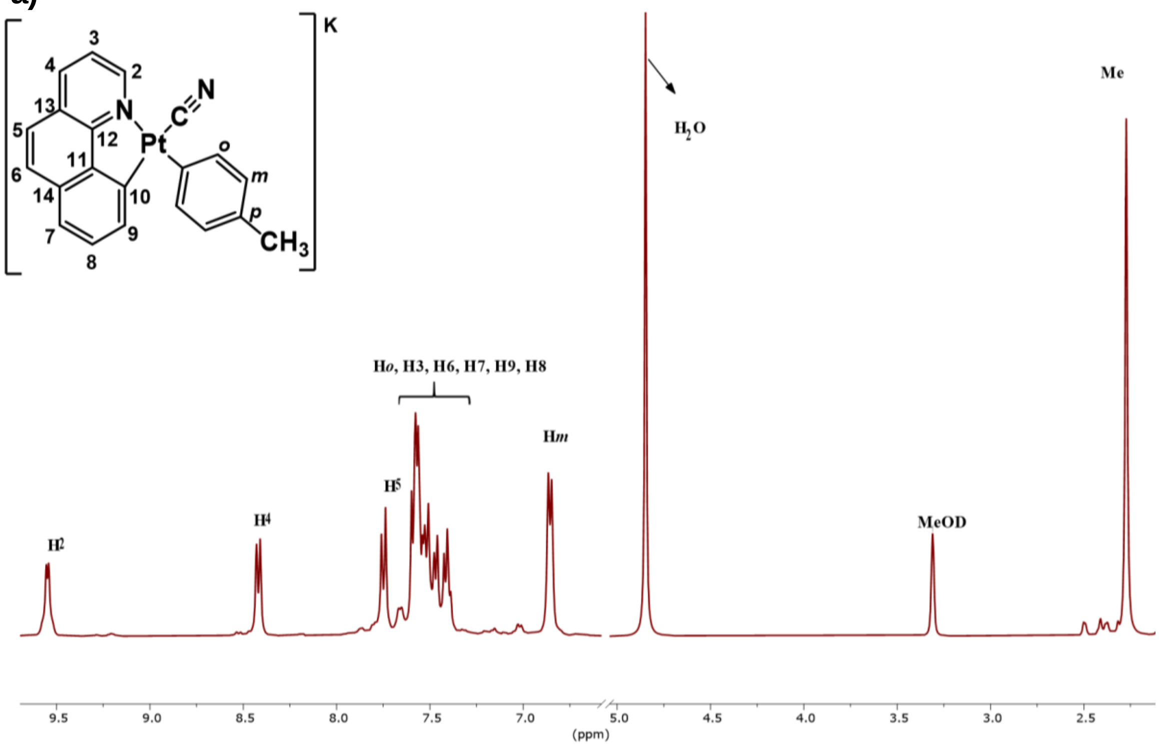

b)

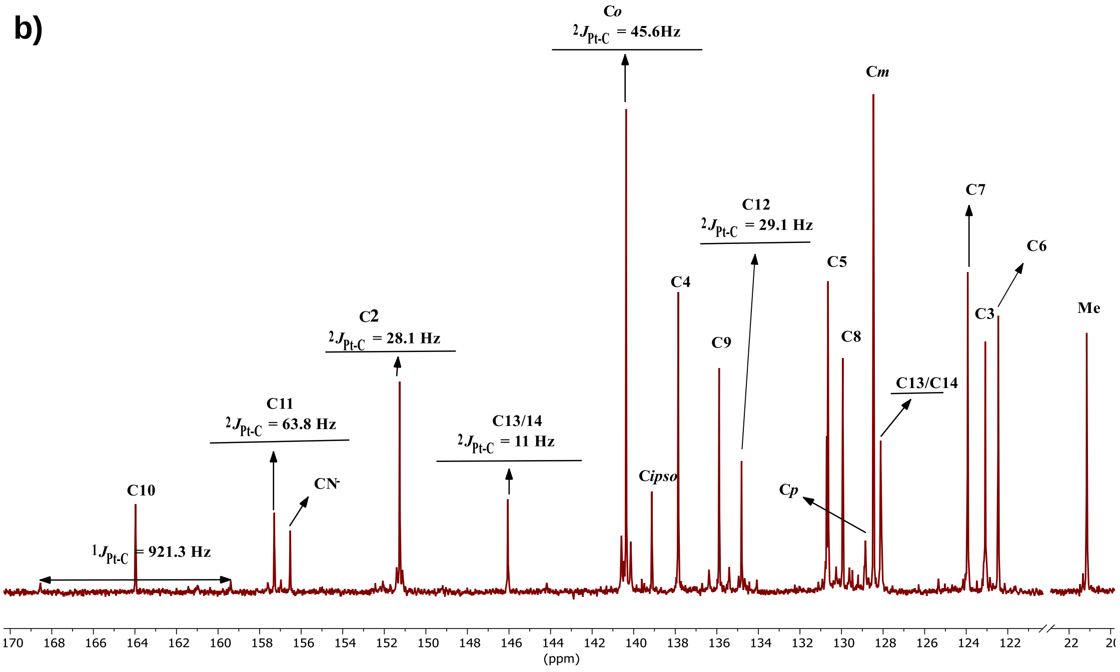

c)

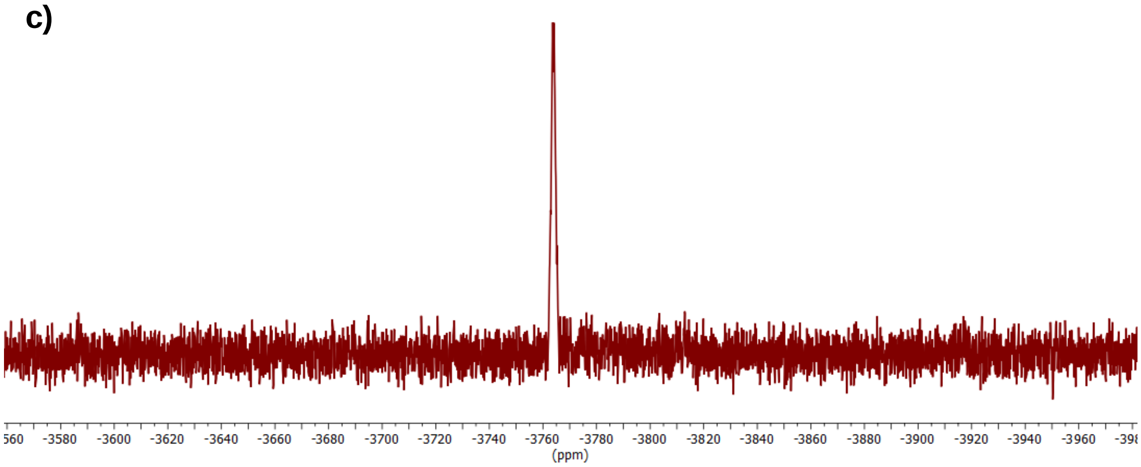

Figure S1. NMR spectra of K[Pt(bzq)(p-MeC$_6$H$_4$)(CN)] (**1**) in MeOD (a) ^{1}H, (b) ^{13}C{^{1}H} and (c) ^{195}Pt.

a)

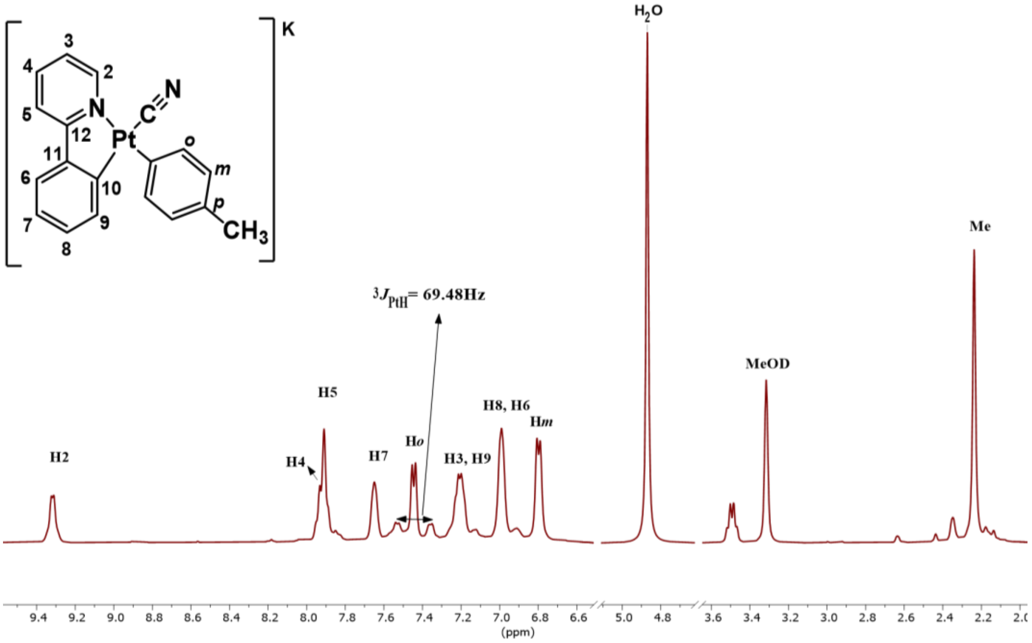

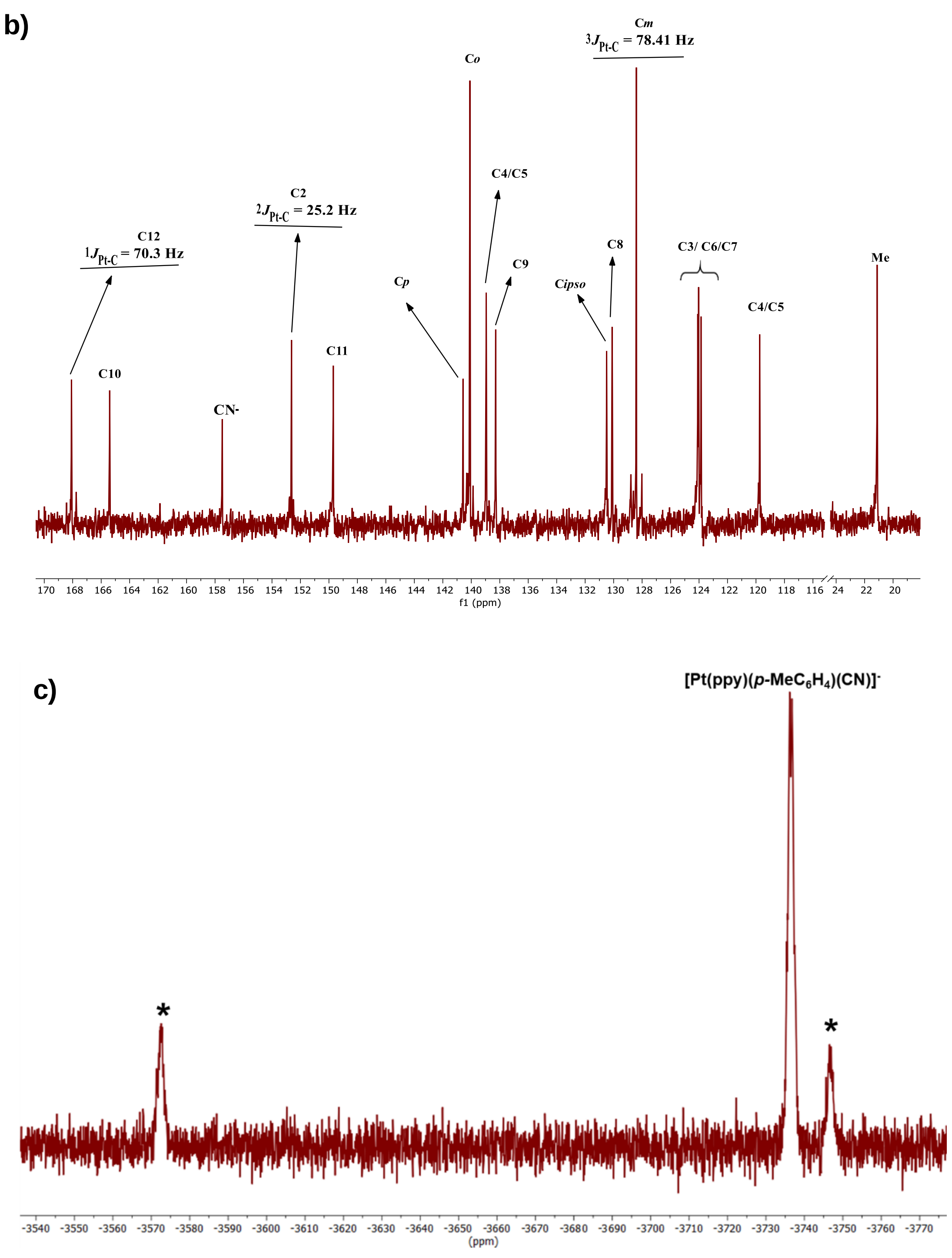

Figure S2. NMR spectra of K[Pt(ppy)(p-MeC$_6$H$_4$)(CN)] (**2**) in MeOD (a) ^{1}H, (b) ^{13}C{^{1}H} and (c) ^{195}Pt (* signals assigned to [Pt$_2$(ppy)$_2$(p-MeC$_6$H$_4$)$_2$(μ-CN)] (**8**))

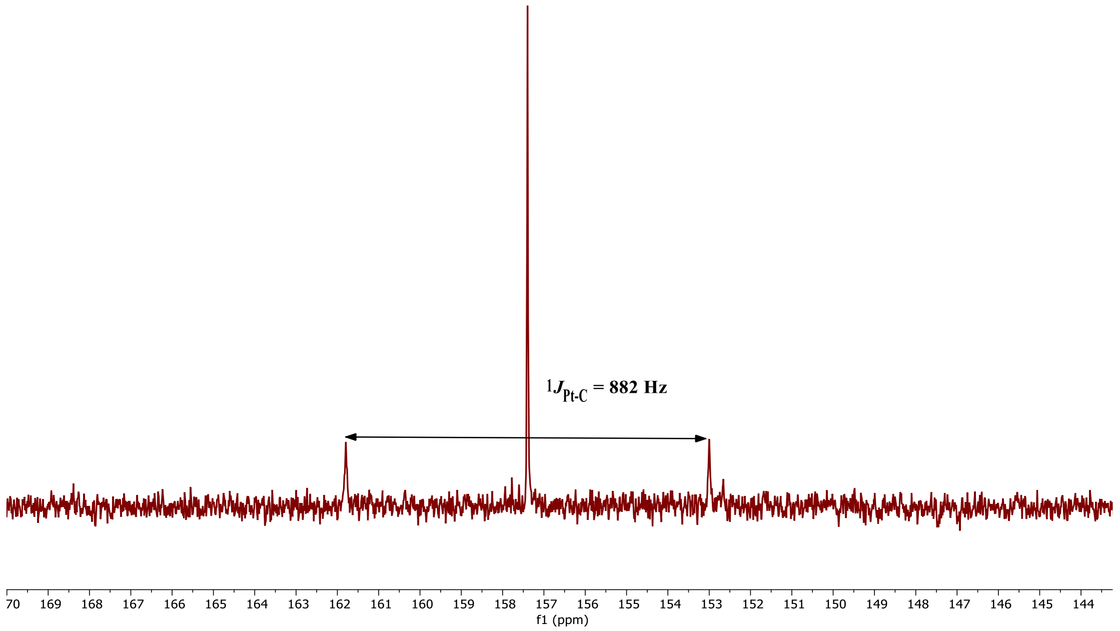

Figure S3. Selected region of the $^{13}C\{^1H\}$ NMR spectrum of K[Pt(ppy)(*p*-MeC_6H_4)(^{13}CN)] (**2'**) in MeOD.

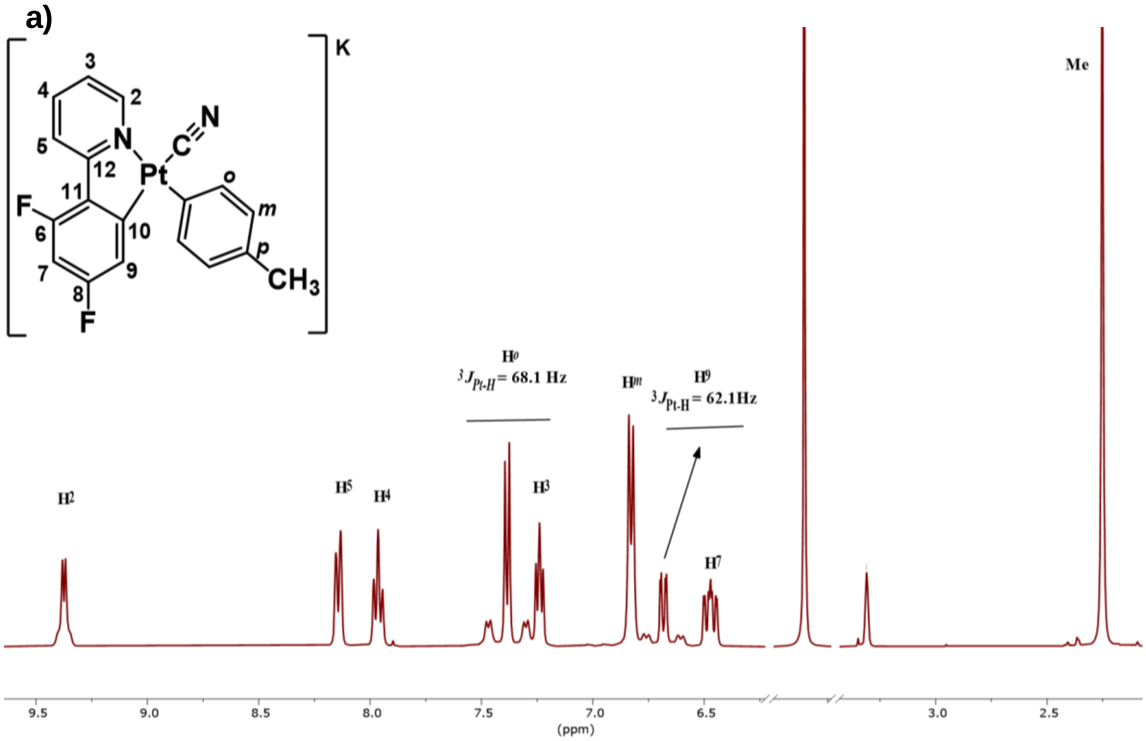

b)

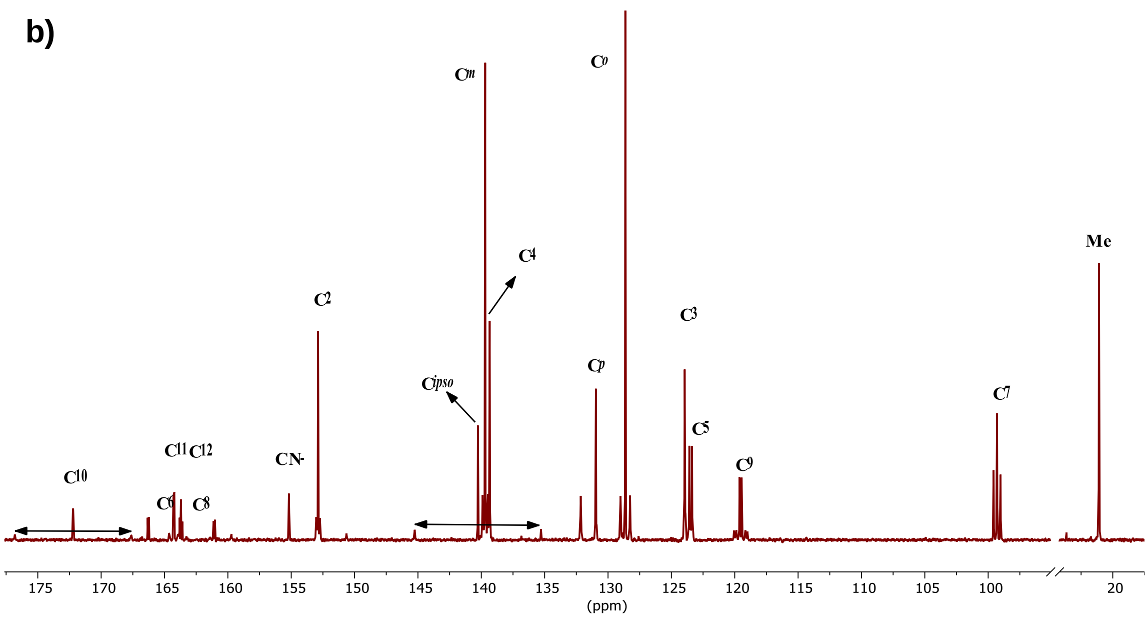

Cm
Co
C4
Me
C2
C3
Cipso
Cp
C7
C5
C11 C12
CN-
C9
C10
C6
C8
175 170 165 160 155 150 145 140 135 130 125 120 115 110 105 100 20
(ppm)

c)

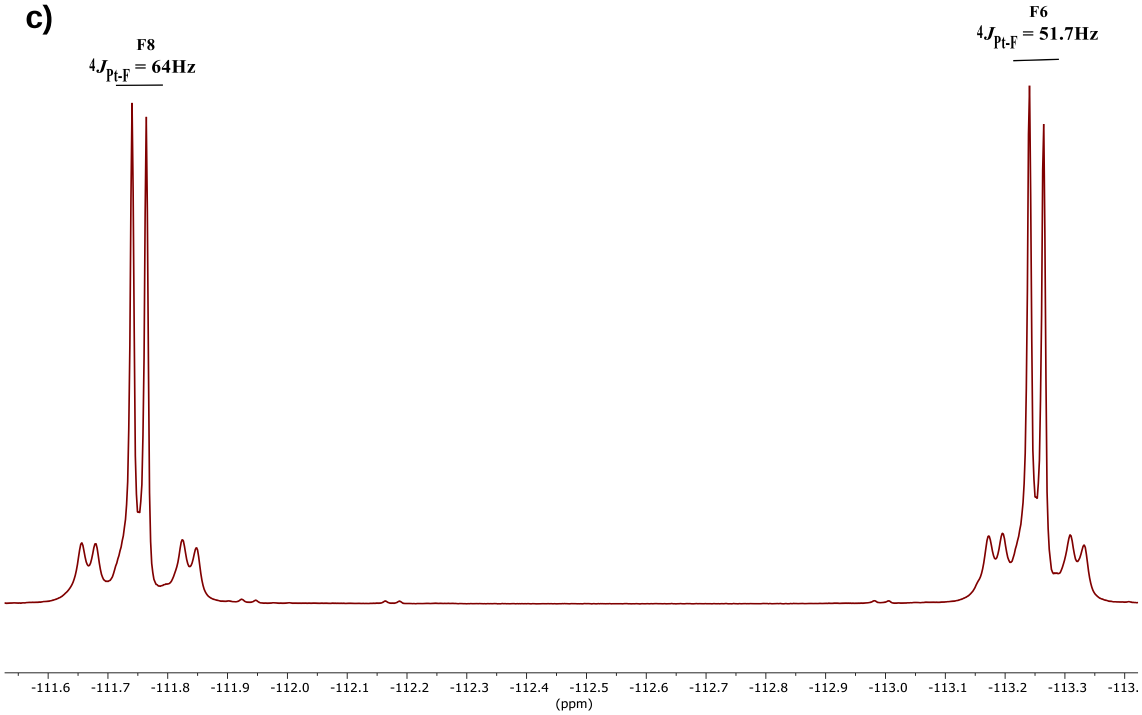

F8
$^4J_{Pt-F}$ = 64Hz
F6
$^4J_{Pt-F}$ = 51.7Hz
-111.6 -111.7 -111.8 -111.9 -112.0 -112.1 -112.2 -112.3 -112.4 -112.5 -112.6 -112.7 -112.8 -112.9 -113.0 -113.1 -113.2 -113.3 -113.
(ppm)

d)

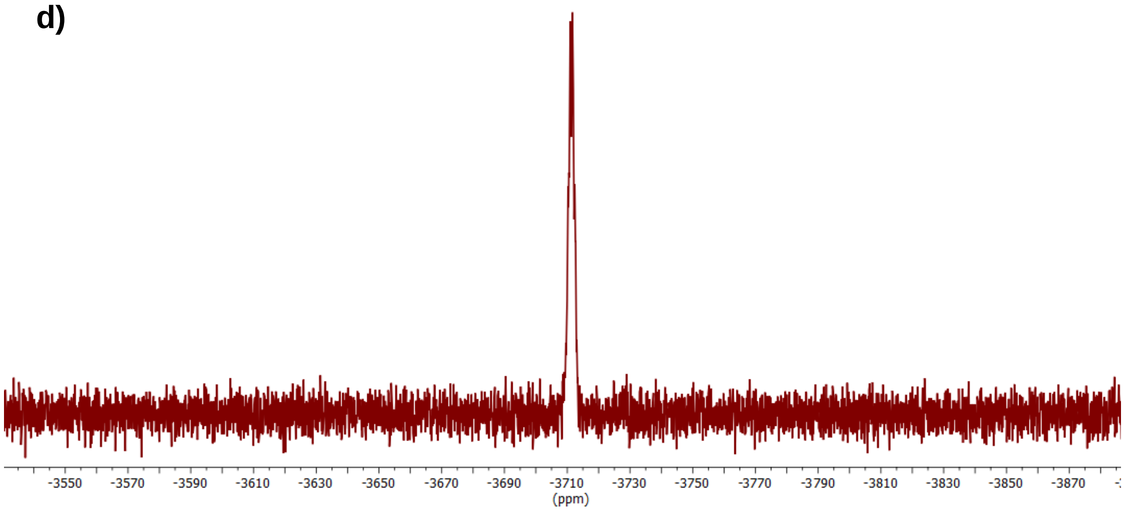

Figure S4. NMR spectra of K[Pt(dfppy)(*p*-MeC$_6$H$_4$)(CN)] (**3**) in MeOD (a) ^{1}H, (b) ^{13}C{^{1}H}, (c) ^{19}F{^{1}H} and (d) ^{195}Pt.

a)

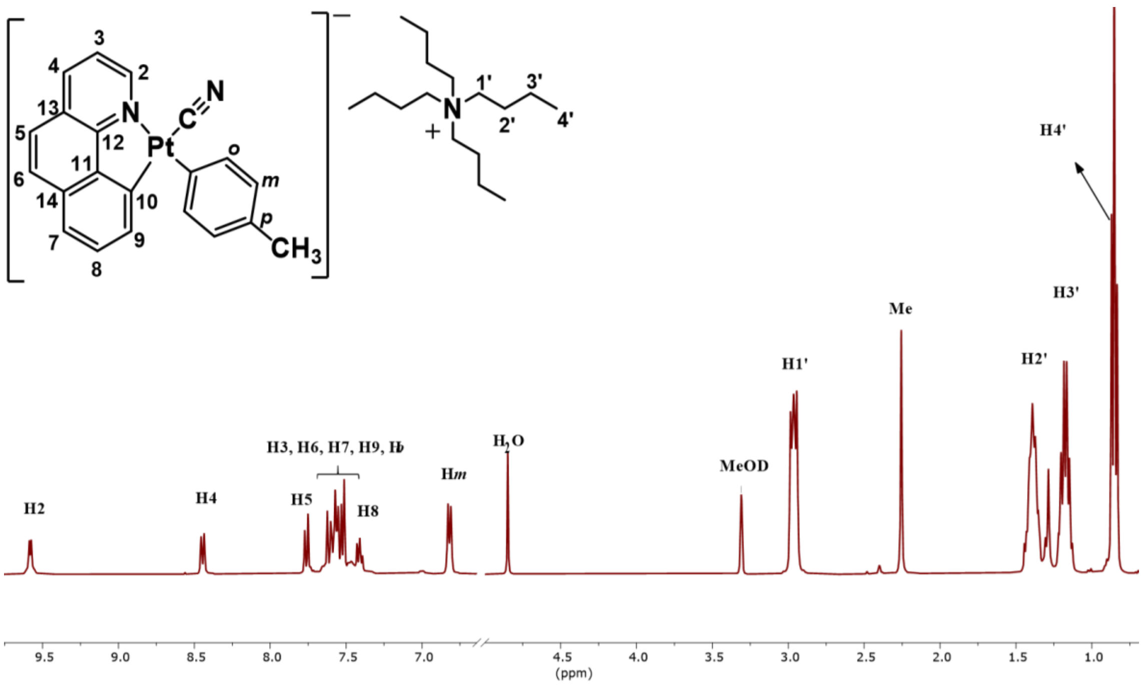

b)

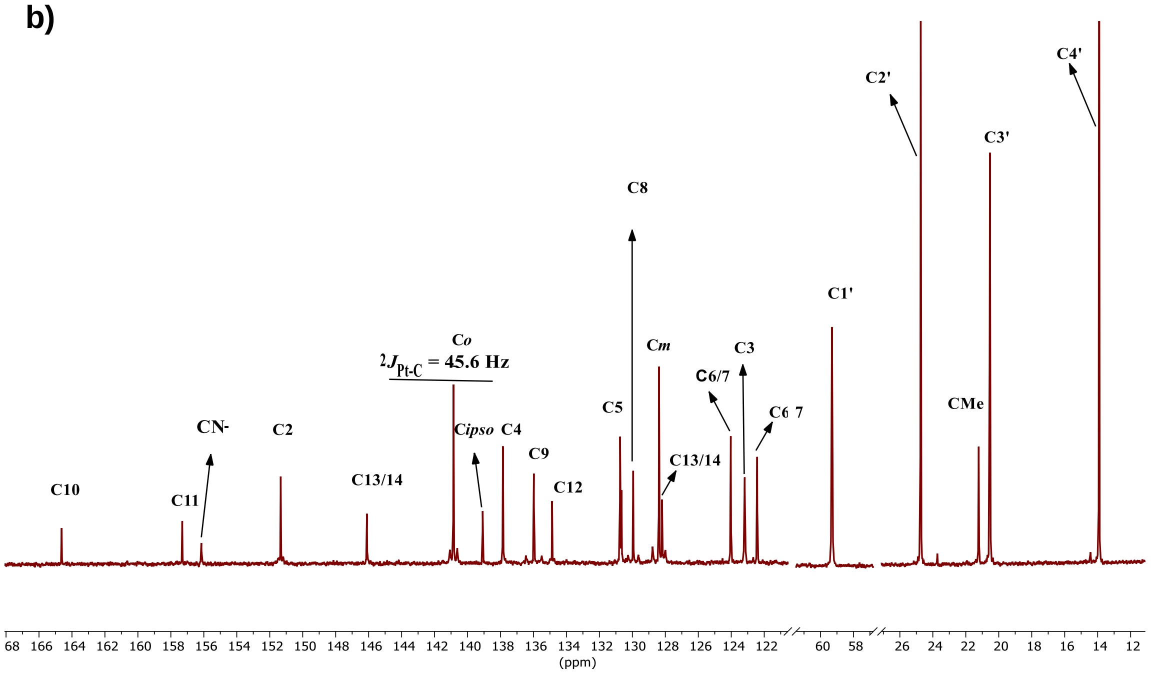

c)

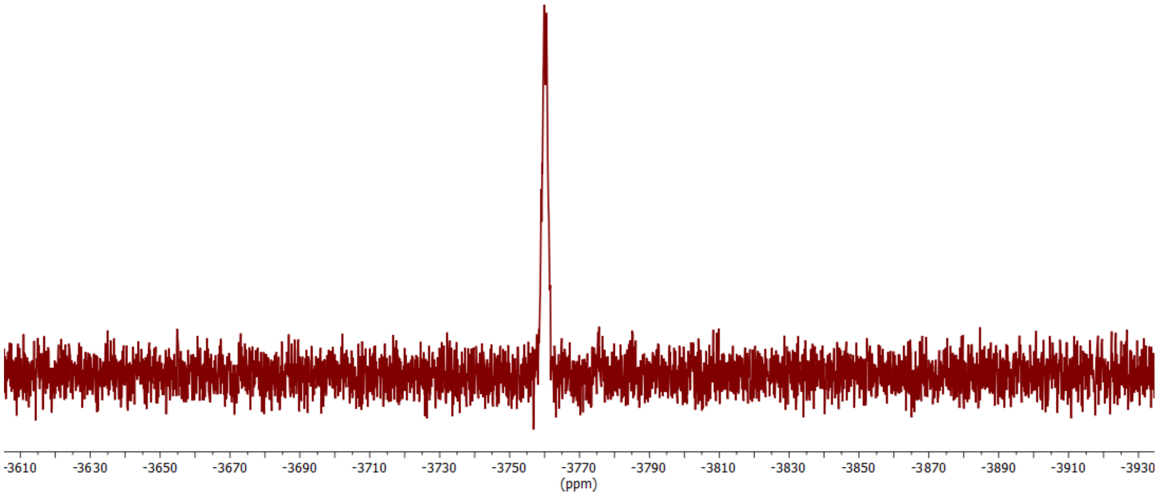

Figure S5. NMR spectra of $NBu_4[Pt(bzq)(p\text{-}MeC_6H_4)(CN)]$ (**4**) in MeOD (a)^{1}H, (b) $^{13}C\{^1H\}$ and (c) ^{195}Pt.

a)

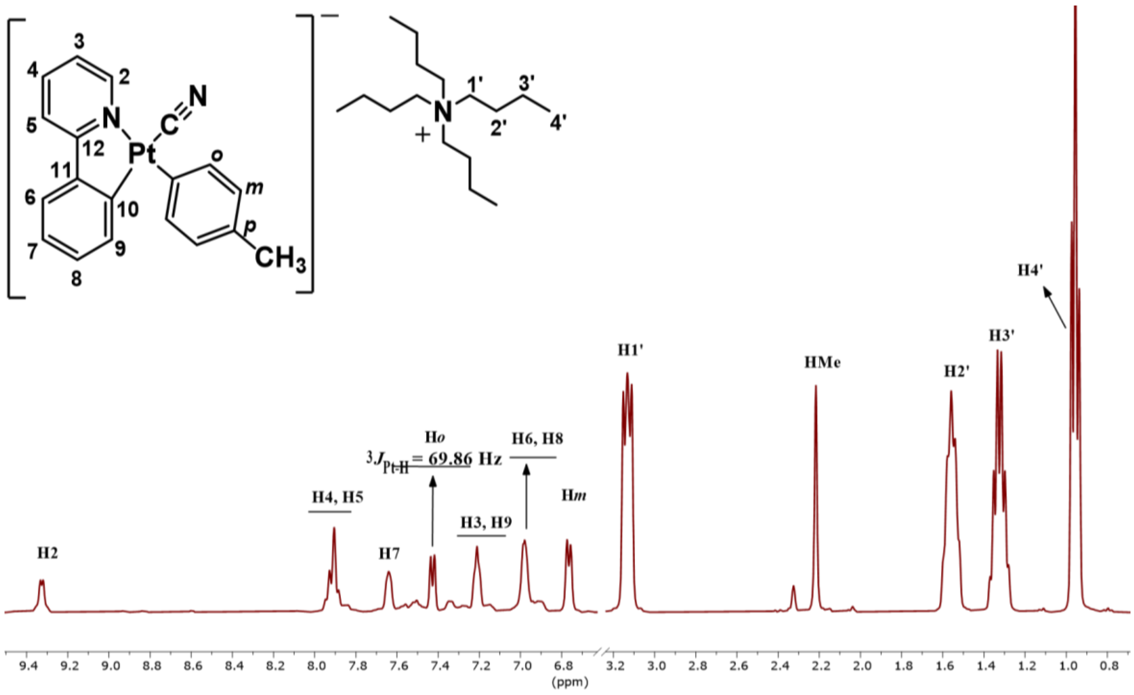
H4'
H3'
H1'
HMe
H2'
Ho
$^{3}J_{Pt-H}$ = 69.86 Hz
H6, H8
H4, H5
H3, H9
Hm
H2
H7
(ppm)

b)

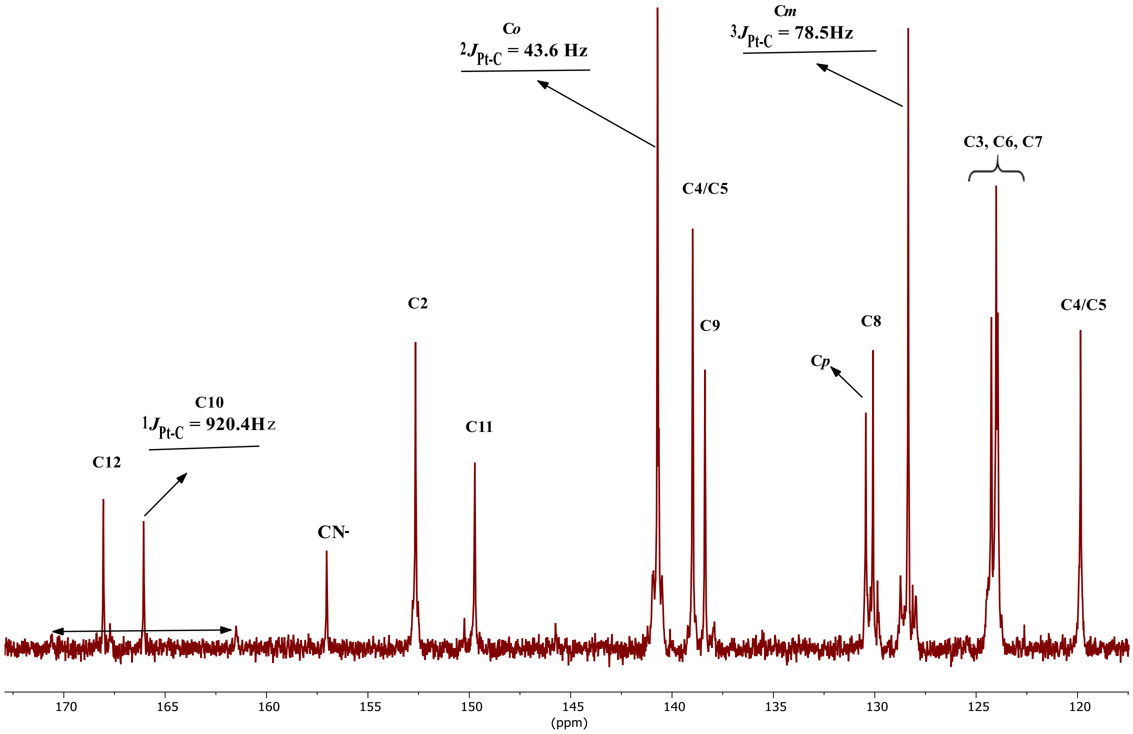
Co
$^{2}J_{Pt-C}$ = 43.6 Hz
Cm
$^{3}J_{Pt-C}$ = 78.5Hz
C3, C6, C7
C4/C5
C2
C9
C8
C4/C5
Cp
C10
$^{1}J_{Pt-C}$ = 920.4Hz
C11
C12
CN-
(ppm)

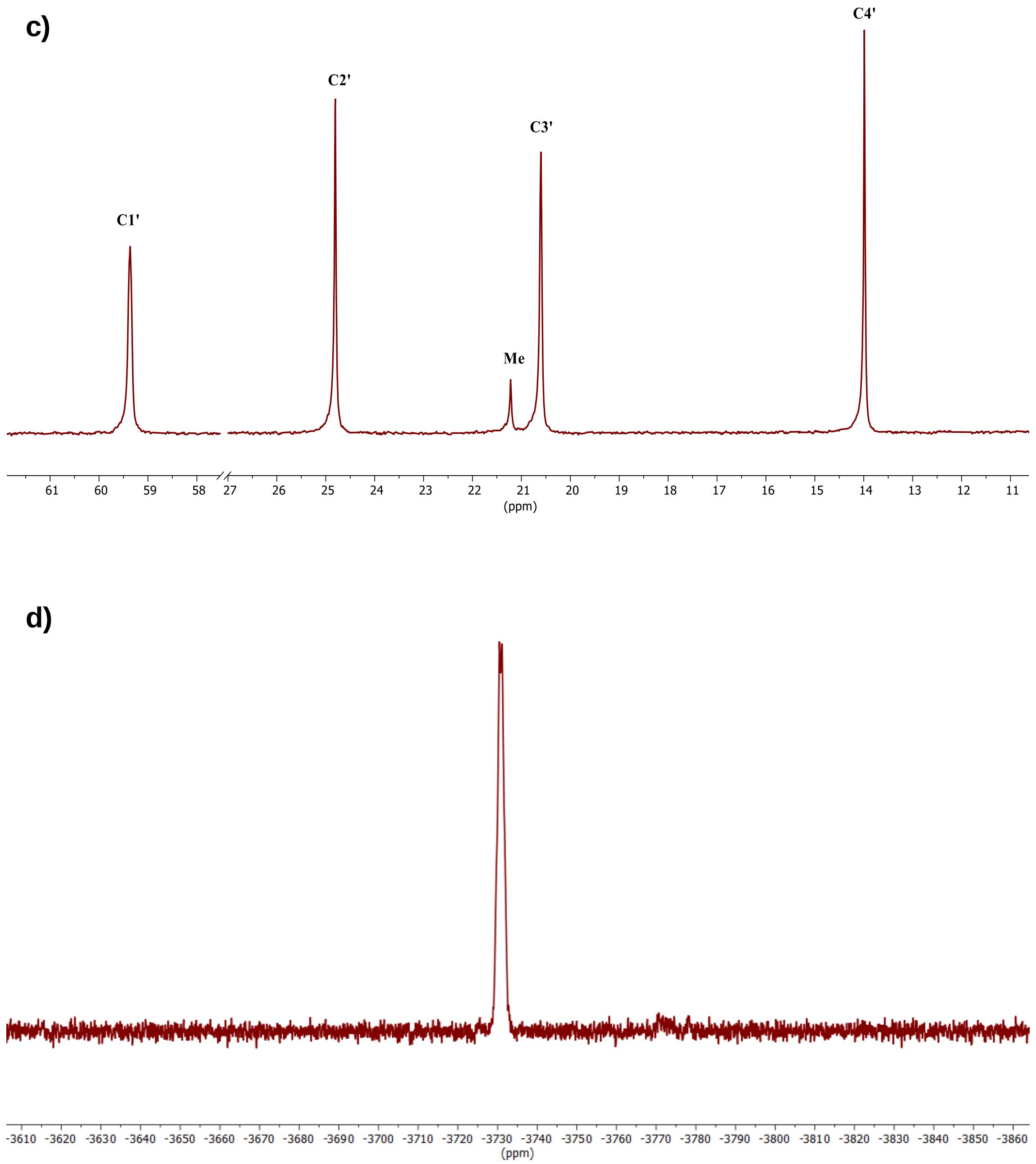

Figure S6. NMR spectra of $NBu_4[Pt(ppy)(p\text{-}MeC_6H_4)(CN)]$ (**5**) in MeOD (a) ^{1}H and (b) aromatic and (c) aliphatic region of the $^{13}C\{^{1}H\}$ and (d) ^{195}Pt.

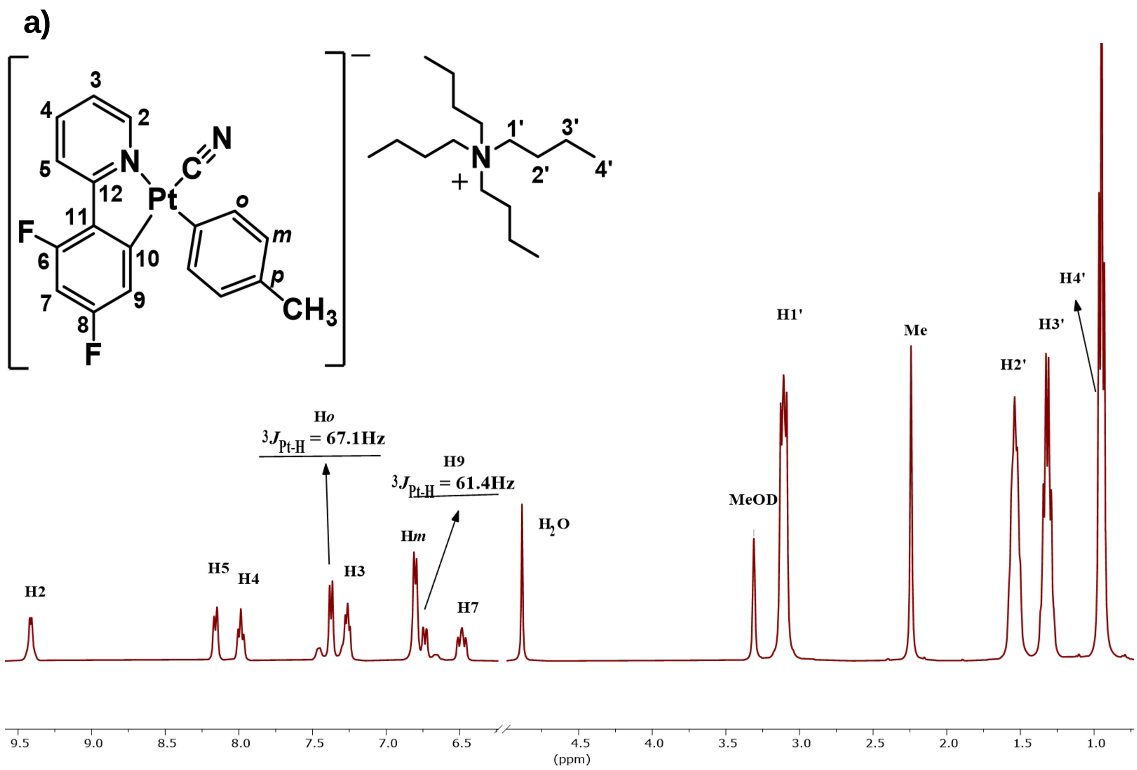
a)
F
N
Pt
C≡N
CH3
F
N
+
1'
2'
3'
4'
H2
H5
H4
Ho
3JPt-H = 67.1Hz
H3
Hm
H9
3JPt-H = 61.4Hz
H7
H2O
MeOD
H1'
Me
H2'
H3'
H4'
(ppm)

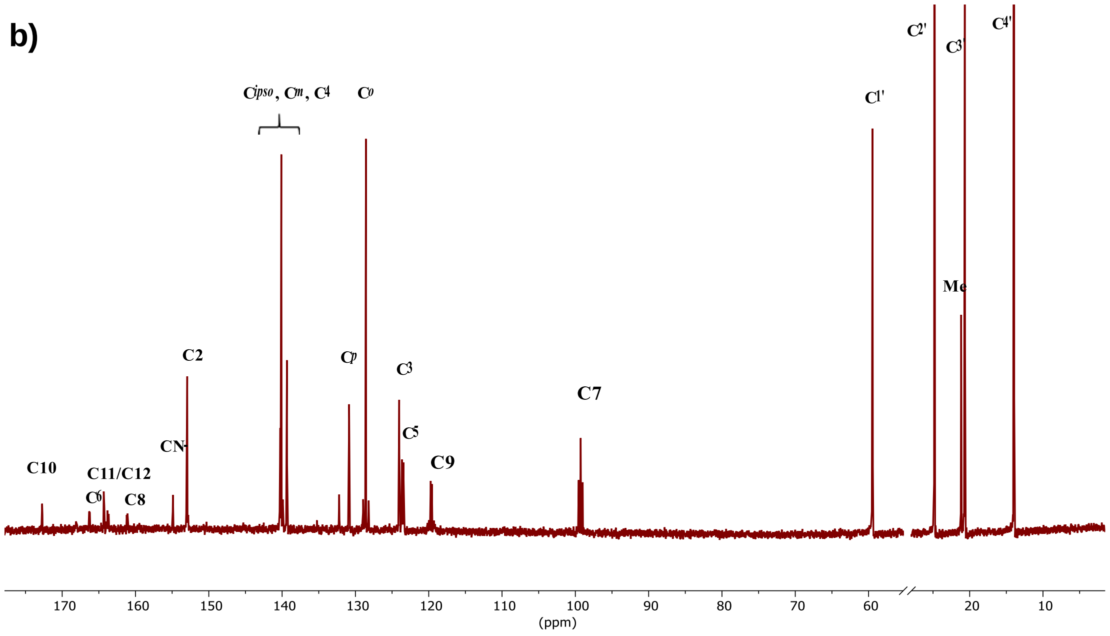
b)
C10
C6
C11/C12
C8
CN
C2
Cipso, Cm, C4
Co
Cp
C3
C5
C9
C7
C1'
C2'
C3'
C4'
Me
(ppm)

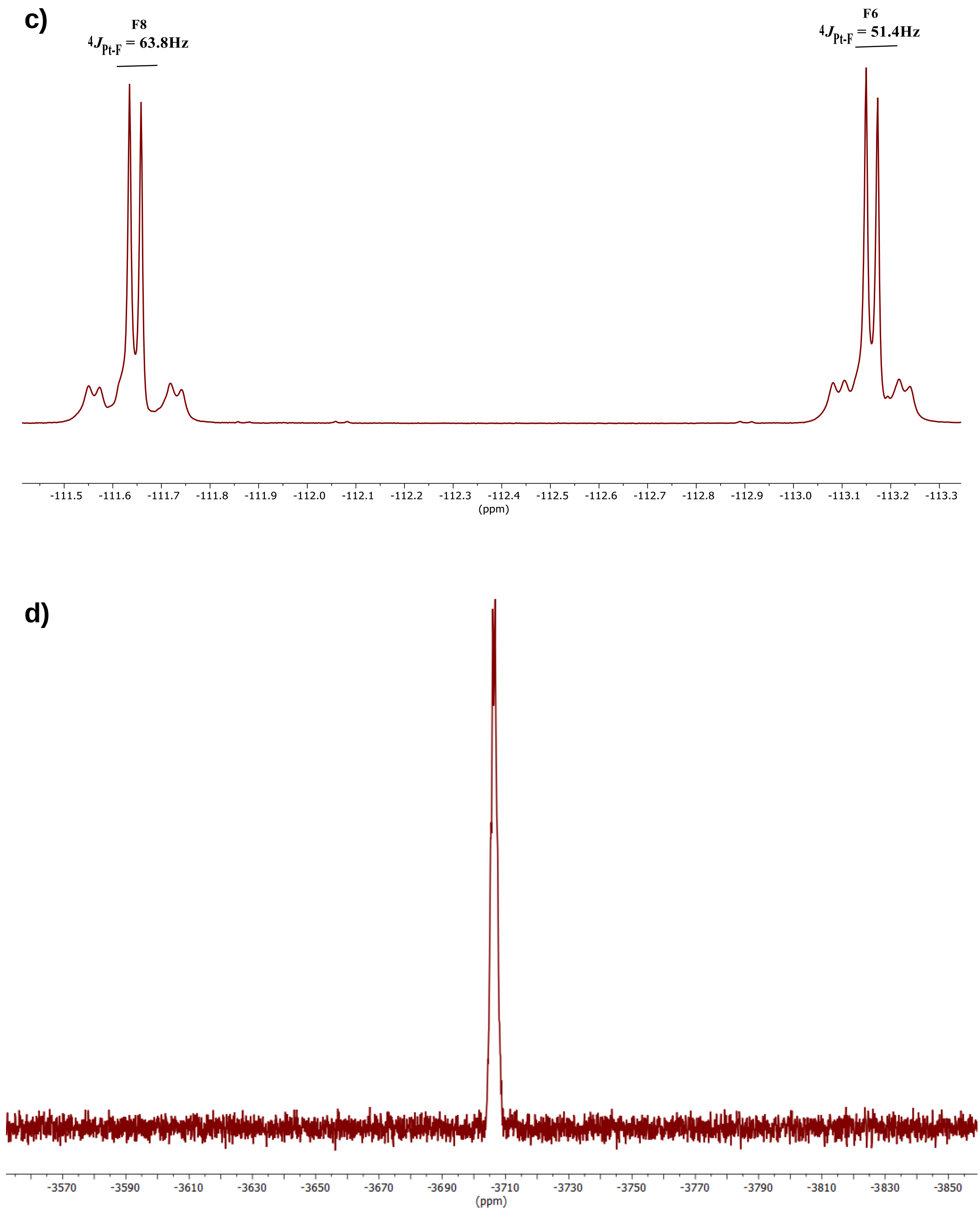

Figure S7. NMR spectra of $NBu_4[Pt(dfppy)(p\text{-}MeC_6H_4)(CN)]$ (**6**) in MeOD (a) ^{1}H (b) $^{13}C\{^{1}H\}$, (c) $^{19}F\{^{1}H\}$ and (d) ^{195}Pt.

a)

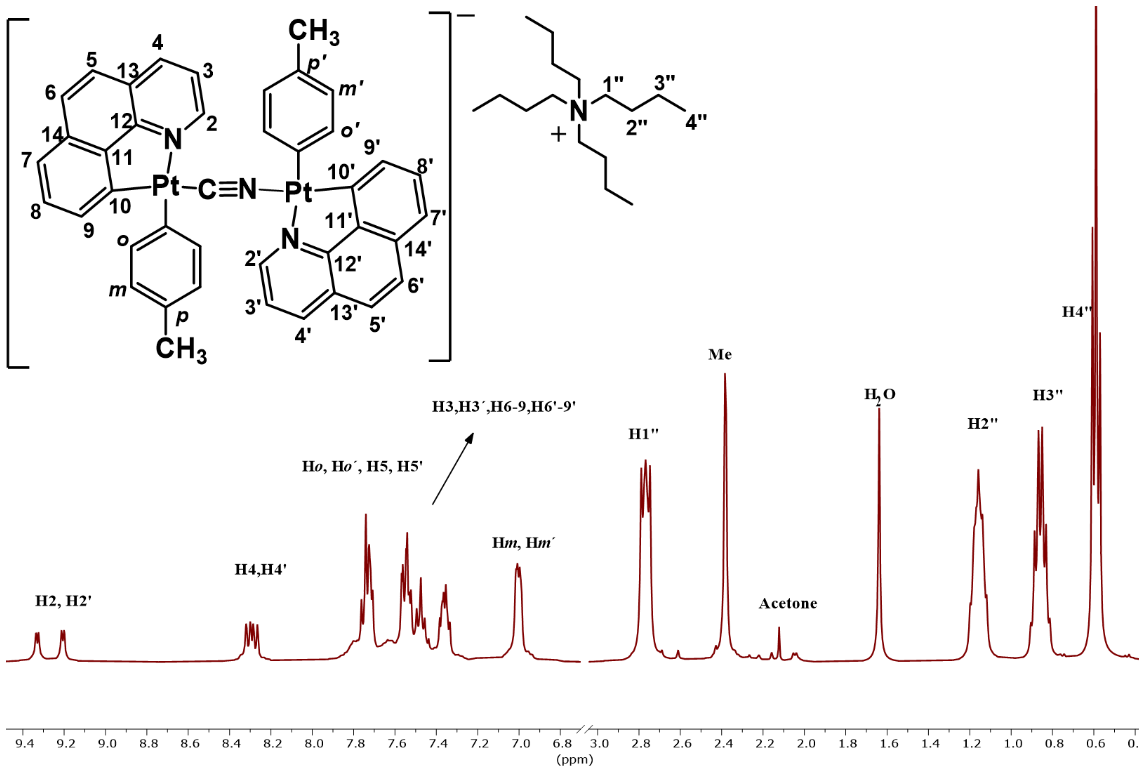
H2, H2'
H4,H4'
Ho, Ho´, H5, H5'
H3,H3´,H6-9,H6'-9'
Hm, Hm´
H1"
Me
Acetone
H2O
H2"
H3"
H4"
(ppm)

b)

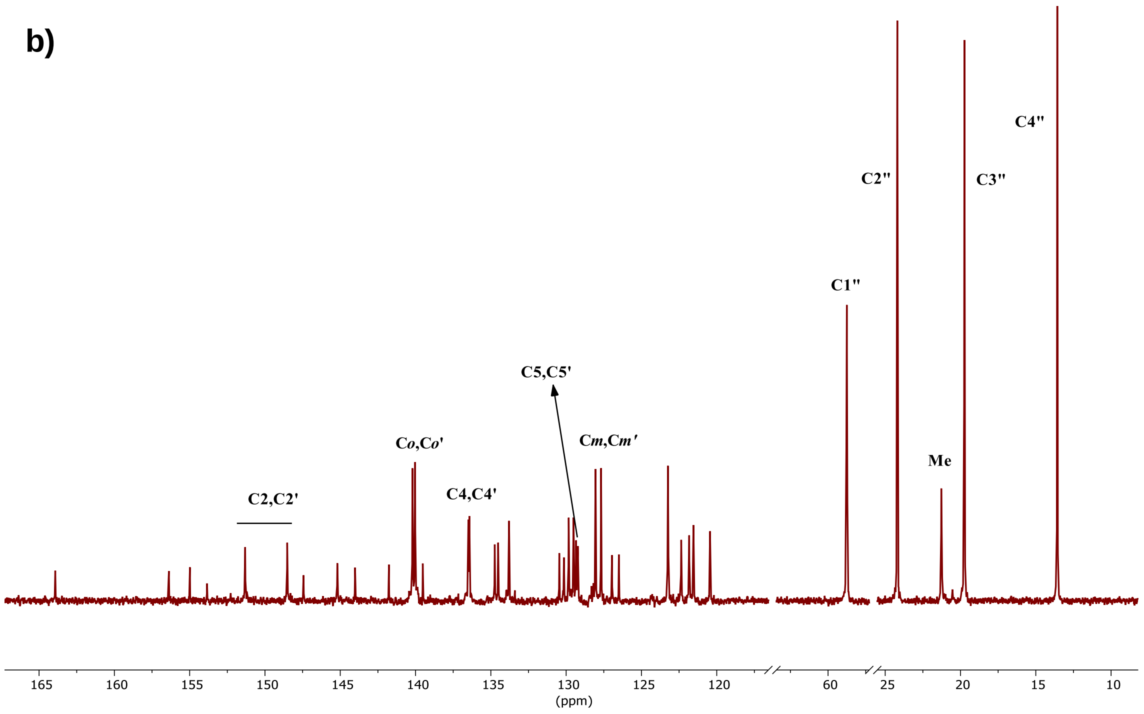
C2,C2'
Co,Co'
C4,C4'
C5,C5'
Cm,Cm'
C1"
C2"
Me
C3"
C4"
(ppm)

c)

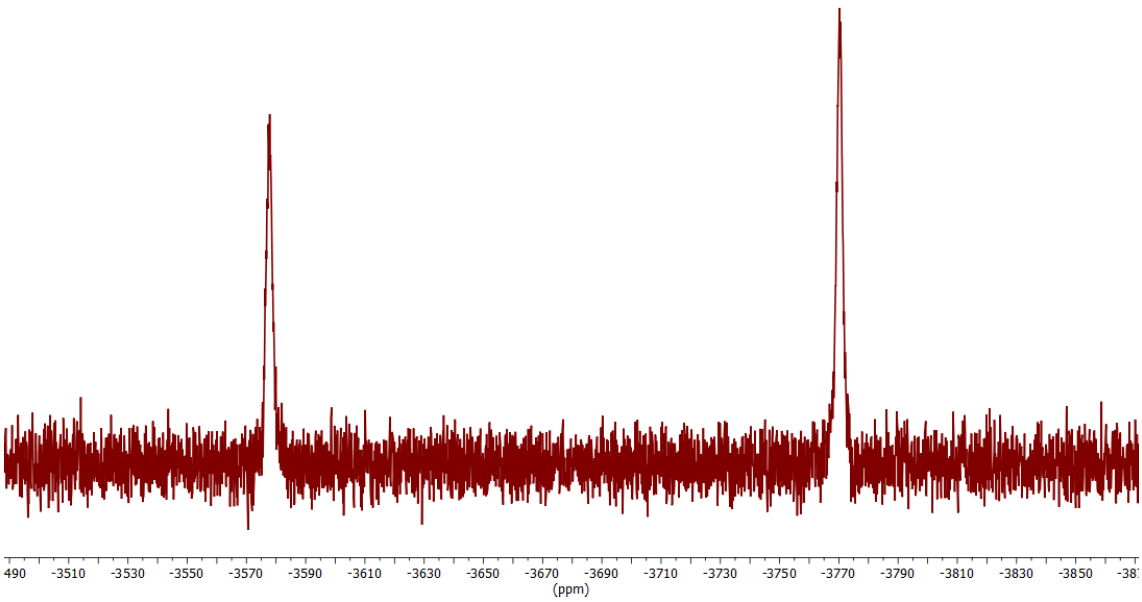

Figure S8. NMR spectra of $NBu_4[Pt_2(bzq)_2(p\text{-}MeC_6H_4)_2(\mu\text{-}CN)]$ (**7**) in CD_2Cl_2 (a) ^1H, (b) $^{13}C\{^1H\}$ and (c) ^{195}Pt.

a)

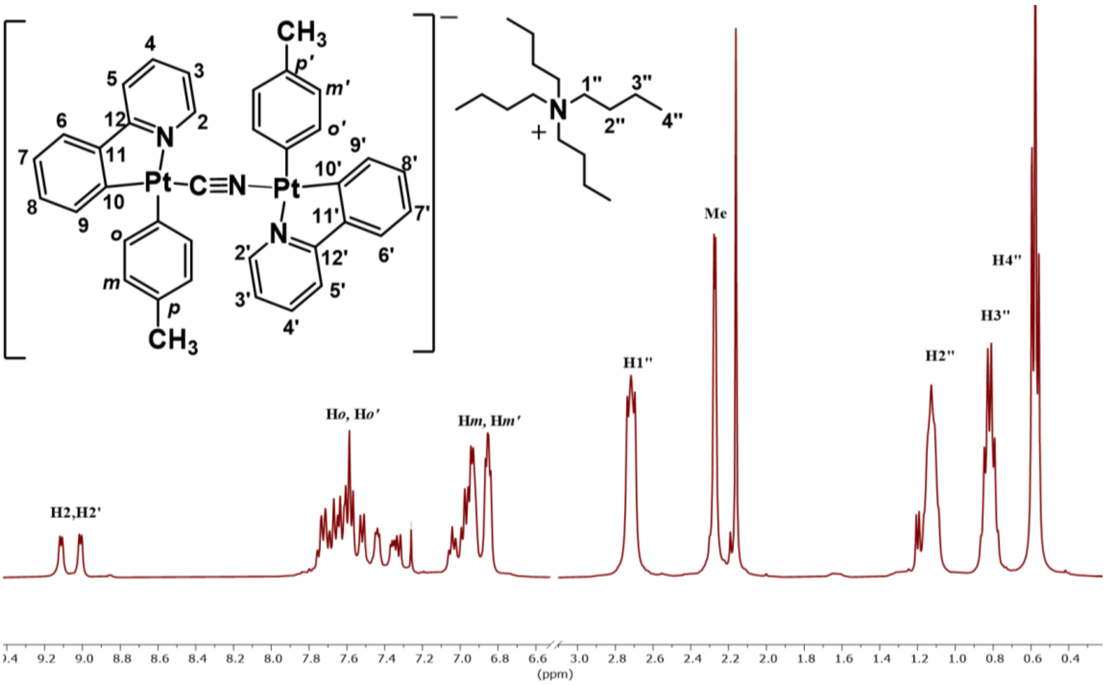

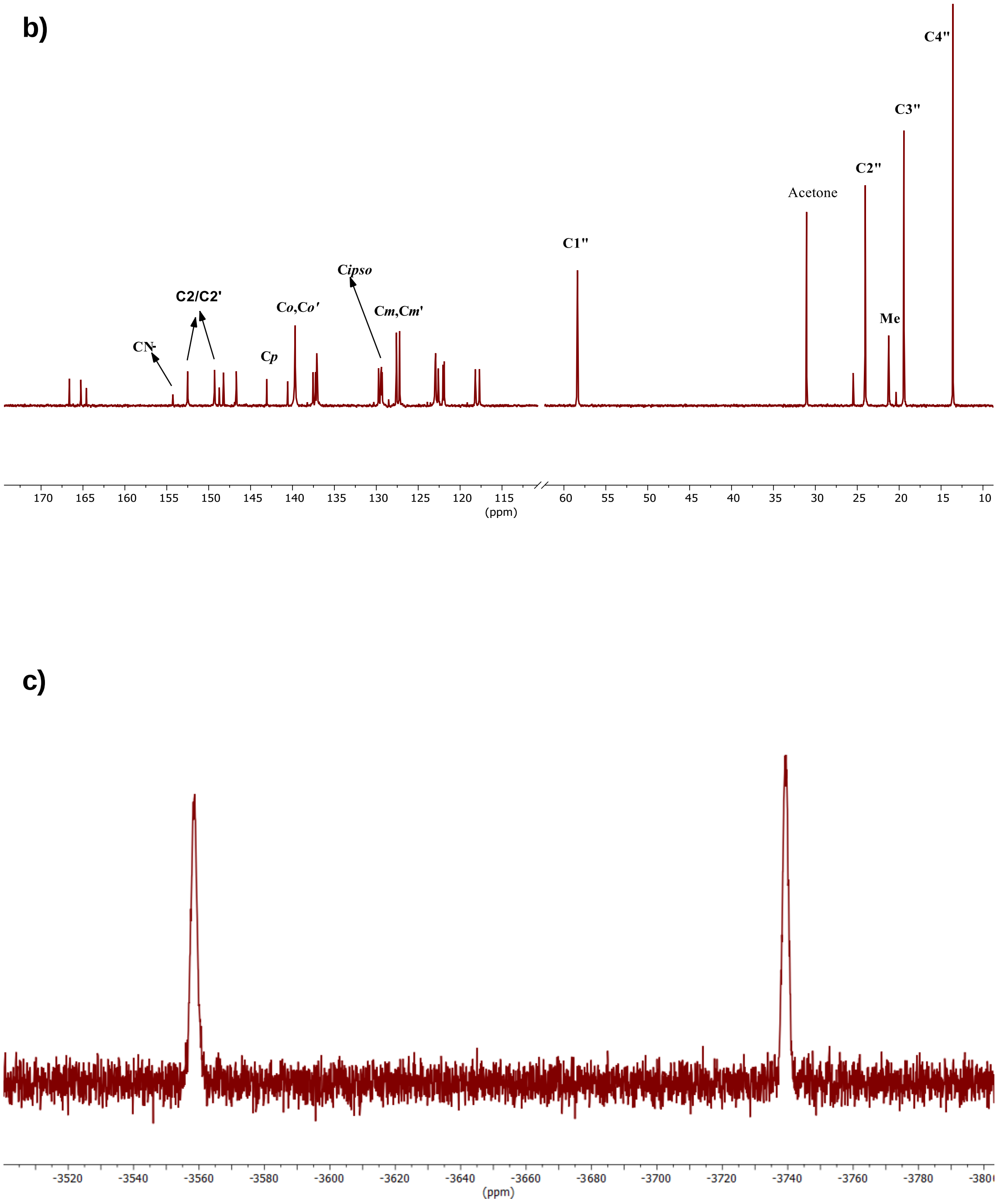

Figure S9. NMR spectra of $NBu_4[Pt_2(ppy)_2(p\text{-}MeC_6H_4)_2(\mu\text{-}CN)]$ (**8**) in $CDCl_3$ (a) ^1H, (b) $^{13}C\{^1H\}$ and (c) ^{195}Pt.

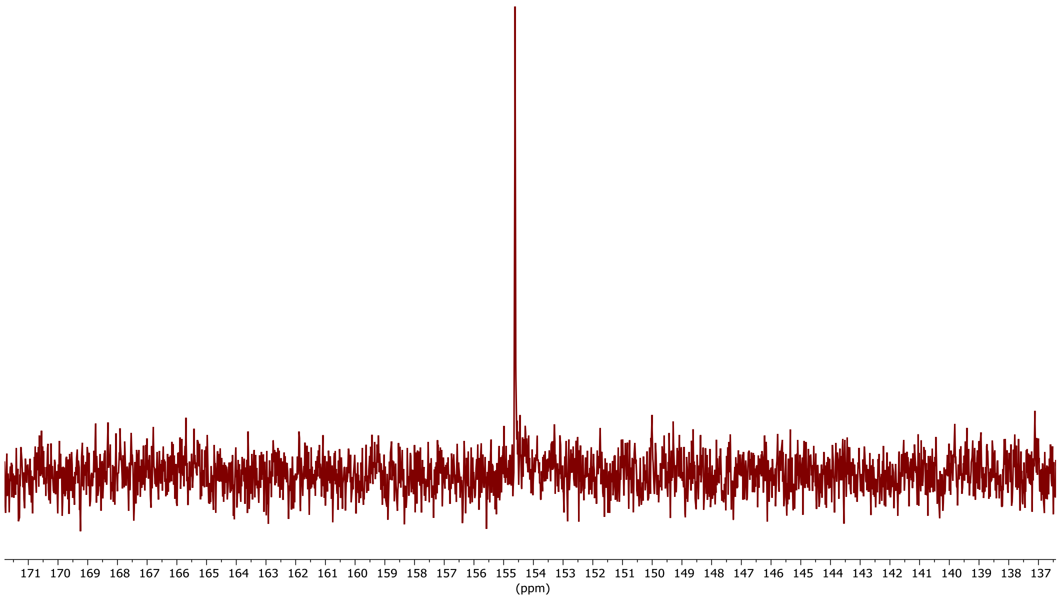

Figure S10. Selected region of the $^{13}C\{^1H\}$ NMR spectrum of $NBu_4[Pt_2(ppy)_2(p\text{-}MeC_6H_4)_2(\mu\text{-}^{13}CN)]$ (**8'**) in $CDCl_3$.

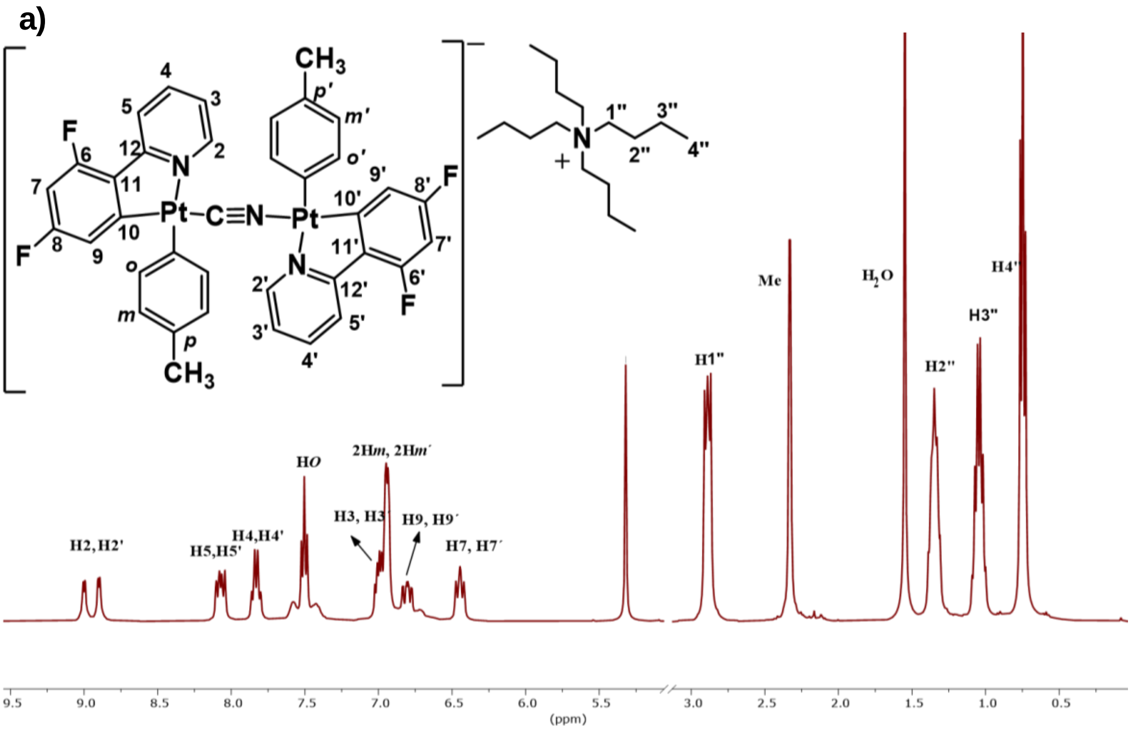

b)

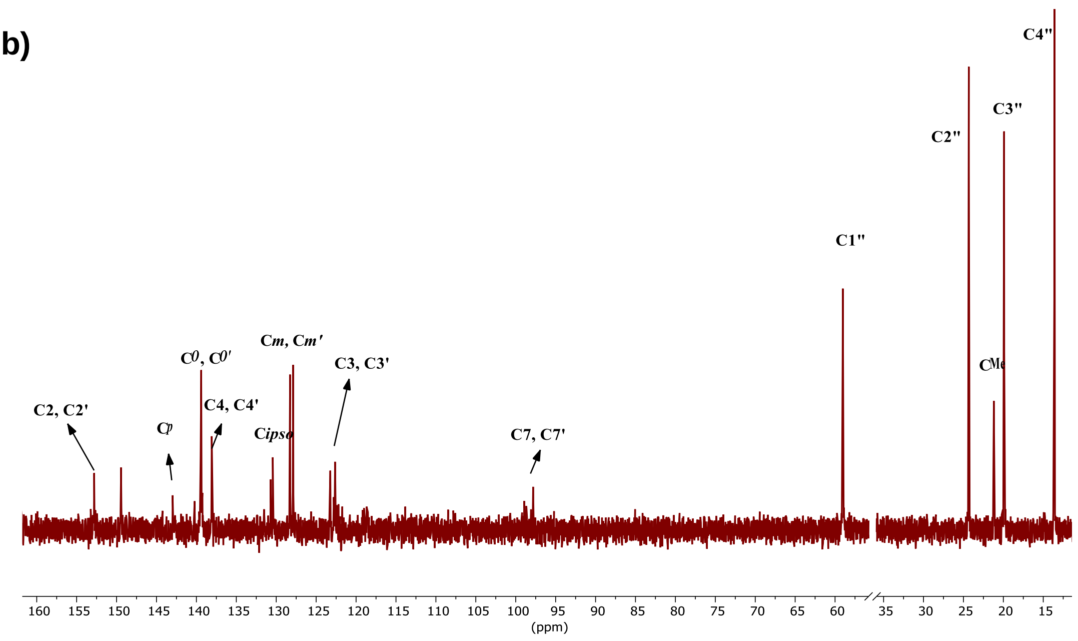
C4''
C2''
C3''
C1''
C0, C0'
Cm, Cm'
C3, C3'
CMe
C2, C2'
Cp
C4, C4'
Cipso
C7, C7'
160 155 150 145 140 135 130 125 120 115 110 105 100 95 90 85 80 75 70 65 60 35 30 25 20 15
(ppm)

c)

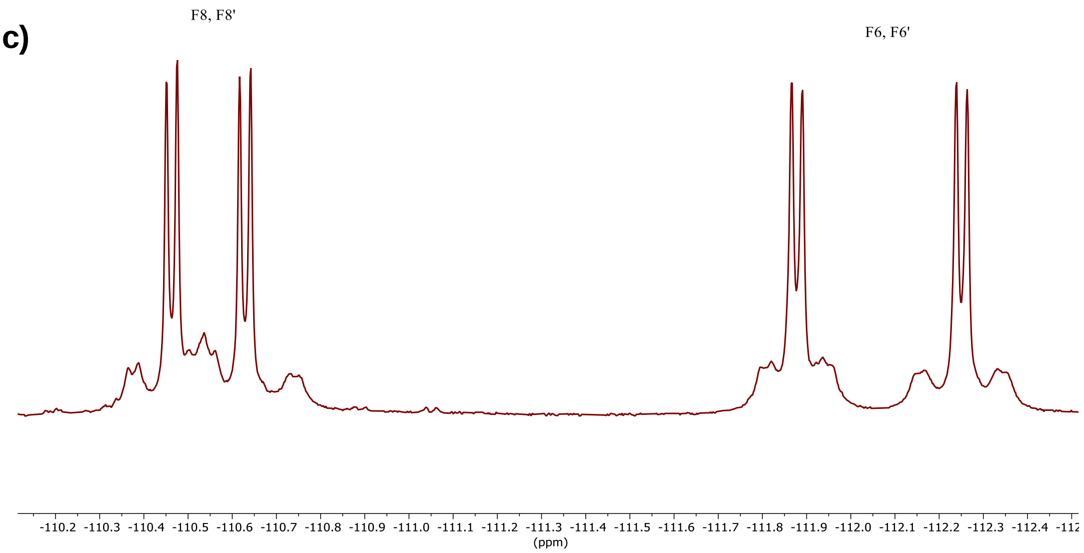
F8, F8'
F6, F6'
-110.2 -110.3 -110.4 -110.5 -110.6 -110.7 -110.8 -110.9 -111.0 -111.1 -111.2 -111.3 -111.4 -111.5 -111.6 -111.7 -111.8 -111.9 -112.0 -112.1 -112.2 -112.3 -112.4
(ppm)

d)

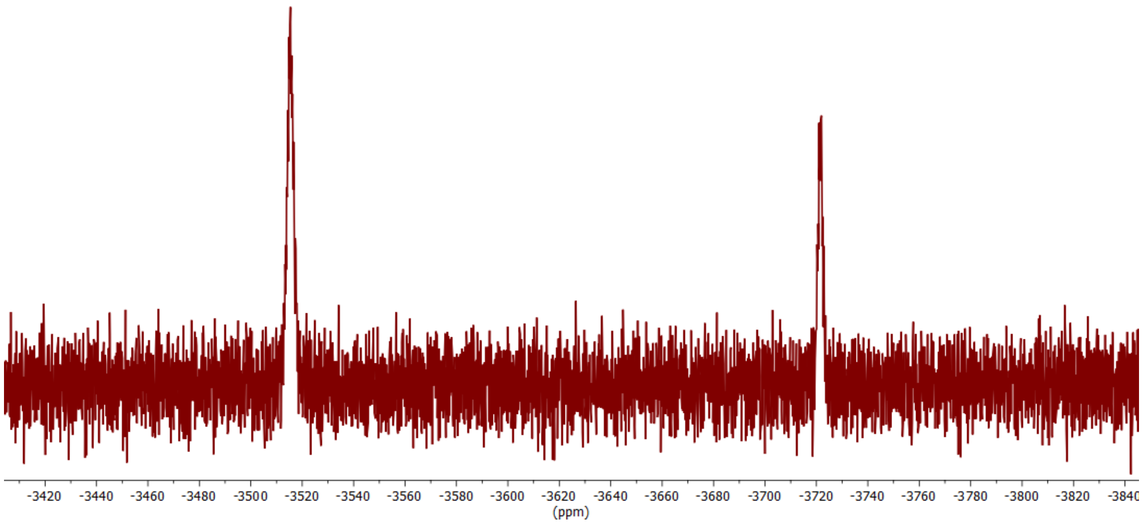

Figure S11. NMR spectra of $NBu_4[Pt_2(dfppy)_2(p\text{-}MeC_6H_4)_2(\mu\text{-}CN)]$ (**9**) in CD_2Cl_2 (a) ^1H, (b) $^{13}C\{^1H\}$, (c) $^{19}F\{^1H\}$ and (d) ^{195}Pt.

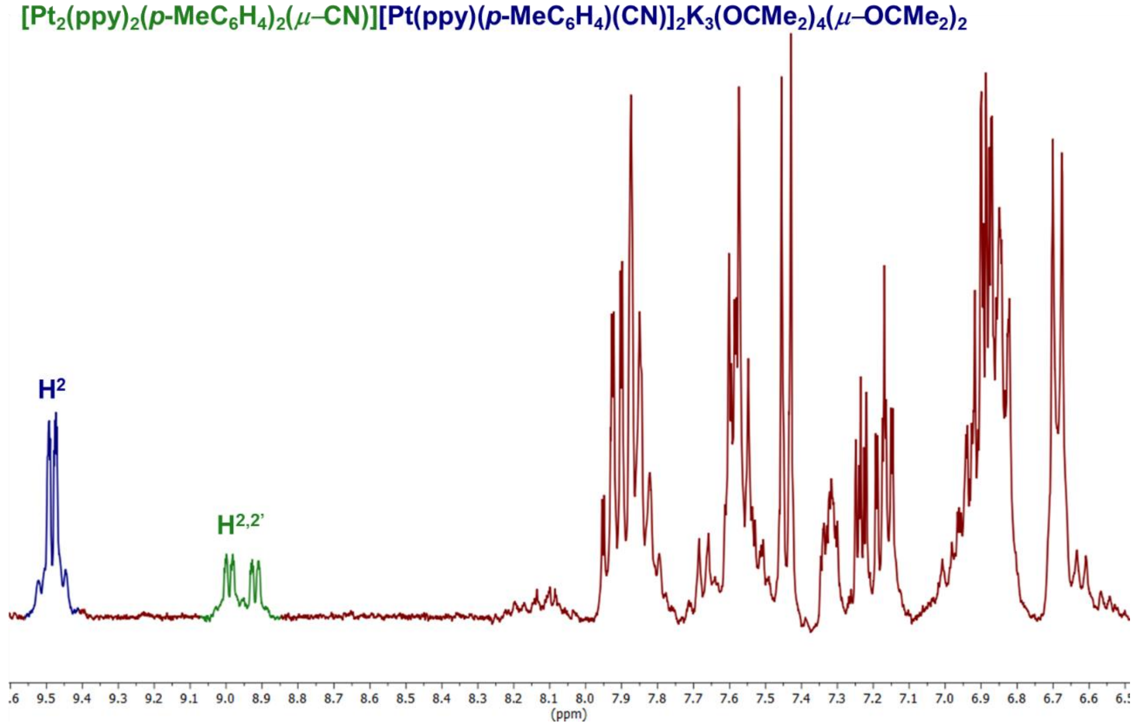

Figure S12. ^1H NMR spectra (aromatic region) of $\{[Pt(ppy)(p\text{-}MeC_6H_4)(CN)]_2K_3(OCMe_2)_4(\mu\text{-}OCMe_2)_2\}[Pt(ppy)(p\text{-}MeC_6H_4)(\mu\text{-}CN)Pt(ppy)(p\text{-}MeC_6H_4)]$ (**10**).

2.- Mass Spectra

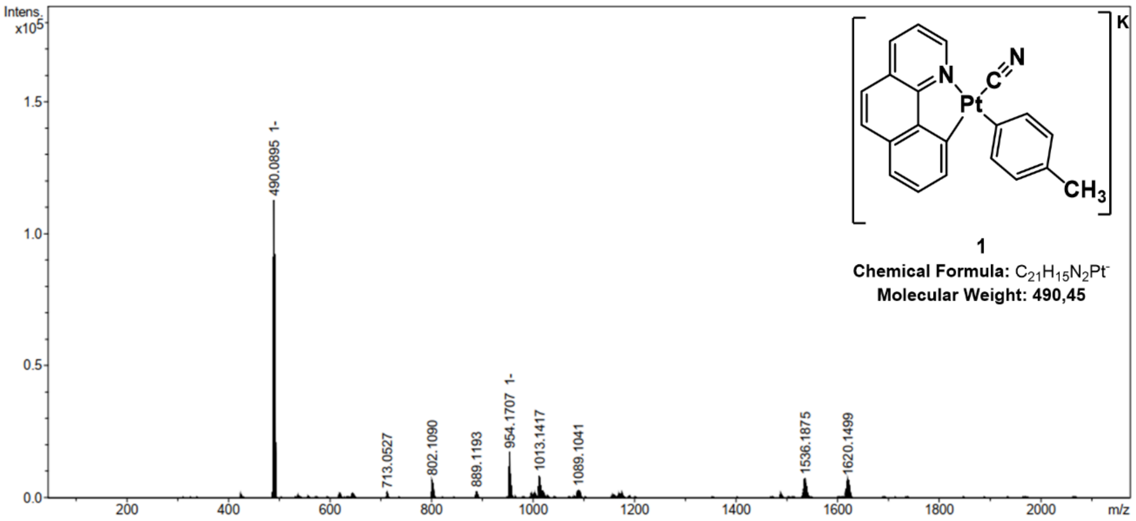

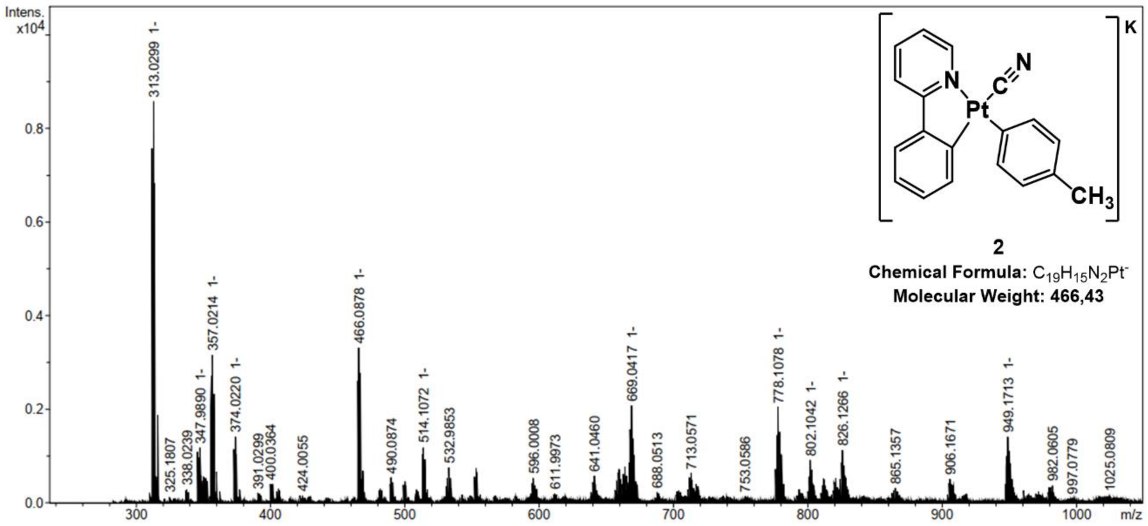

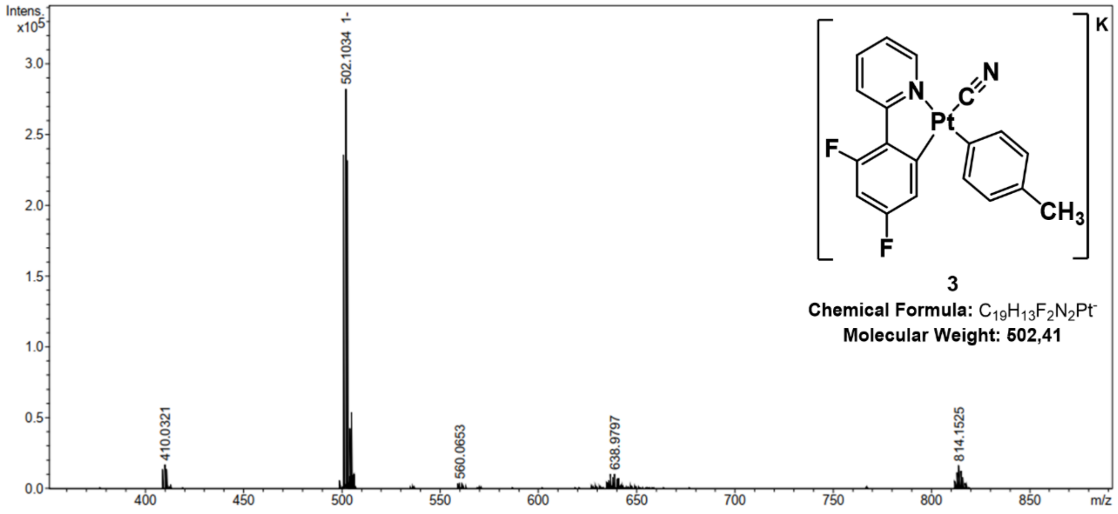

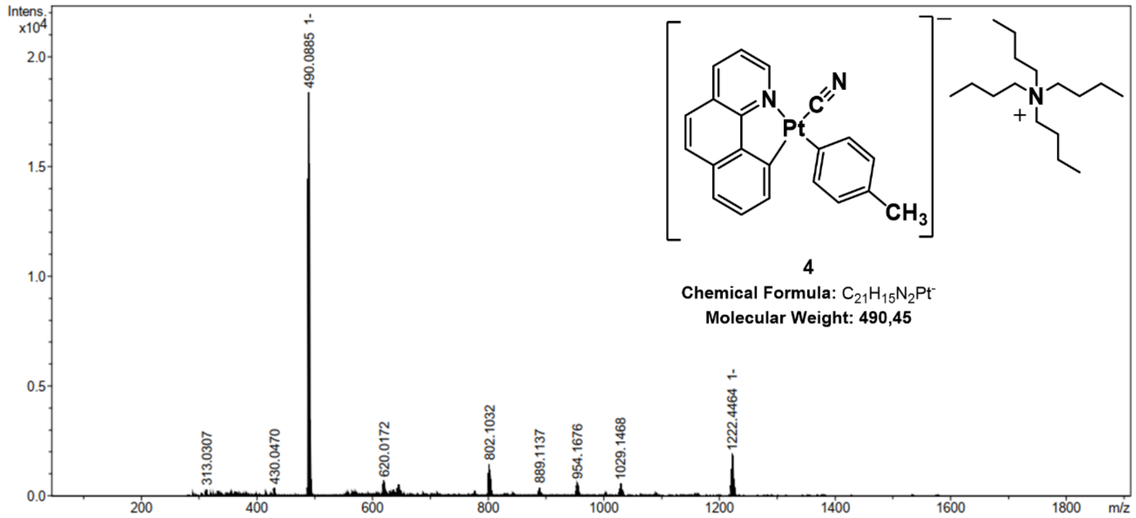

4
Chemical Formula: $C_{21}H_{15}N_2Pt^-$
Molecular Weight: 490,45

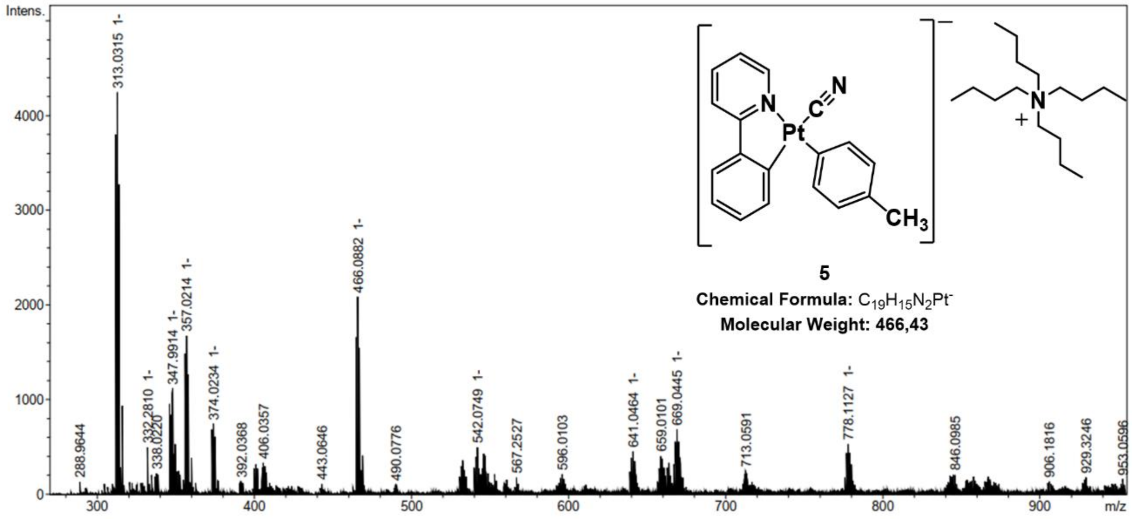

5
Chemical Formula: $C_{19}H_{15}N_2Pt^-$
Molecular Weight: 466,43

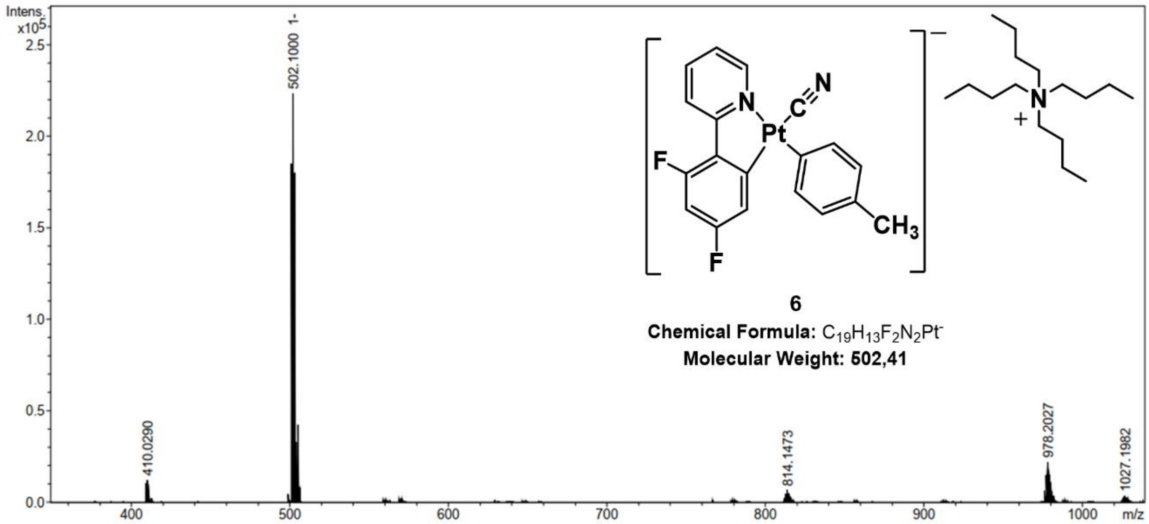

6
Chemical Formula: $C_{19}H_{13}F_2N_2Pt^-$
Molecular Weight: 502,41

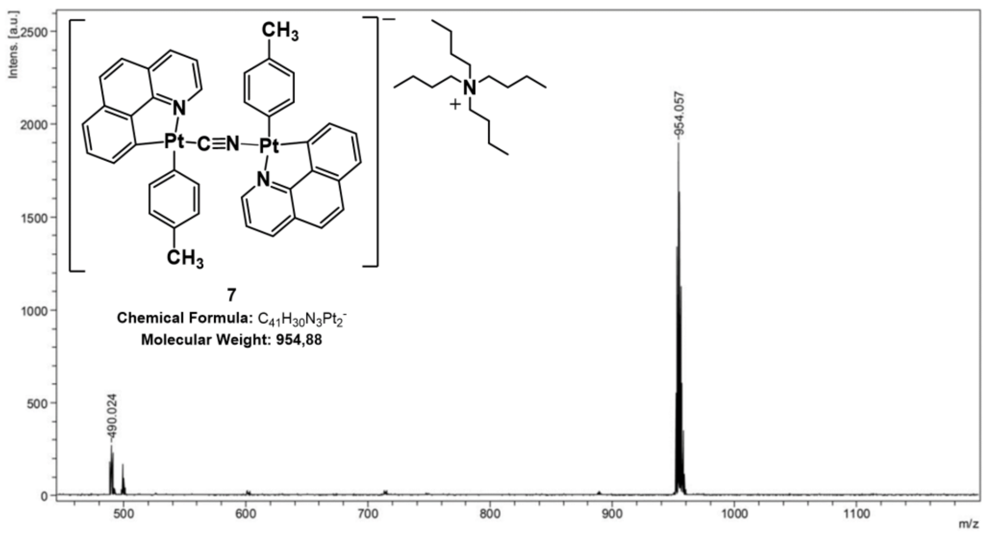

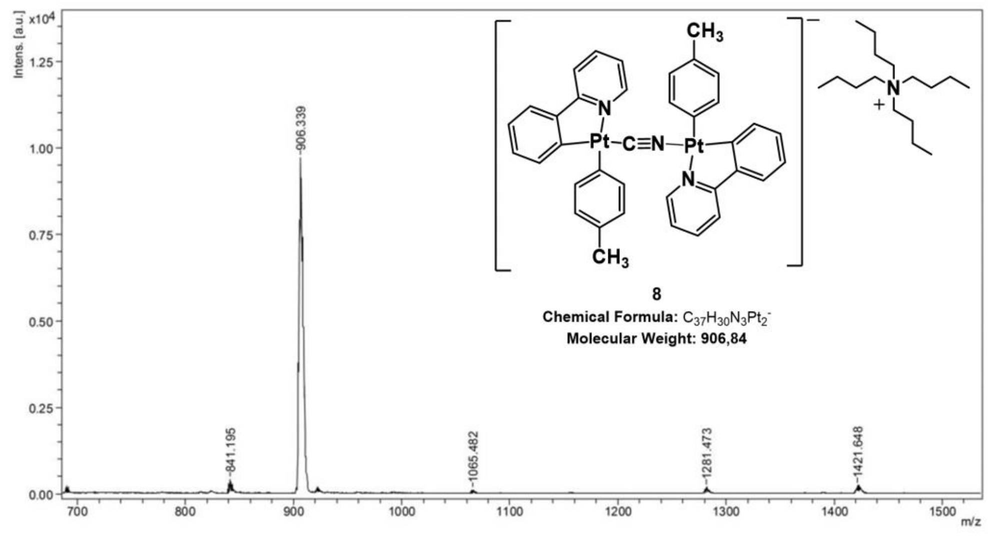

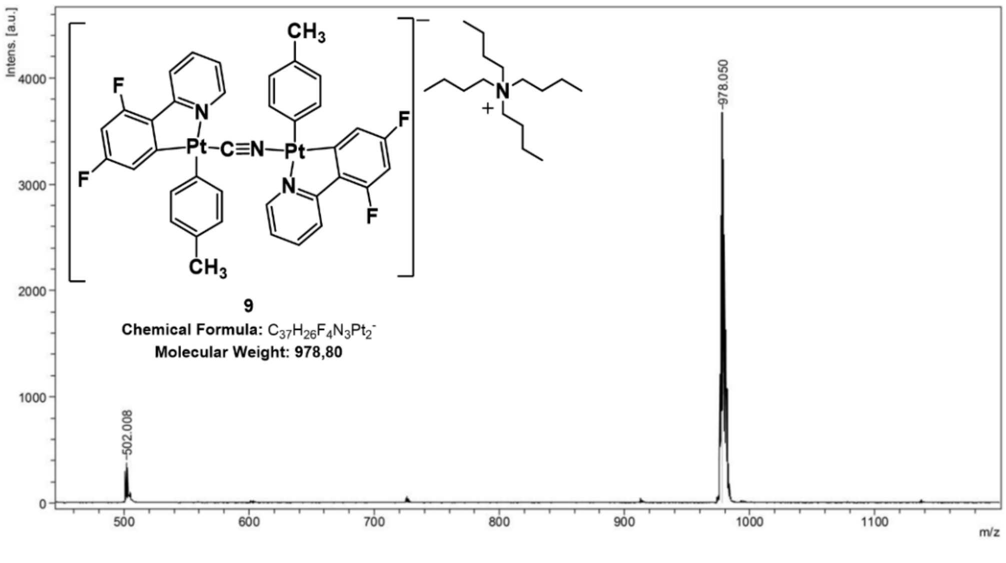

Figure S13. ESI(-) Mass spectra of **1**– **6** and MALDI-TOF(-) Mass spectra of **7**–**9**.

3.- Crystal Structures

Table S1. Crystal Data and Structure Refinement for complexes **5**–**10**

	**5**	**6**	**7**
**Empirical formula**	$C_{35}H_{51}N_3Pt$	$C_{35}H_{49}F_2N_3Pt$	$C_{60}H_{72}N_4OPt_2$
**Molecular weight**	708.87	744.86	1255.39
**T (K)**	100(2)	100(2)	100(2)
**λ (Å)**	0.71076	0.71076	0.71076
**Crystal system**	Monoclinic	Monoclinic	Monoclinic
**Space group**	$P\,2_1/c$	$P\,2_1/n$	$P\,2_1/c$
**Crystal size (mm)**	0.380 × 0.093 × 0.088	0.356 × 0.200 × 0.073	0.420 × 0.094 × 0.074
**a (Å)**	18.2445(9)	9.7033(8)	16.2856(7)
**b (Å)**	8.9701(5)	17.1974(12)	19.3253(8)
**c (Å)**	20.3780(11)	19.9595(15)	17.1112(6)
**α (°)**	90.0	90.0	90.0
**β (°)**	104.501	98.846(3)	100.7090(10)
**γ (°)**	90.0	90.0	90.0
**V ($Å^3$)**	3228.7(3)	3291.1(4)	5291.5(4)
**Z**	4	4	4
***ρ* (calculated) (Mg/m^3)**	1.458	1.503	1.576
**μ (mm^{-1})**	4.372	4.302	5.325
**F (000)**	1440	1504	2496
**θ range for data collection (°)**	2.098 to 31.421	2.432 to 27.932	2.423 to 26.374
**Index ranges**	-26 ≤ h ≤ 26, -11 ≤ k ≤ 11, -24 ≤ l ≤ 23	-12 ≤ h ≤ 12, -22 ≤ k ≤ 22, -26 ≤ l ≤ 26	-20 ≤ h ≤ 20, -24 ≤ k ≤ 24, -20 ≤ l ≤ 21
**Reflections collected**	208698	185044	320998
**Independent reflections**	7720 [R(int) = 0.0348]	7872 [R(int) = 0.0369]	10823 [R(int) = 0.0379]
**Data / restraints / parameters**	7720 / 1 / 352	7872 / 0 / 546	10823 / 0 / 607
**Goodness-of-fit on F^2 [a]**	1.087	1.277	1.156
**Final R indices [I>2σ(I)]** [a]	R1 = 0.0190, wR2 = 0.0500	R1 = 0.0216, wR2 = 0.0502	R1 = 0.0225, wR2 = 0.0451
**R indices (all data)** [a]	R1 = 0.0198, wR2 = 0.0507	R1 = 0.0224, wR2 = 0.0506	R1 = 0.0295, wR2 = 0.0523
**Largest diff. peak and hole (e $Å^{-3}$) (dmin/dmax)**	1.742 and -1.038	1.898 and -1.399	1.977 and -1.092

[a] $R1 = \sum(|F_o| - |F_c|)/\sum|F_o|$; $wR_2 = [\sum w(F_o^2 - F_c^2)^2/\sum wF_o^2]^{1/2}$; goodness of fit $= \{\sum[w(F_o^2 - F_c^2)^2]/(N_{obs} - N_{param})\}^{1/2}$; $w = [\sigma^2(F_o) + (g1P)^2 + g2P]^{-1}$; $P = [\max(F_o^2;0 + 2F_c^2)/3$.

Table S1(cont.). Crystal Data and Structure Refinement for complexes **5**–**10**

	**8**	**9**	**10**
**Empirical formula**	$C_{53}H_{66}N_4Pt_2$	$C_{53}H_{62}F_4N_4Pt_2$	$C_{50}H_{54}K_{1.50}N_3O_4Pt_2$
**Molecular weight**	1149.27	1221.24	1209.79
**T (K)**	100(2)	100(2)	100(2)
**λ (Å)**	0.71076	0.71076	0.71076
**Crystal system**	Monoclinic	Monoclinic	Triclinic
**Space group**	$P2_1/c$	$P2_1/c$	P -1
**Crystal size (mm)**	0.375 × 0.145 × 0.112	0.153 × 0.105 × 0.092	0.214 × 0.193 × 0.038
**a (Å)**	16.5799(10)	16.9159(8)	8.5365(5)
**b (Å)**	16.8150(12)	16.8391(9)	16.1683(12)
**c (Å)**	17.2431(12)	17.5770(9)	18.2033(13)
**α (°)**	90.0	90.0	69.981(3)
**β (°)**	89.993(2)	89.996(2)	81.802(3)
**γ (°)**	90.0	90.0	86.298(3)
**V (Å^3)**	4807.2(6)	5006.8(4)	2336.2(3)
**Z**	4	4	2
***ρ* (calculated) (Mg/m^3)**	1.588	1.620	1.720
**μ (mm^{-1})**	5.852	5.635	6.162
**F (000)**	2272	2400	1183
**θ range for data collection (°)**	2.362 to 29.013	2.317 to 29.043	2.322 to 26.373
**Index ranges**	$-22 \leq h \leq 22$, $-22 \leq k \leq 22$, $-21 \leq l \leq 21$	$-23 \leq h \leq 23$, $-22 \leq k \leq 22$, $-22 \leq l \leq 22$	$-10 \leq h \leq 10$, $-20 \leq k \leq 20$, $-22 \leq l \leq 22$
**Reflections collected**	305573	299413	137074
**Independent reflections**	11503 [R(int) = 0.0385]	11967 [R(int) =0.0372]	9564 [R(int) = 0.0475]
**Data / restraints / parameters**	11503 / 0 / 532	11967 / 0 / 568	9564 / 0 / 547
**Goodness-of-fit on F^2[a]**	1.365	1.156	1.094
**Final R indices [I>2σ(I)] [a]**	R1 = 0.0197, wR2 = 0.0424	R1 = 0.0179, wR2 = 0.0390	R1 = 0.0242, wR2 = 0.0588
**R indices (all data) [a]**	R1 = 0.0219, wR2 = 0.0435	R1 = 0.0215, wR2 = 0.0408	R1 = 0.0297, wR2 = 0.0622
**Largest diff. peak and hole (e Å^{-3}) (dmin/dmax)**	0.643 and -0.933	1.269 and -0.943	2.575 and -1.103

[a] $R1 = \sum(|F_o| - |F_c|)/\sum|F_o|$; $wR_2 = [\sum w(F_o^2 - F_c^2)^2/\sum wF_o^2]^{1/2}$; goodness of fit = $\{\sum[w(F_o^2 - F_c^2)^2]/(N_{obs} - N_{param})\}^{1/2}$; $w = [\sigma^2(F_o) + (g1P)^2 + g2P]^{-1}$; $P = [max(F_o^2;0 + 2F_c^2)/3$.

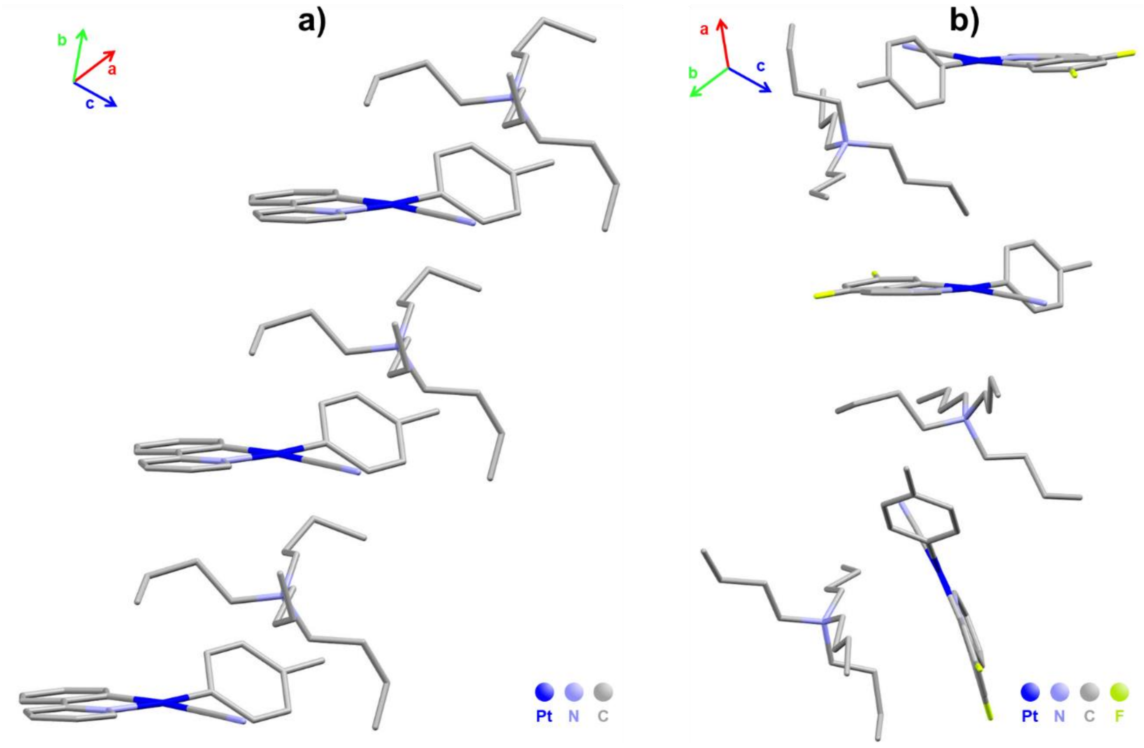

Figure S14. Crystal packing of (a) **5** and (b) **6**.

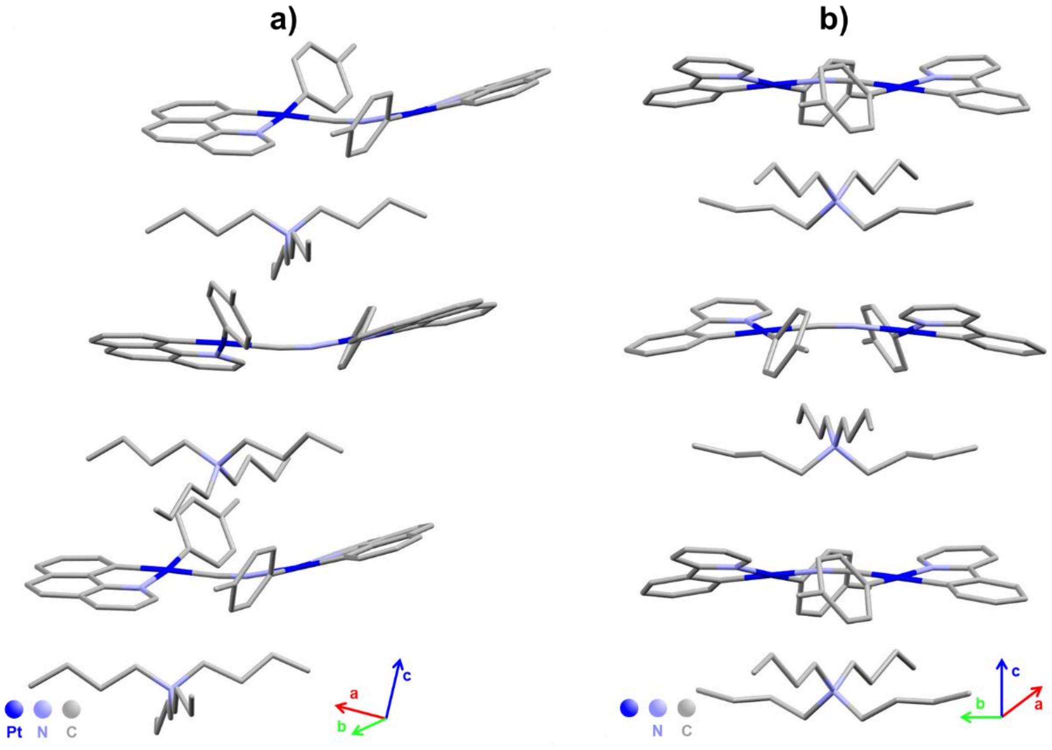

Figure S15. Crystal packing of (a) **7** and (b) **8**.

4.- Photophysical properties and Theoretical Calculations

Table S2. Experimental absorption data for complexes **1**–**6** in MeOH and **7**–**9** in mixture of MeOH/CH_2Cl_2 (5×10^{-5} M) and solid state at 298 K.

Compound	Media	λ_{abs}/nm (ε x 10^{-3} M^{-1} cm^{-1})
**1**	**MeOH**	215(50.0), 233(38.1), 256(26.2), 302(10.8), 374(4.3), 385(3.8), 410(2.0)
	**Solid**	298, 313, 317, 321, 331, 346, 349, 352, 358, 366, 379, 384, 392, 404, 414, 462$_{sh}$, 480$_{sh}$
**2**	**MeOH**	207(37.4), 241(23.4), 259(24.7), 275(23.0), 320(5.3), 327(4.8), 368(3.7), 360(3.8), 371(3.7), 401(1.4)
	**Solid**	294, 302, 312, 314, 326, 329,332,335, 349, 352, 356, 360, 362, 371, 376, 386, 388, 402, 444, 471, 488$_{sh}$
**3**	**MeOH**	205(43.6), 209(39.2), 238(28.2), 253(24.5), 321(6.3), 359(4.7), 371(3.9), 381(2.7), 402(0.1)
	**Solid**	281, 295, 305, 307, 309, 322, 326, 332, 337, 341, 346, 363, 430, 456, 469$_{sh}$
**4**	**MeOH**	214(48.7), 240(37.6), 257(29.2), 305(11.5), 379(4.3), 401(2.6), 410(2.1), 420(1.2)
	**Solid**	295, 299, 301, 309, 312, 315, 323, 325, 329, 332, 340, 346, 360, 363, 367, 370, 385, 389, 398, 464, 475$_{sh}$
**5**	**MeOH**	208(40.7), 238(28.0), 257(26.3), 278(21.1), 326(6.1), 364(5.5), 390(2.9), 400(1.6), 405(1.2)
	**Solid**	299, 302, 307, 313, 320, 324, 328, 331, 338, 346, 358, 378, 383, 393, 455, 475, 492$_{sh}$
**6**	**MeOH**	204(39.6), 208(40.8), 237(10.6), 248(26.7), 252(26.4), 267(23.2), 321(7.14), 359(5.5), 369(4.9), 381(3.06), 393(1.5), 400(0.7)
	**Solid**	278, 284, 306, 312, 320, 339, 341, 350, 352, 362, 367, 381, 430, 462
**7**	**MeOH/CH_2Cl_2**	224(80.7), 242(80.1), 258(64.9), 289(26.8), 302(26.1), 340(20.8), 375(10.8), 406(5.5), 420(3.9),435(2.1)
	**Solid**	274, 285, 300, 307, 314, 317, 357, 394, 440, 475, 490$_{sh}$
**8**	**MeOH/CH_2Cl_2**	217(58.7), 239(52.0), 259(51.3), 274(47.7), 325(12.9), 365(10.6), 379(9.1), 385(7.7), 400(4.3), 418(1.8), 423(1.3)
	**Solid**	285, 317, 324, 380, 412, 477, 490$_{sh}$
**9**	**MeOH/CH_2Cl_2**	204(45.5), 217(53.6), 239(59.5), 253(54.9), 275(50.8), 321(15.1), 364(12.0), 380(8.8), 390(5.4), 400(2.8), 404(2.0)
	**Solid**	284, 298, 305, 312, 316, 323, 360, 371, 375, 428, 459, 465$_{sh}$

Table S3. Selected vertical excitation energies singlets (S_n) and first triplets computed by TDDFT/SCRF (MeOH) with the orbitals involved for **4**$^-$ – **6**$^-$.

[Pt(bzq)(*p*-MeC$_6$H$_4$)(CN)]$^-$			
**State**	**λ/nm**	***f***	**Transition (% Contribution)**
**T$_1$**	467.04	-	H-3→LUMO (23%), H-1→LUMO (13%), H-1→L+1 (33%)
**T$_2$**	439.15	-	H-1→LUMO (78%), H-1→L+1 (13%)
**T$_3$**	405.38	-	H-1→L+1 (16%), HOMO→LUMO (64%)
**S$_1$**	397.44	0.01	H-1→LUMO (80%), HOMO→LUMO (16%)
**S$_2$**	385.91	0.0818	H-1→LUMO (15%), HOMO→LUMO (81%)
**S$_3$**	368.23	0.0044	H-2→LUMO (98%)
**S$_4$**	336.83	0.0108	H-3→LUMO (11%), H-1→L+1 (33%), HOMO → L+1 (51%)
**S$_5$**	335.55	0.0335	H-3→LUMO (15%), H-1→L+1 (35%), HOMO→L+1 (45%)
**S$_6$**	319.39	0.1701	H-5→LUMO (10%), H-3→LUMO (56%), H-1→L+1 (25%)
**S$_9$**	299.40	0.0566	H-6->LUMO (35%), H-3->L+1 (46%)
**S$_{11}$**	281.65	0.0569	H-7→LUMO (21%), H-6→LUMO (23%), H-5→L+1 (19%), H-3→L+1 (24%)
**S$_{12}$**	280.89	0.0232	H-7→LUMO (66%)
[Pt(ppy)(*p*-MeC$_6$H$_4$)(CN)]$^-$			
**State**	**λ/nm**	***f***	**Transition (% Contribution)**
**T$_1$**	448.67		H-3→LUMO (10%), H-1→LUMO (67%)
**T$_2$**	388.52		H-1→LUMO (11%), HOMO→LUMO (75%)
**T$_3$**	375.45		H-2→LUMO (89%)
**S$_1$**	381.41	0,0003	H-1→LUMO (30%), HOMO→LUMO (68%)
**S$_2$**	371.81	0,0653	H-1→LUMO (66%), HOMO→LUMO (29%)
**S$_3$**	358.05	0,0091	H-2→LUMO (97%)
**S$_4$**	317.06	0,0457	H-4→LUMO (15%), H-3→LUMO (64%), H-1→L+1 (10%)
**S$_5$**	311.61	0,0653	H-3→LUMO (23%), H-1→L+1 (40%), HOMO→L+1 (30%)
**S$_8$**	290.41	0,2029	H-4→LUMO (61%), H-3→L+1 (10%)
**S$_{11}$**	267.61	0,0883	H-3→L+1 (63%), HOMO→L+2 (13%)
**S$_{12}$**	266.77	0,0545	H-3→L+1 (11%), HOMO→L+2 (82%)
[Pt(dfppy)(*p*-MeC$_6$H$_4$)(CN)]$^-$			
**State**	**λ/nm**	***f***	**Transition (% Contribution)**
**T$_1$**	433.24		H-3→LUMO (18%), H-1→LUMO (60%)
**T$_2$**	385.49		HOMO→LUMO (79%)
**T$_3$**	374.07		H-2→LUMO (92%)
**S$_1$**	376.81	0.0082	HOMO→LUMO (91%)
**S$_2$**	360.91	0.0487	H-1→LUMO (85%)
**S$_3$**	355.82	0.0123	H-2→LUMO (93%)
**S$_4$**	313.96	0.0833	H-4→LUMO (31%), H-3→LUMO (61%)
**S$_5$**	304.01	0.0332	H-4→LUMO (14%), H-3→LUMO (17%), H-1→L+1 (14%), HOMO→L+1 (49%)
**S$_6$**	298.26	0.0431	H-4→LUMO (16%), H-1→L+1 (25%), HOMO→L+1 (47%)
**S$_8$**	288.44	0.1656	H-4→LUMO (26%), H-1→L+1 (54%)
**S$_9$**	286.56	0.0171	H-5→LUMO (90%)
**S$_{12}$**	262.61	0.1078	H-4→L+1 (17%), H-3→L+1 (57%)

Table S4. Composition (%) of Frontier MOs in terms of ligands and metals in the ground state in MeOH for **4**$^-$–**6**$^-$.

[Pt(bzq)(*p*-MeC_6H_4)(CN)]$^-$					
**MO**	**eV**	**Pt**	**bzq**	**CN^-**	***p*-MeC_6H_4**
LUMO+5	0.80	17	5	1	77
LUMO+4	0.50	32	32	4	32
LUMO+3	0.46	11	9	1	79
LUMO+2	-0.08	14	80	3	3
LUMO+1	-1.02	6	92	2	0
LUMO	-1.55	4	95	0	1
HOMO	-5.32	25	7	2	66
HOMO-1	-5.37	31	62	6	1
HOMO-2	-5.70	87	4	0	10
HOMO-3	-6.03	43	48	3	6
HOMO-4	-6.25	11	3	1	85
HOMO-5	-6.34	38	46	5	11
**[Pt(ppy)(*p*-MeC_6H_4)(CN)]$^-$**					
**MO**	**eV**	**Pt**	**ppy**	**CN^-**	***p*-MeC_6H_4**
LUMO+5	0.92	47	39	1	12
LUMO+4	0.73	6	12	0	81
LUMO+3	0.48	3	4	0	93
LUMO+2	0.07	35	53	7	4
LUMO+1	-0.75	1	99	0	0
LUMO	-1.39	7	91	1	1
HOMO	-5.32	24	6	3	67
HOMO-1	-5.44	39	52	8	1
HOMO-2	-5.68	87	4	0	9
HOMO-3	-6.07	58	36	2	4
HOMO-4	-6.21	26	58	1	15
HOMO-5	-6.24	6	11	1	82
**[Pt(dfppy)(*p*-MeC_6H_4)(CN)]$^-$**					
**MO**	**eV**	**Pt**	**dfppy**	**CN^-**	***p*-MeC_6H_4**
LUMO+5	0.86	57	27	2	14
LUMO+4	0.68	10	12	0	78
LUMO+3	0.44	2	4	0	93
LUMO+2	-0.10	27	65	6	2
LUMO+1	-0.73	1	99	0	0
LUMO	-1.45	8	90	1	1
HOMO	-5.39	23	5	3	70
HOMO-1	-5.65	42	49	9	1
HOMO-2	-5.77	87	3	0	10
HOMO-3	-6.12	32	62	2	3
HOMO-4	-6.25	50	32	1	16
HOMO-5	-6.28	7	9	0	83

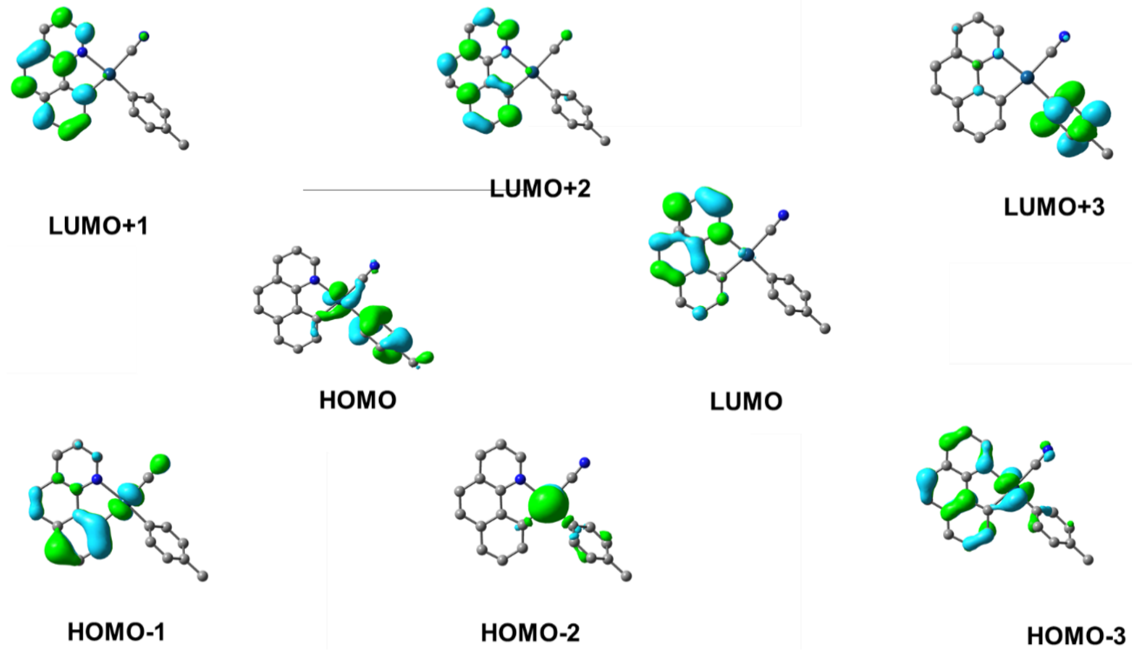
LUMO+1
LUMO+2
LUMO+3
HOMO
LUMO
HOMO-1
HOMO-2
HOMO-3

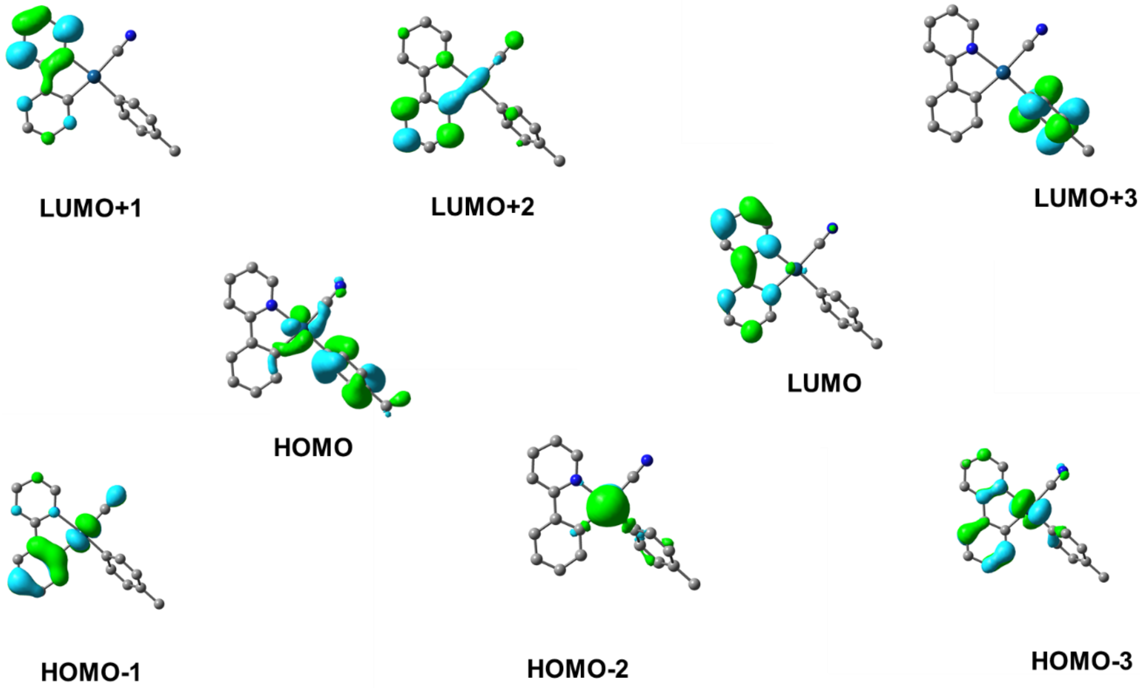
LUMO+1
LUMO+2
LUMO+3
LUMO
HOMO
HOMO-1
HOMO-2
HOMO-3

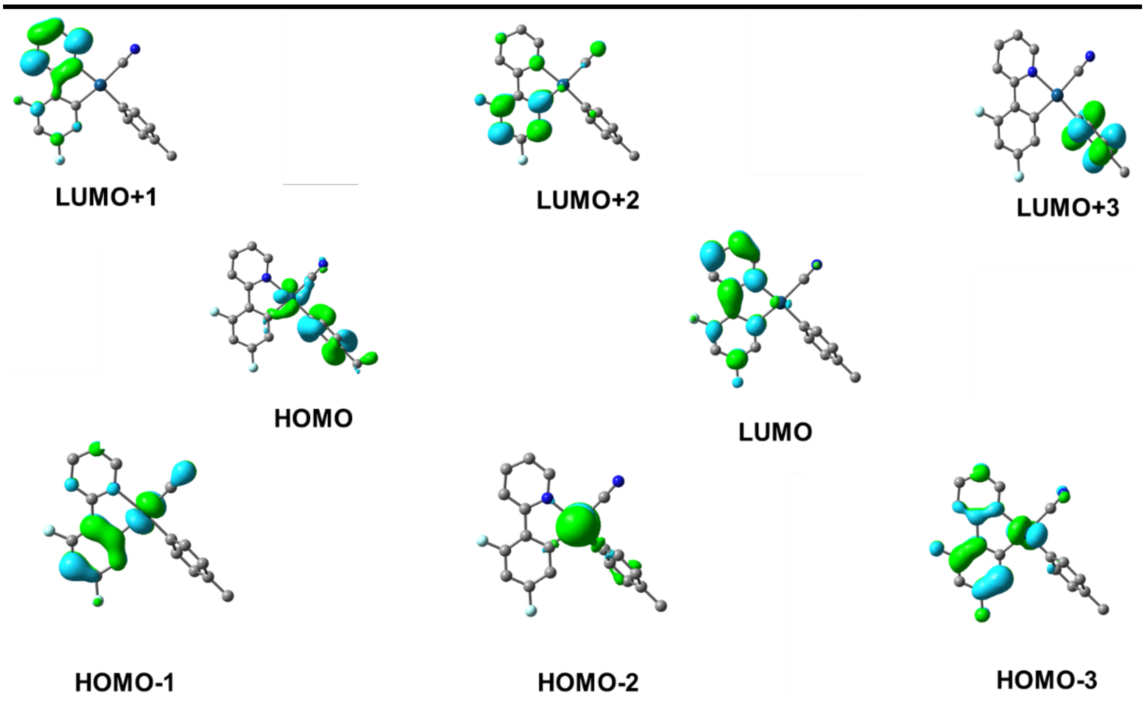

Figure S16. Selected frontier molecular orbitals for (a) **4**$^-$, (b) **5**$^-$ and (c) **6**$^-$ in the ground state in solution (MeOH).

Table S5. Selected vertical excitation energies singlets (S_n) and first triplets computed by TDDFT/SCRF (MeOH) with the orbitals involved for **7**$^-$–**9**$^-$.

**[Pt_2(bzq)$_2$(*p*-MeC_6H_4)$_2$(μ-CN)]$^-$**			
**State**	**λ/nm**	***f***	**Transition (% Contribution)**
**T_1**	468.09		H-6→L+1 (14%), HOMO→L+2 (13%), HOMO→L+3 (14%)
**T_2**	464.52		H-7→LUMO (17%), H-3→L+2 (19%)
**T_3**	447.82		H-3→L+1 (11%), HOMO→LUMO (32%), HOMO→L+1 (37%)
**S_1**	408.14	0.0284	HOMO→LUMO (45%), HOMO→L+1 (42%)
**S_2**	395.52	0.0129	H-3→LUMO (18%), HOMO→LUMO (34%), HOMO→L+1 (38%)
**S_3**	380.30	0.0252	H-1→LUMO (49%), H-1→L+1 (38%)
**S_4**	377.02	0.0933	H-2→LUMO (69%), H-1→L+1 (14%)
**S_9**	354.11	0.0244	H-3→LUMO (62%), H-3→L+1 (10%), HOMO→LUMO (11%)
**S_{10}**	343.78	0.1849	HOMO→L+2 (62%), HOMO→L+3 (19%)
**S_{11}**	342.24	0.0376	H-3→LUMO (11%), H-3→L+1 (75%)
**S_{12}**	334.78	0.0265	H-3→L+2 (12%), HOMO→L+2 (15%), HOMO→L+3 (42%)

$[Pt_2(ppy)_2(p\text{-}MeC_6H_4)_2(\mu\text{-}CN)]^-$			
**State**	**λ/nm**	***f***	**Transition (% Contribution)**
**T_1**	455.51		HOMO→LUMO (32%), HOMO→L+1 (29%)
**T_2**	445.87		H-4→LUMO (19%), H-3→LUMO (18%), HOMO→LUMO (11%), HOMO→L+1 (16%)
**T_3**	388.26		H-1→LUMO (29%), H-1→L+1 (34%)
**S_1**	390.75	0.0539	HOMO→LUMO (62%), HOMO→L+1 (26%)
**S_2**	376.27	0.0115	H-2→LUMO (11%), H-1→LUMO (12%), HOMO→LUMO (16%), HOMO→L+1 (37%)
**S_3**	368.97	0.0101	H-1→LUMO (47%), H-1→L+1 (23%), HOMO→L+1 (17%)
**S_4**	368.30	0.1009	H-2→LUMO (65%), H-1→L+1 (14%)
**S_9**	332.85	0.0196	H-4→LUMO (31%), H-3→LUMO (34%), HOMO→L+1 (13%)
**S_{11}**	318.99	0.0523	H-4→L+1 (35%), H-3→L+1 (27%)
$[Pt_2(dfppy)_2(p\text{-}MeC_6H_4)_2(\mu\text{-}CN)]^-$			
**State**	**λ/nm**	***f***	**Transition (% Contribution)**
**T_1**	438.39		H-5→L+1 (14%), H-2→LUMO (18%), H-2→L+1 (14%)
**T_2**	431.67		H-5→LUMO (31%)
**T_3**	384.89		H-2→LUMO (14%), H-2→L+1 (14%), HOMO→LUMO (27%), HOMO→L+1 (24%)
**S_1**	379.18	0.018	H-2→LUMO (15%), HOMO→LUMO (40%), HOMO→L+1 (23%)
**S_2**	371.27	0.0016	H-1→LUMO (73%), H-1→L+1 (10%)
**S_3**	363.71	0.1487	H-2→LUMO (51%), H-2→L+1 (10%), H-1→L+1 (11%), HOMO→LUMO (15%)
**S_4**	358.34	0.0234	H-2→LUMO (12%), H-2→L+1 (30%), HOMO→LUMO (16%), HOMO→L+1 (16%)
**S_9**	321.39	0.0279	H-5→LUMO (54%), H-2→L+1 (10%)
**S_{10}**	319.48	0.0003	H-3→LUMO (53%), H-3→L+1 (45%)
**S_{11}**	312.24	0.0881	H-8→LUMO (12%), H-8→L+1 (14%), H-7→L+1 (10%), H-6→LUMO (11%), H-5→L+1 (23%)
**S_{12}**	309.32	0.0158	H-4→LUMO (21%), H-4→L+1 (69%)

Table S6. Composition (%) of Frontier MOs in terms of ligands and metals in the ground state in MeOH for **7**$^-$–**9**$^-$.

$[Pt_2(bzq)_2(p\text{-}MeC_6H_4)_2(\mu\text{-}CN)]^-$					
MO	eV	Pt	bzq	CN	*p*-MeC_6H_4
LUMO+5	-0.06	9	87	0	4
LUMO+4	-0.21	15	75	6	4
LUMO+3	-1.02	5	94	0	0
LUMO+2	-1.11	7	90	3	0
LUMO+1	-1.57	3	97	0	1
LUMO	-1.61	5	93	1	1
HOMO	-5.27	39	55	5	0
HOMO-1	-5.41	29	5	1	64
HOMO-2	-5.45	23	6	2	69
HOMO-3	-5.59	19	78	2	1
HOMO-4	-5.73	87	3	0	9
HOMO-5	-5.83	86	4	0	10

$[Pt_2(ppy)_2(p\text{-}MeC_6H_4)_2(\mu\text{-}CN)]^-$

MO	eV	Pt	ppy	CN	*p*-MeC_6H_4
LUMO+5	0.15	31	53	1	15
LUMO+4	-0.11	30	54	12	3
LUMO+3	-0.77	1	98	0	0
LUMO+2	-0.78	1	98	0	0
LUMO+1	-1.39	6	93	0	1
LUMO	-1.48	9	88	2	1
HOMO	-5.33	47	46	6	0
HOMO-1	-5.42	29	4	1	66
HOMO-2	-5.45	23	5	2	70
HOMO-3	-5.71	59	35	1	5
HOMO-4	-5.72	56	38	1	4
HOMO-5	-5.81	85	5	0	10

$[Pt_2(dfppy)_2(p\text{-}MeC_6H_4)_2(\mu\text{-}CN)]^-$

MO	eV	Pt	dfppy	CN^-	*p*-MeC_6H_4
LUMO+5	-0.02	25	69	1	6
LUMO+4	-0.29	23	63	11	2
LUMO+3	-0.75	1	98	0	0
LUMO+2	-0.76	1	99	0	0
LUMO+1	-1.45	6	93	0	1
LUMO	-1.55	10	87	3	1
HOMO	-5.48	31	8	2	59
HOMO-1	-5.52	27	9	2	63
HOMO-2	-5.56	41	36	5	18
HOMO-3	-5.82	87	3	0	10
HOMO-4	-5.91	75	16	0	9
HOMO-5	-5.93	34	61	2	3

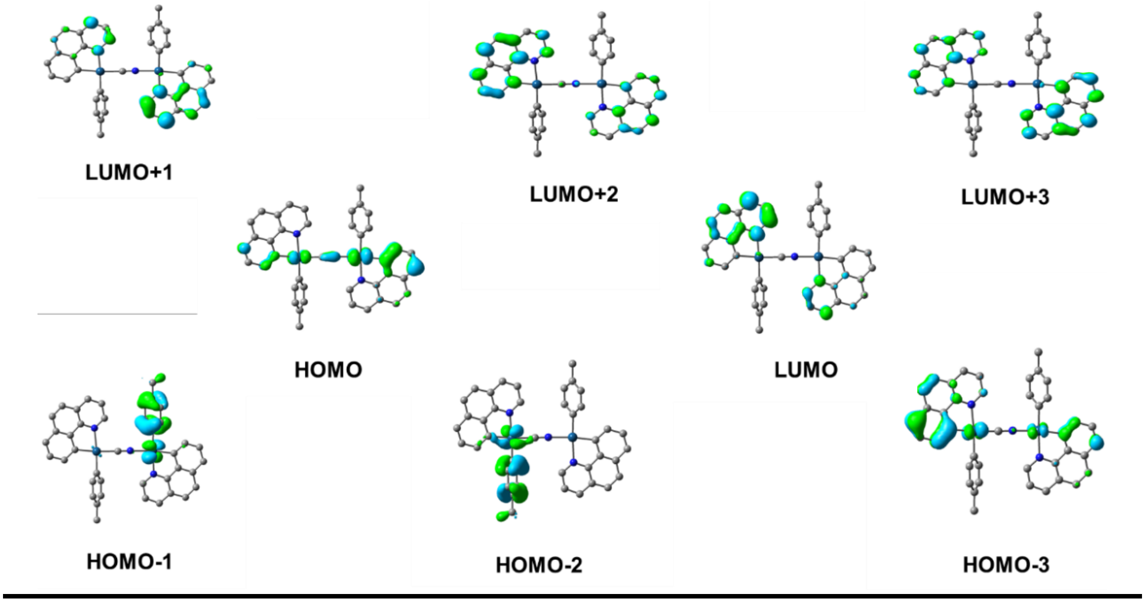

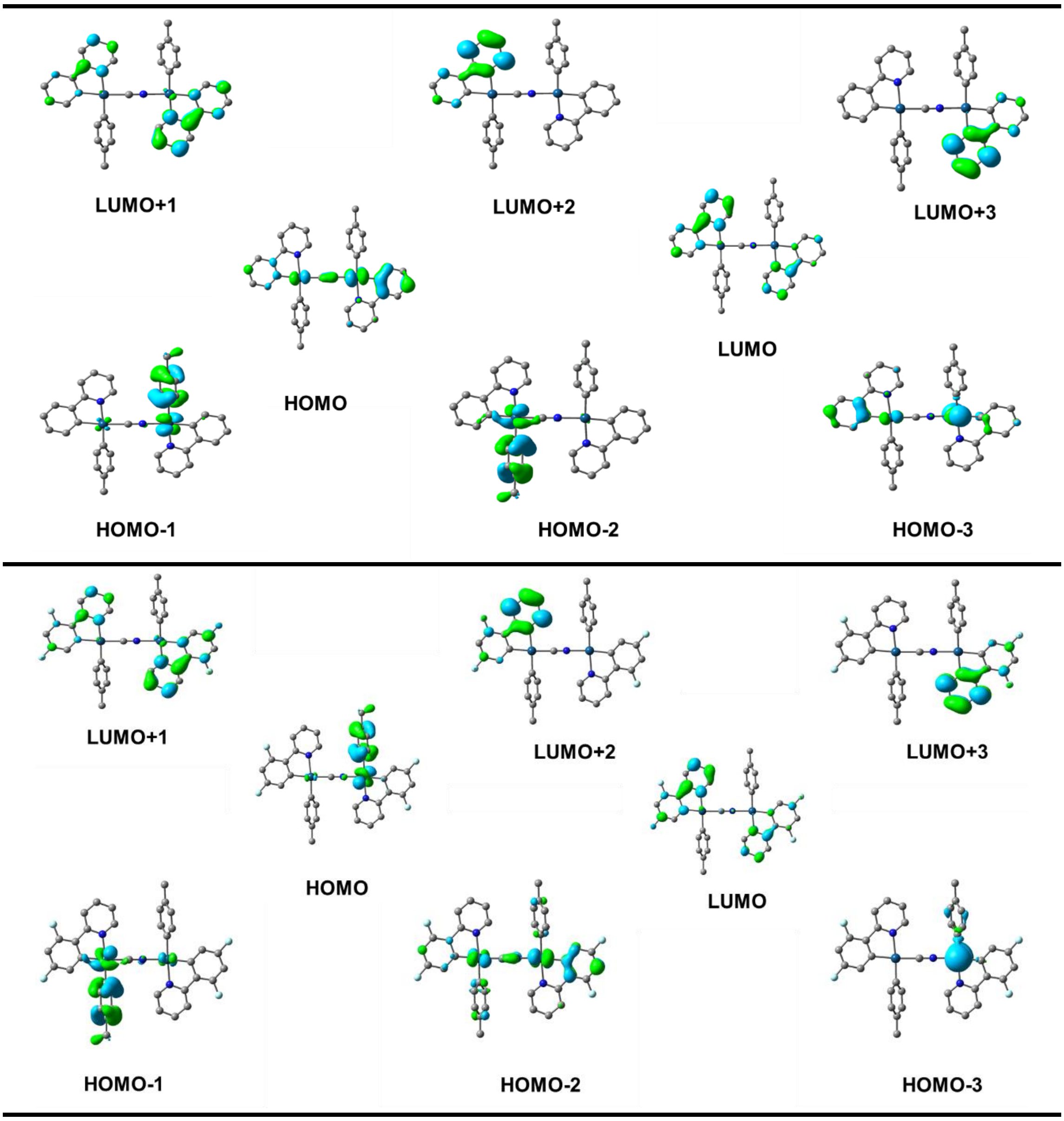

Figure S17. Selected frontier molecular orbitals for (a) $\mathbf{7}^-$, (b) $\mathbf{8}^-$ and (c) $\mathbf{9}^-$ in the ground state in solution (MeOH).

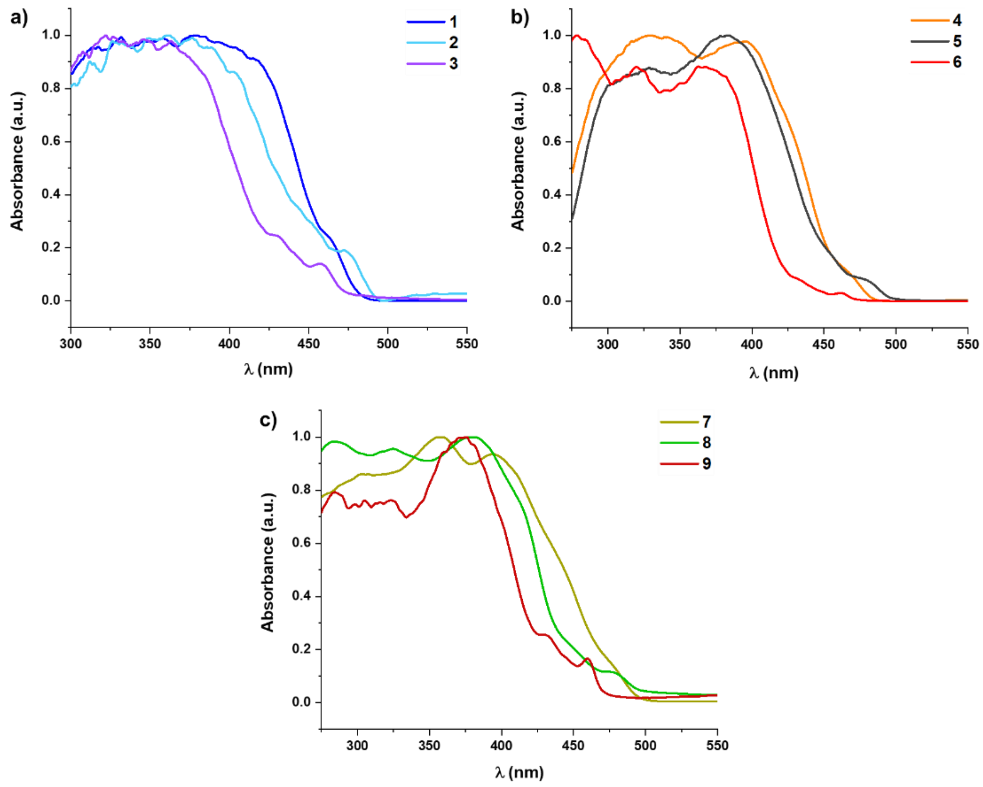

Figure S18. Absorption spectra in solid state of complexes (a) **1**–**3**, (b) **4**–**6** and (c) **7**–**9**.

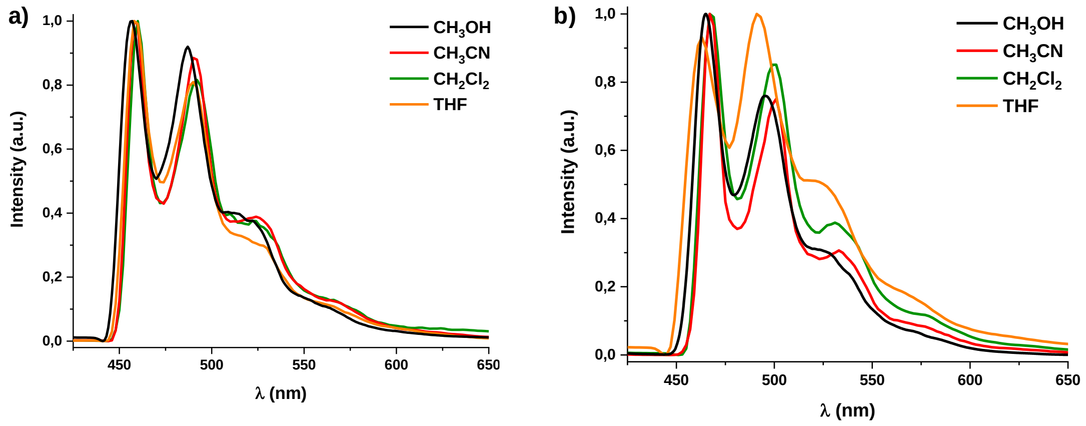

Figure S19. Emission spectra of (a) **6** and (b) **9** in different solvents (5 x 10^{-5} M) at 77 K.

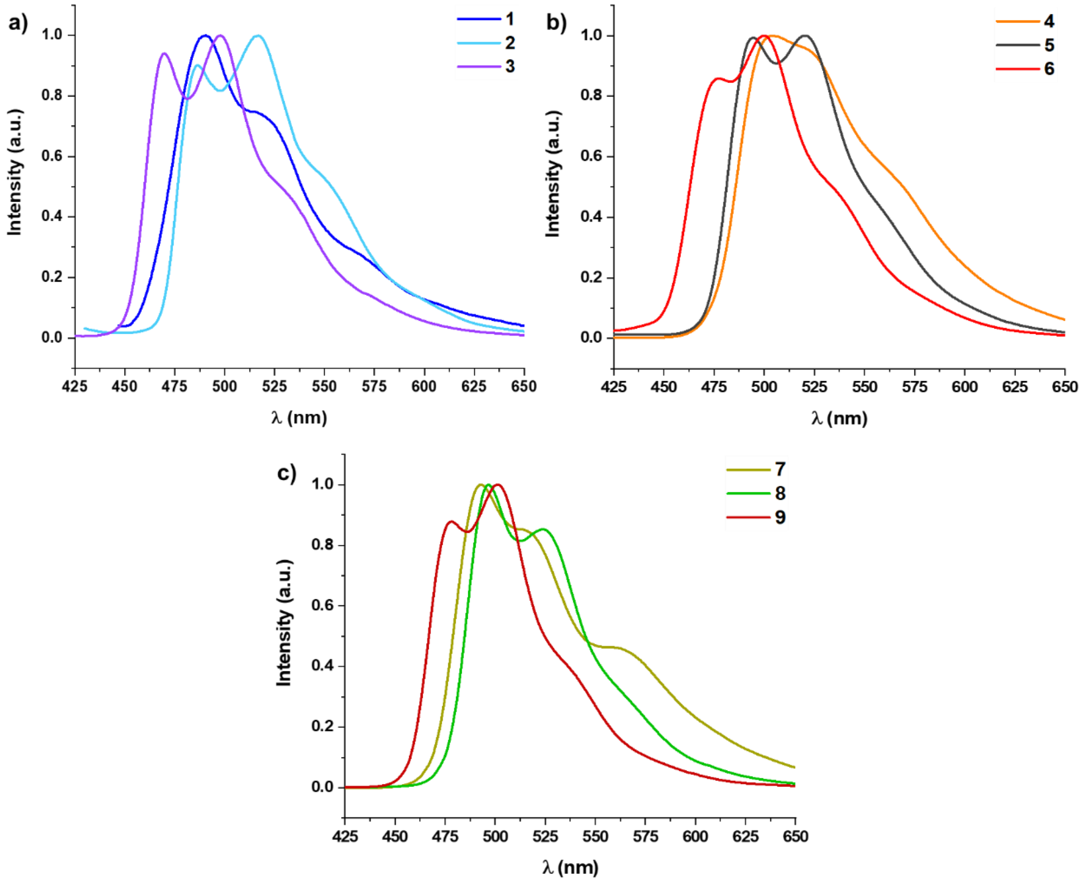

Figure S20. Emission spectra of complexes (a) **1**–**3**, (b) **4**–**6** and (c) **7**–**9** in PS film (1 % *wt*).

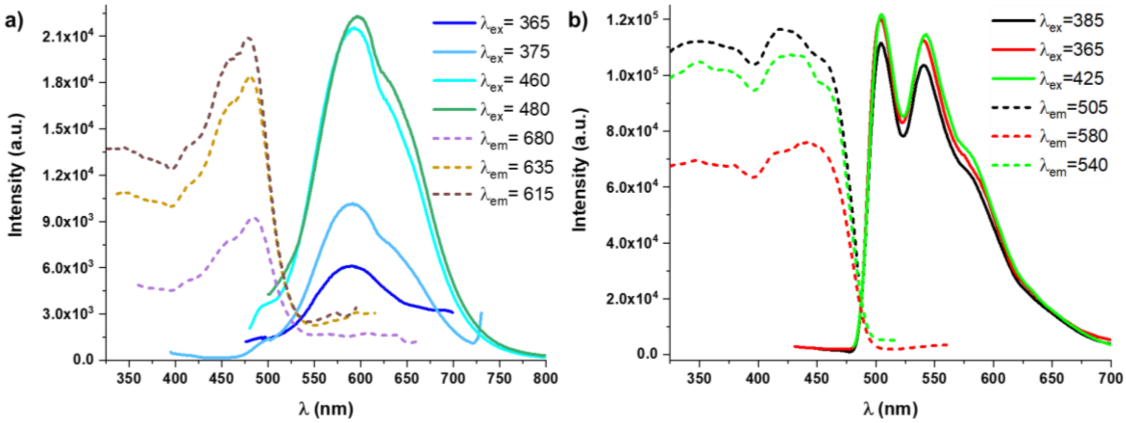

Figure S21. Excitation (- - -) and emission (—) of **1** in solid state at (a) 298 K, (b) 77 K

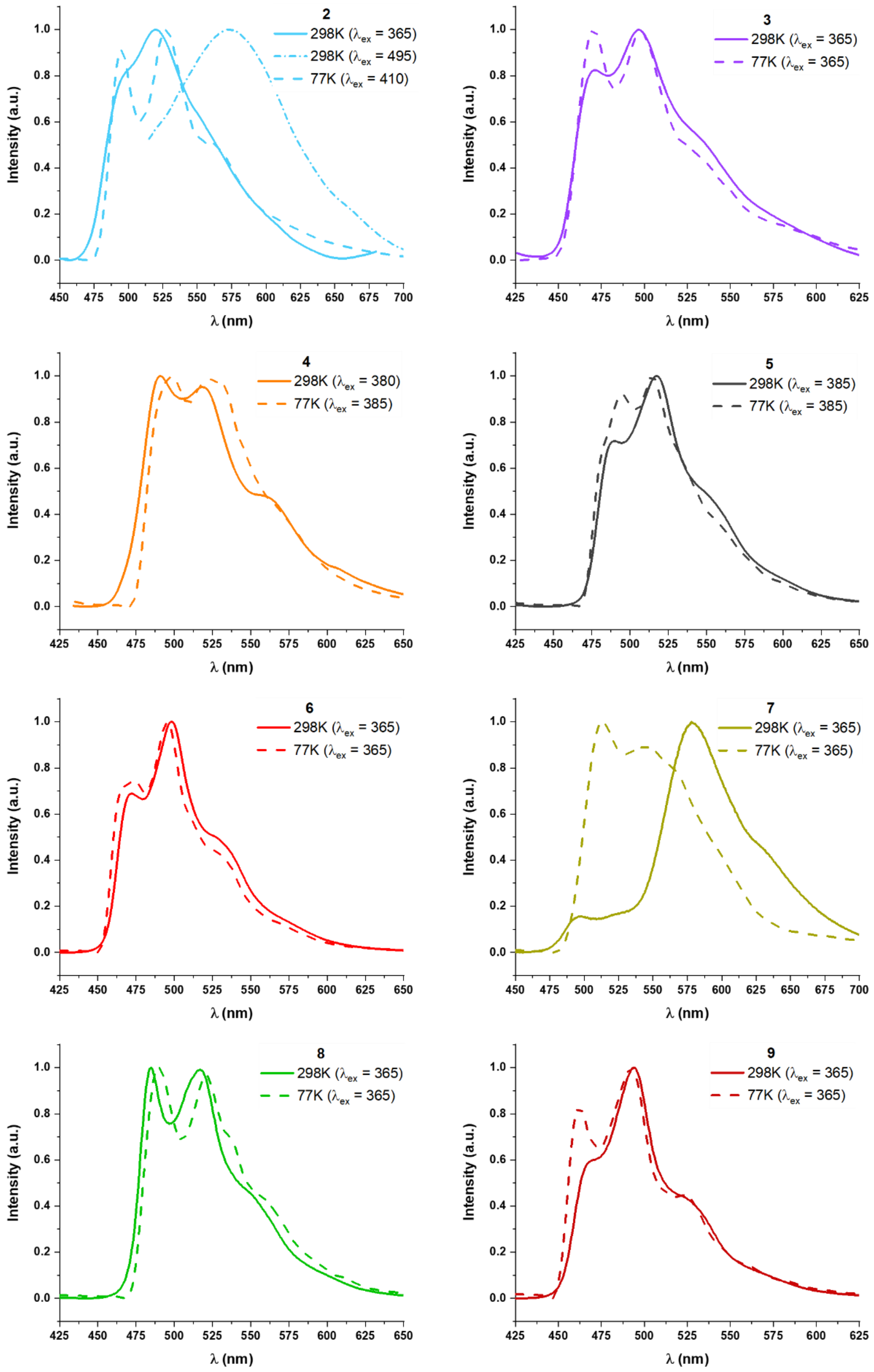

Figure S22. Emission spectra of complexes **2**–**9** in solid state at 298 and 77 K.

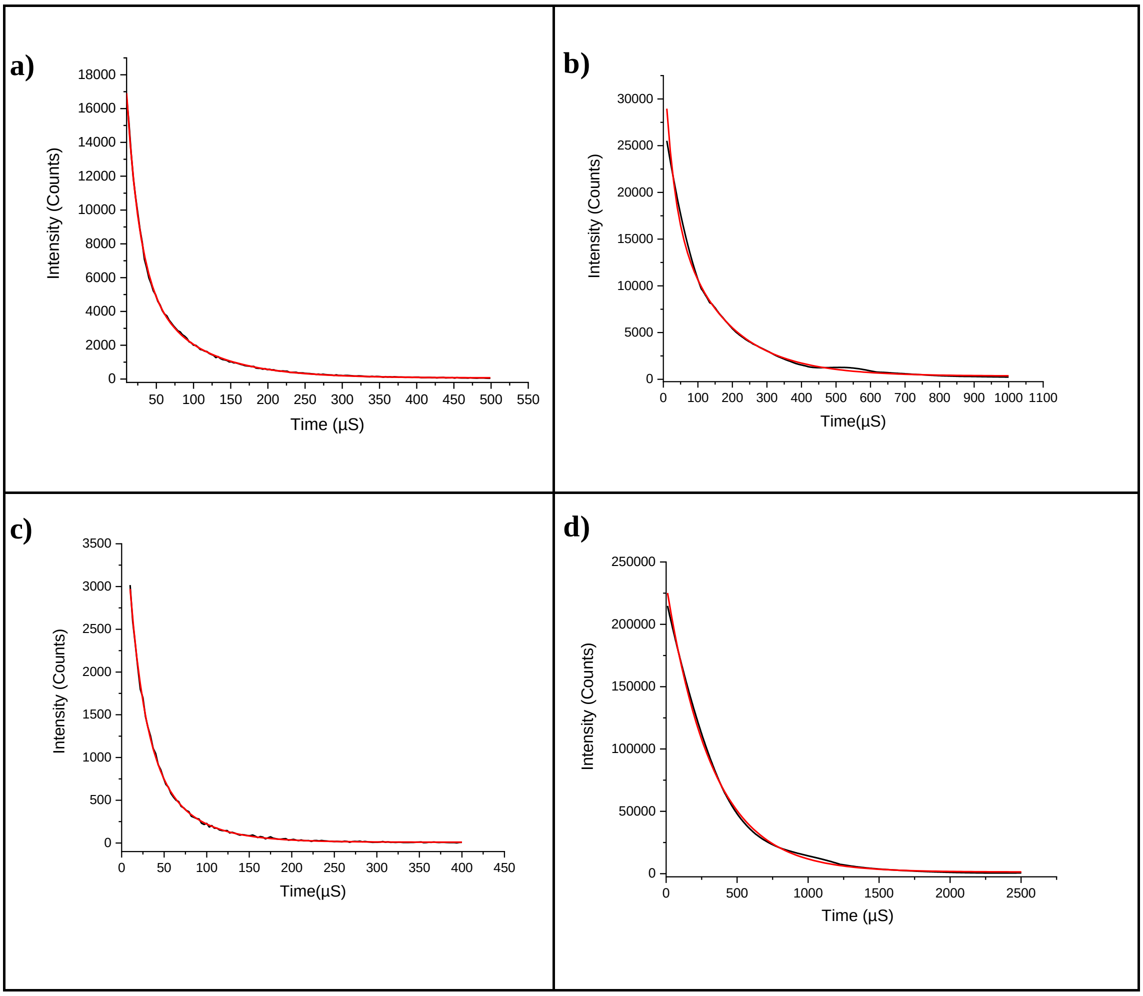

Figure S23. Excited state lifetime decays for **1** in solid-state (**a**) at 298 K, (**b**) at 77 K, (**c**) PS, (**d**) MeOH 5 × 10^{-5} M at 77 K. The red line represents the one/bi-exponential decay fitting.

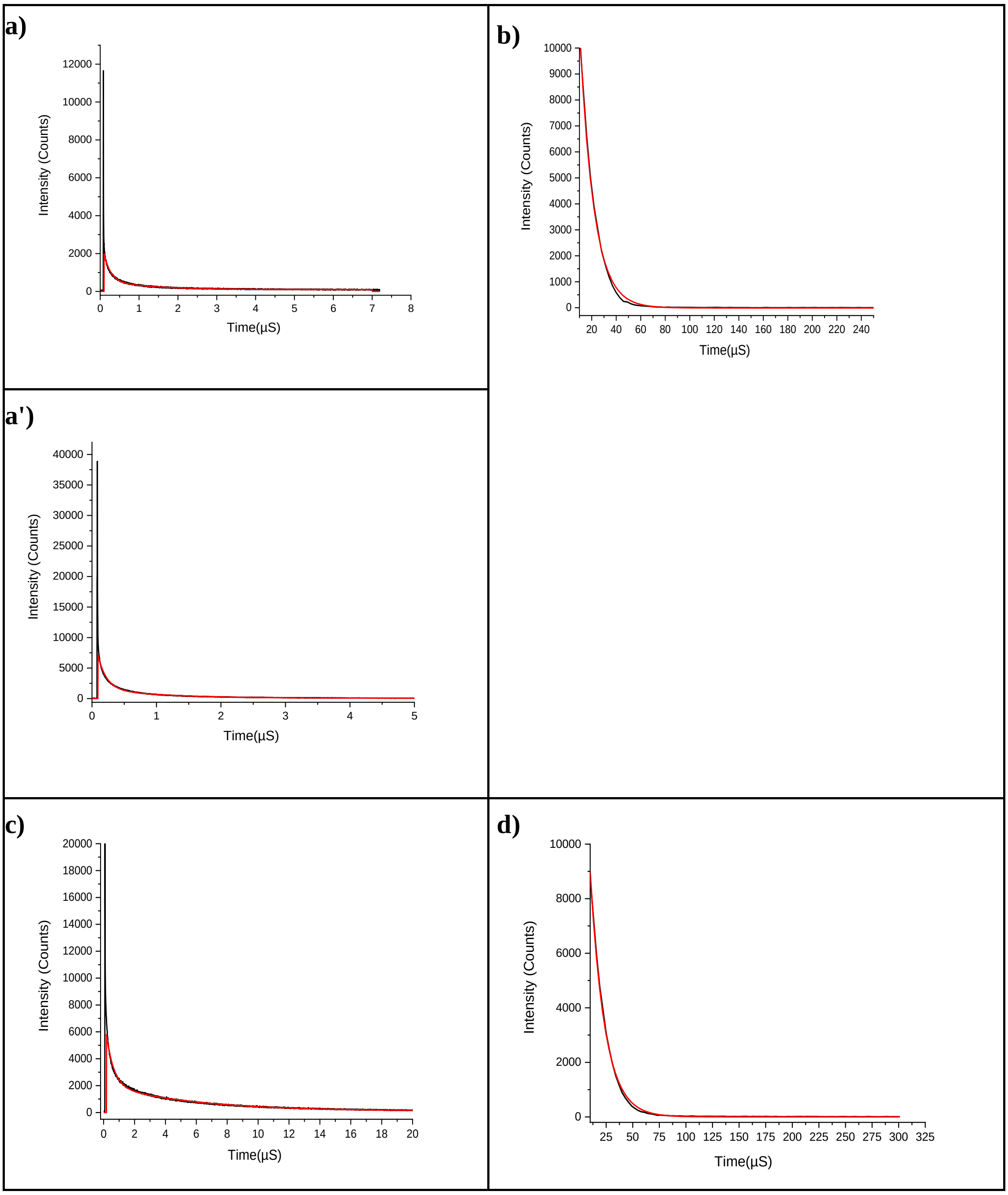

Figure S24. Excited state lifetime decays for **2** in solid-state at 298 K (**a**) λ_{em} = 500nm, (**a'**) λ_{em} = 575nm, (**b**) at 77 K, (**c**) PS, (**d**) MeOH 5×10^{-5} M at 77 K. The red line represents the one/bi-exponential decay fitting.

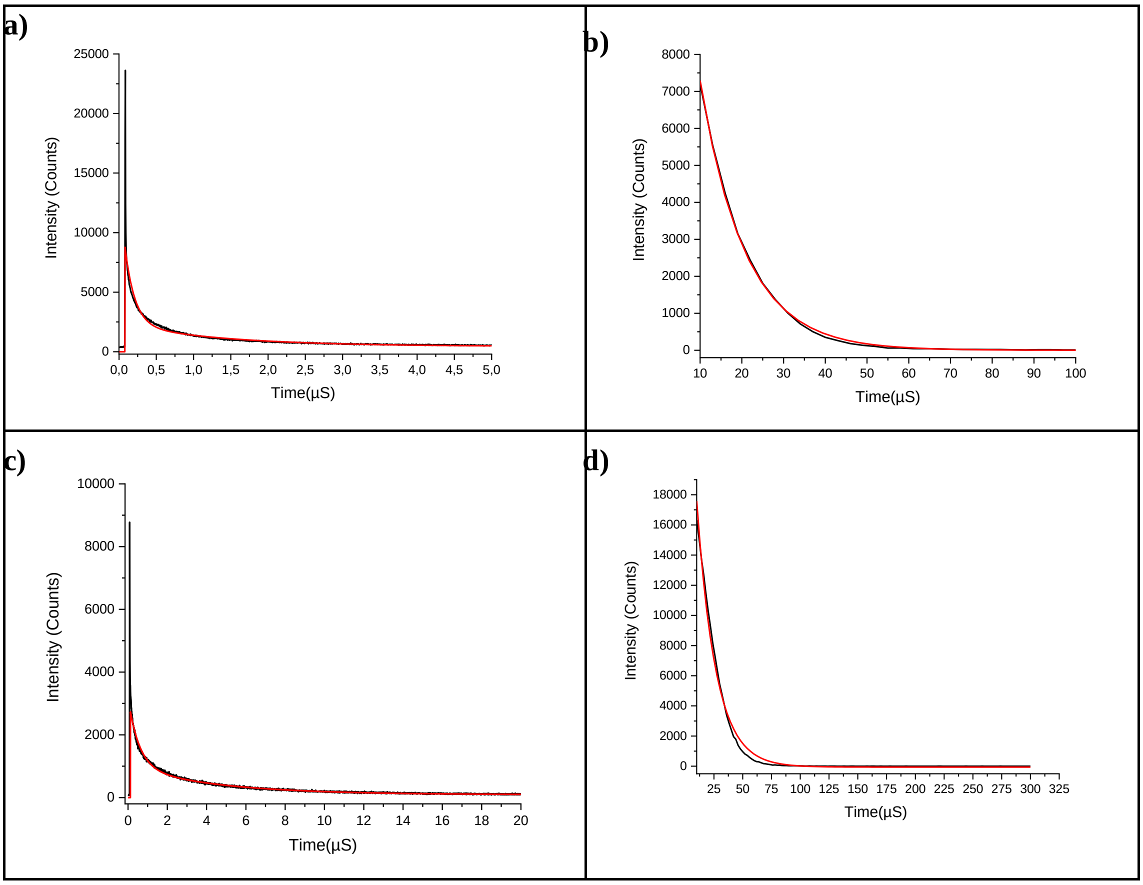

Figure S25. Excited state lifetime decays for **3** in solid-state (**a**) at 298 K, (**b**) at 77 K, (**c**) PS, (**d**) MeOH 5×10^{-5} M at 77 K. The red line represents the one/bi-exponential decay fitting.

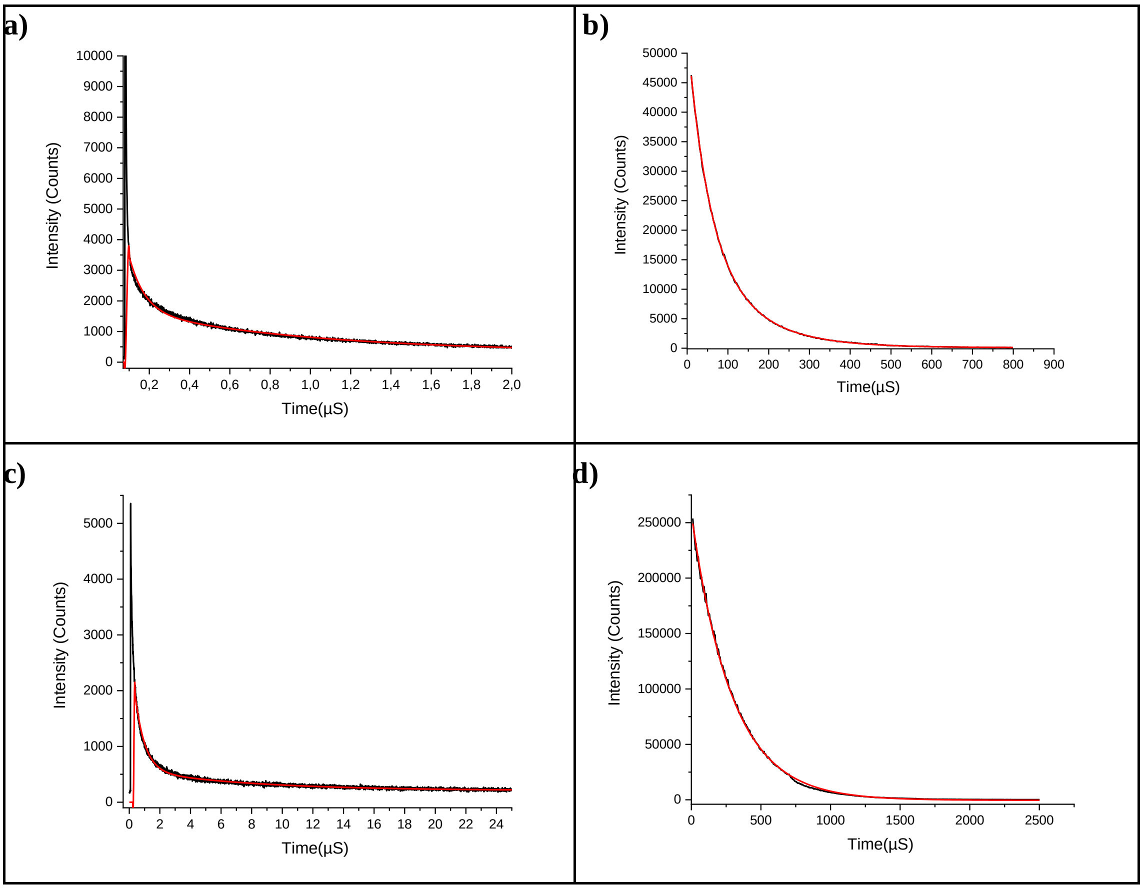

Figure S26. Excited state lifetime decays for **4** in solid-state (**a**) at 298 K, (**b**) at 77 K, (**c**) PS, (**d**) MeOH 5 × 10^{-5} M at 77 K. The red line represents the one/bi-exponential decay fitting.

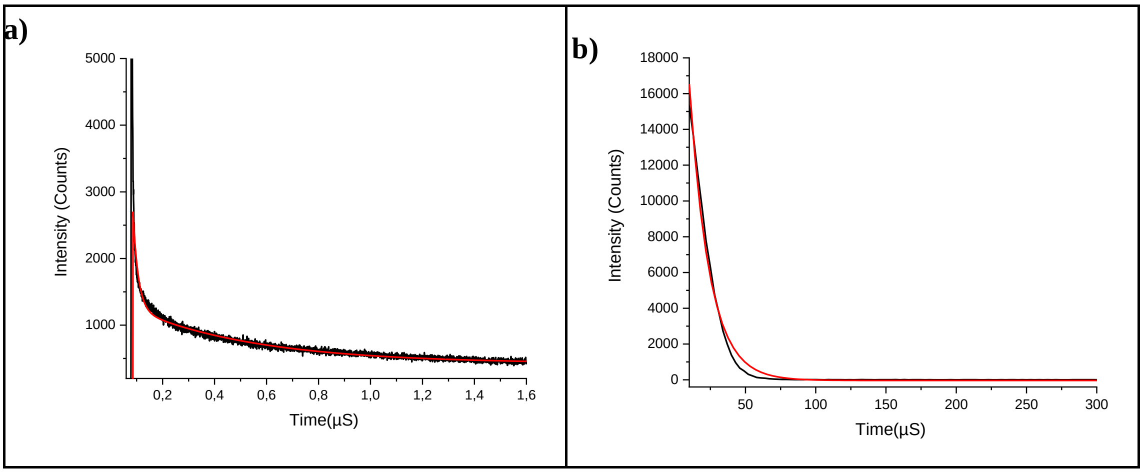

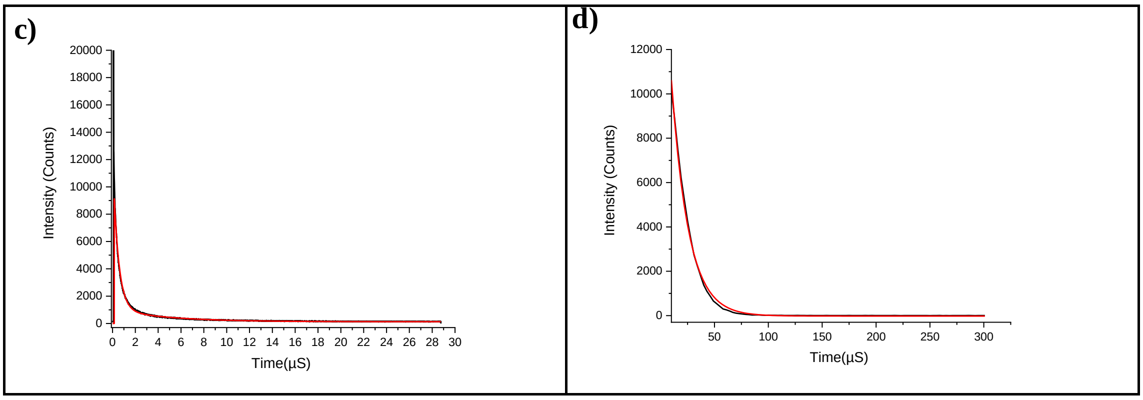

Figure S27. Excited state lifetime decays for **5** in solid-state (**a**) at 298 K, (**b**) at 77K, (**c**) PS, (**d**) MeOH 5 × 10^{-5} M at 77 K. The red line represents the one/bi-exponential decay fitting.

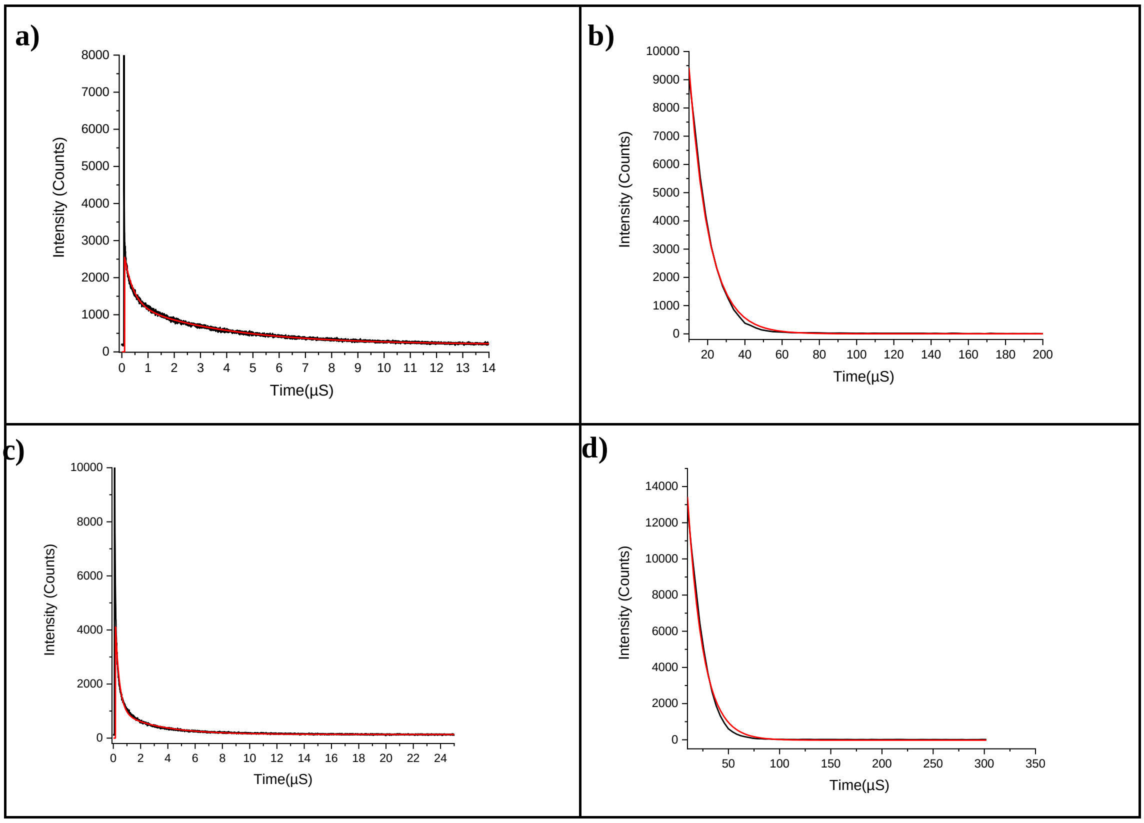

Figure S28. Excited state lifetime decays for **6** in solid-state (**a**) at 298K, (**b**) at 77K, (**c**) PS, (**d**) MeOH 5 × 10^{-5} M at 77 K. The red line represents the one/bi-exponential decay fitting.

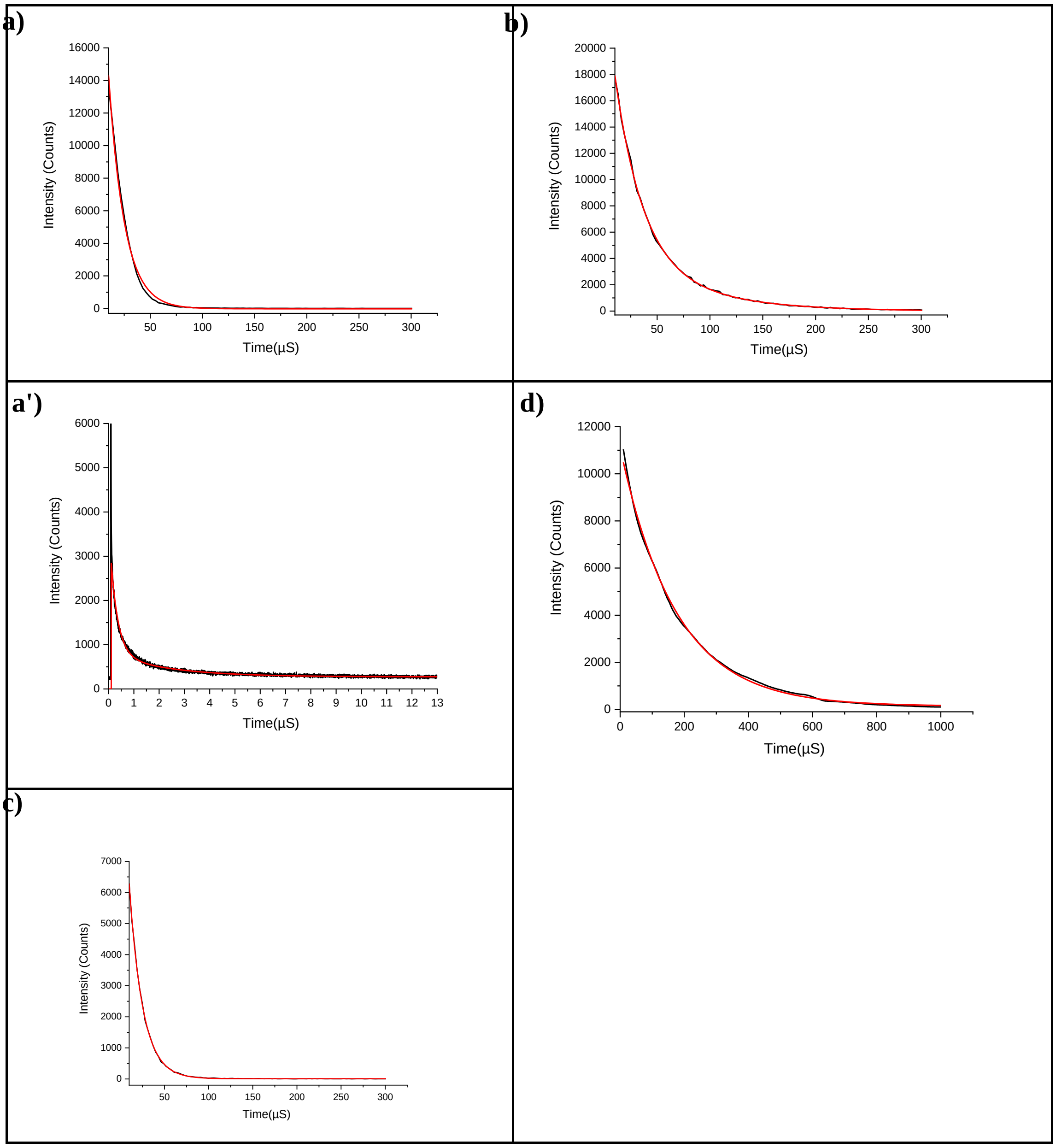

Figure S29. Excited state lifetime decays for **7** in solid-state at 298 K (**a**) λ_{em} = 500 nm, (**a'**) λ_{em} = 575 nm, (**b**) at 77K, (**c**) PS, (**d**) MeOH 5 × 10^{-5} M at 77 K. The red line represents the one/bi-exponential decay fitting.

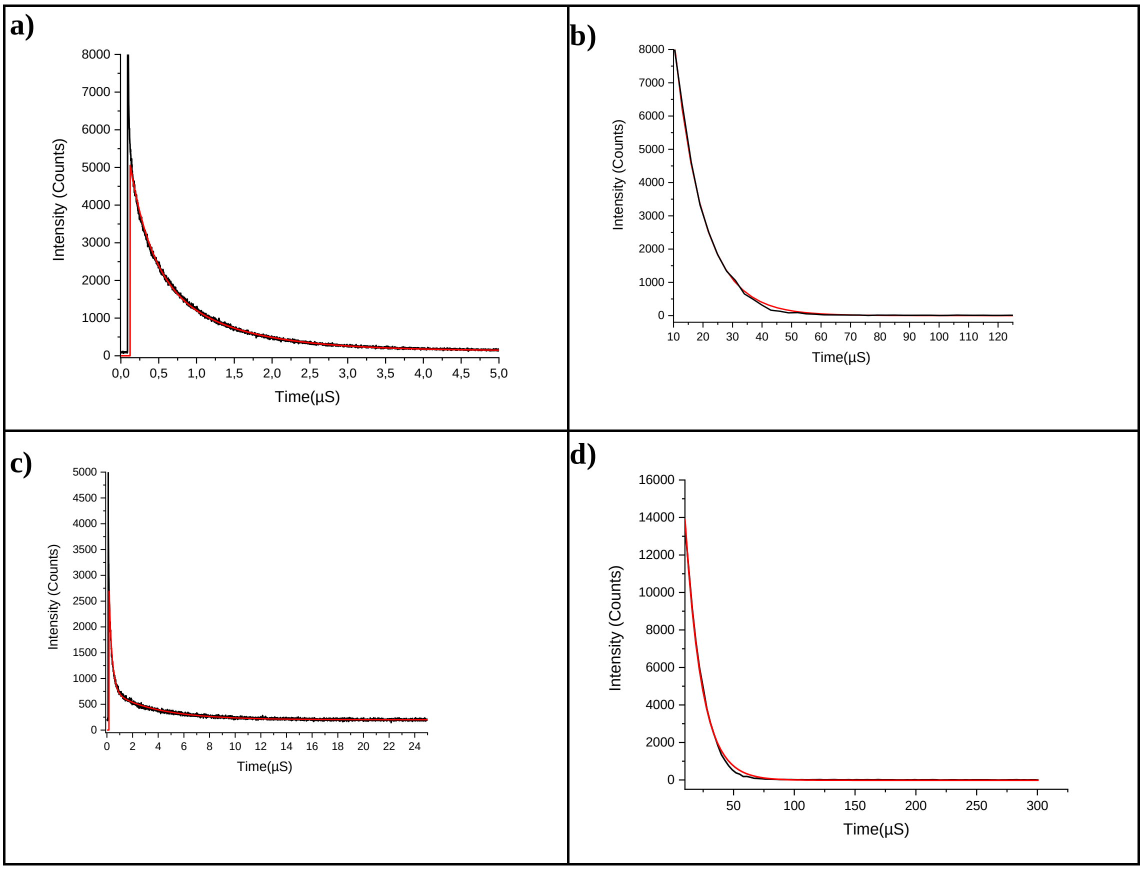

Figure S30. Excited state lifetime decays for **8** in solid-state (**a**) at 298K, (**b**) at 77K, (**c**) PS, (**d**) MeOH 5 × 10^{-5} M at 77 K. The red line represents the one/bi-exponential decay fitting.

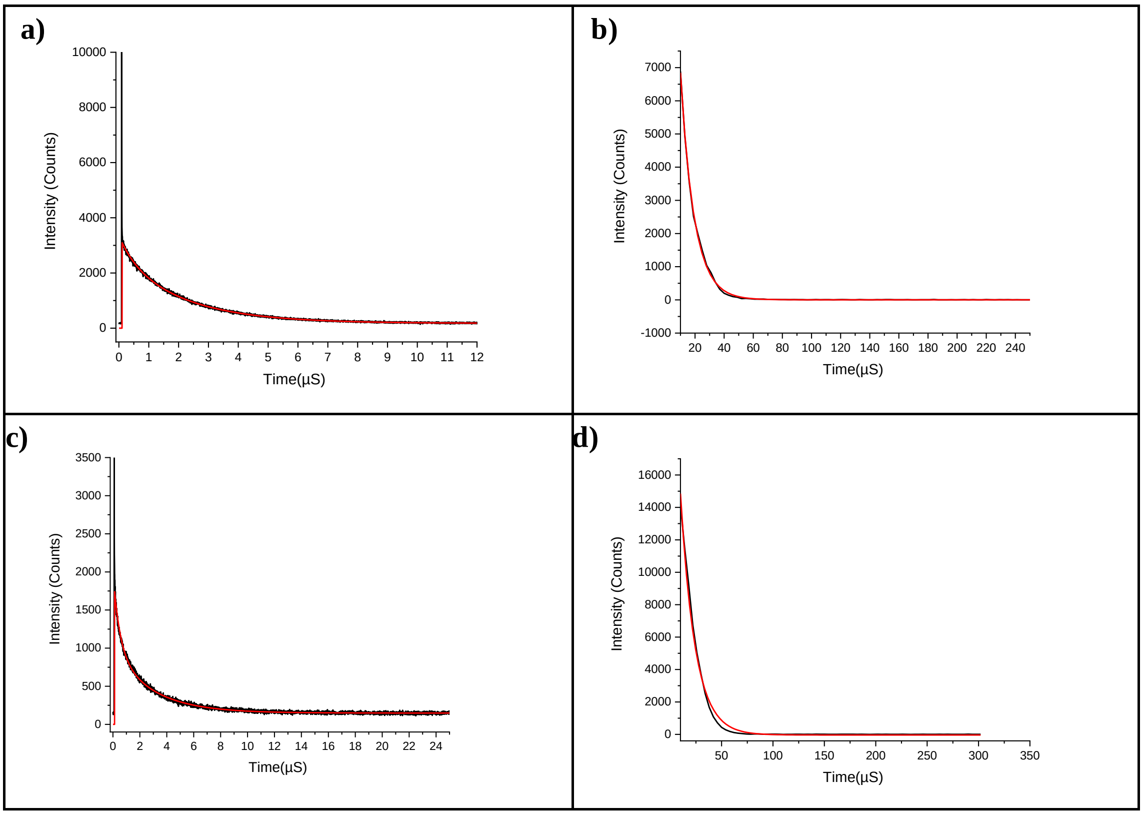

Figure S31. Excited state lifetime decays for **9** in solid-state (**a**) at 298 K, (**b**) at 77 K, (**c**) PS, (**d**) MeOH 5 × 10^{-5} M 77 K. The red line represents the one/bi-exponential decay fitting.

Table S7. Plots and composition (%) of frontier MOs of the first triplet state in MeOH for $\mathbf{4^{-}} - \mathbf{6^{-}}$

**[Pt(bzq)(*p*-MeC$_6$H$_4$)(CN)]$^{-}$**	
**SOMO**	**SOMO-1**
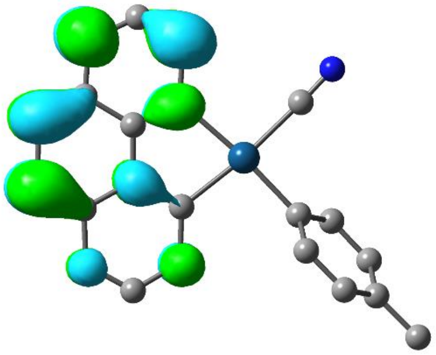	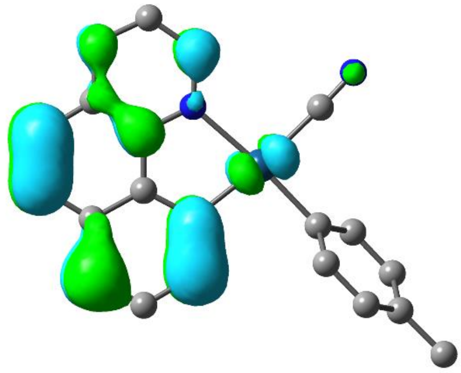
-3.06 eV<br>Pt 1%, bzq 99%, CN^{-} 0 %,<br>*p*-MeC$_6$H$_4$ 0%	-4.00 eV<br>Pt 8%, bzq 90%, CN^{-} 1 %,<br>*p*-MeC$_6$H$_4$ 1%
**[Pt(ppy)(*p*-MeC$_6$H$_4$)(CN)]$^{-}$**	
**SOMO**	**SOMO-1**
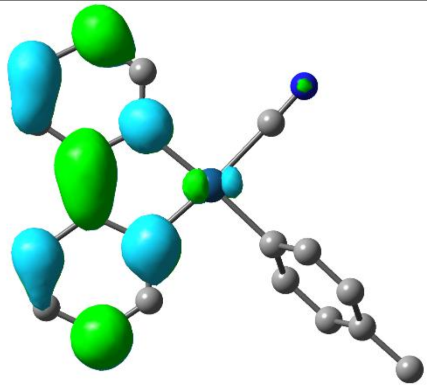	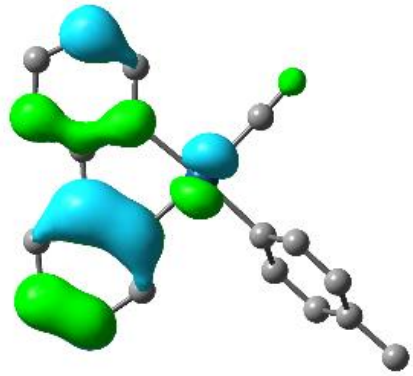
-3.01 eV<br>Pt 6%, ppy 92%, CN^{-} 1 %,<br>*p*-MeC$_6$H$_4$ 0%	-4.00 eV<br>Pt 17%, ppy 81%, CN^{-} 2 %,<br>*p*-MeC$_6$H$_4$ 0%
**[Pt(dfppy)(*p*-MeC$_6$H$_4$)(CN)]$^{-}$**	
**SOMO**	**SOMO-1**
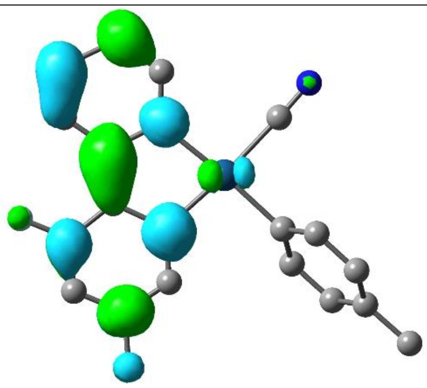	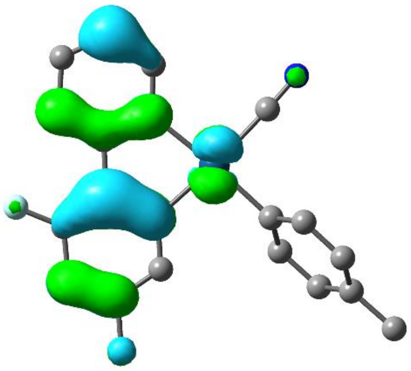
-3.11 eV<br>Pt 6%, dfppy 92%, CN^{-} 1 %,<br>*p*-MeC$_6$H$_4$ 1%	-4.18 eV<br>Pt 15%, dfppy 83%, CN^{-} 1 %,<br>*p*-MeC$_6$H$_4$ 1%

Table S8. Plots and composition (%) of frontier MOs of the first triplet state in MeOH for **7**$^-$– **9**$^-$

**$[Pt_2(bzq)_2(p\text{-}MeC_6H_4)_2(\mu\text{-}CN)]^-$**	
**SOMO**	**SOMO-1**
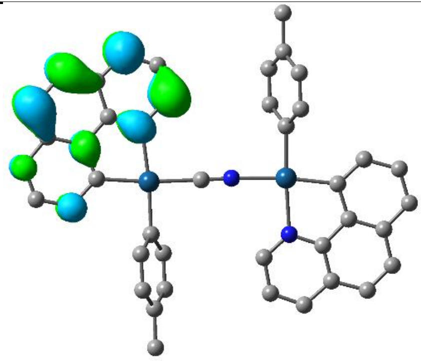	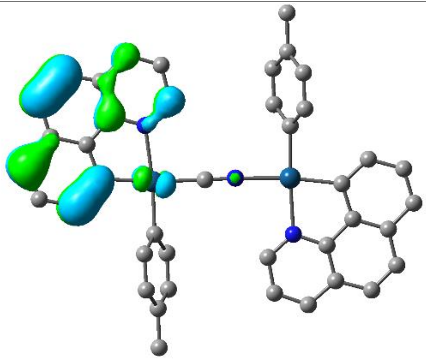
-3.13 eV<br>Pt 1%, bzq 99%, CN^- 0 %,<br>*p*-MeC_6H_4 0%	-4.06 eV<br>Pt 7%, bzq 92%, CN^- 1 %,<br>*p*-MeC_6H_4 0%
**$[Pt_2(ppy)_2(p\text{-}MeC_6H_4)_2(\mu\text{-}CN)]^-$**	
**SOMO**	**SOMO-1**
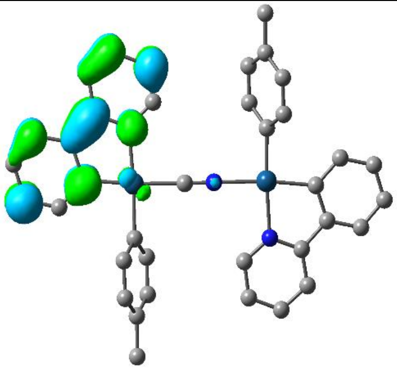	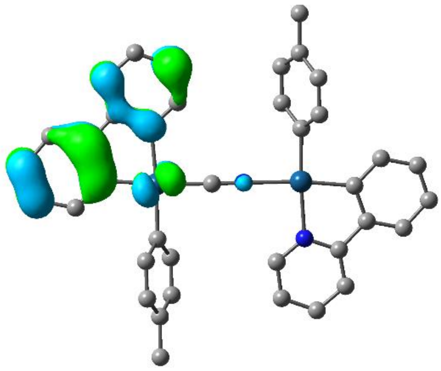
-3.09 eV<br>Pt 6%, ppy 92%, CN^- 1 %,<br>*p*-MeC_6H_4 1%	-4.07 eV<br>Pt 15%, ppy 83%, CN^- 2 %,<br>*p*-MeC_6H_4 0%
**$[Pt_2(dfppy)_2(p\text{-}MeC_6H_4)_2(\mu\text{-}CN)]^-$**	
**SOMO**	**SOMO-1**
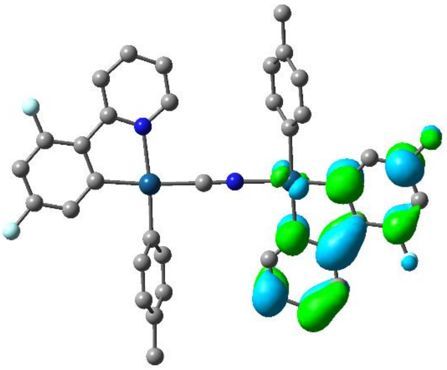	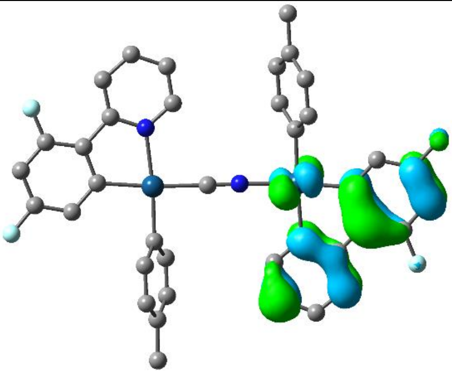
-3.11 eV<br>Pt 7%, dfppy 92%, CN^- 1 %,<br>*p*-MeC_6H_4 1%	-4.19 eV<br>Pt 18%, dfppy 80%, CN^- 1 %,<br>*p*-MeC_6H_4 1%

Table S9. Calculated energies of emission (λ_{em} (nm)) for anions **4**$^-$ – **9**$^-$ in solution of MeOH.

**Complex**	**λ_{em} (nm)**
$[Pt(bzq)(p\text{-}MeC_6H_4)(CN)]^-$ (**4**$^-$, **1**$^-$)	553
$[Pt(ppy)(p\text{-}MeC_6H_4)(CN)]^-$ (**5**$^-$, **2**$^-$)	537
$[Pt(dfppy)(p\text{-}MeC_6H_4)(CN)]^-$ (**6**$^-$, **3**$^-$)	515
$[Pt_2(bzq)_2(p\text{-}MeC_6H_4)_2(\mu\text{-}CN)]^-$ (**7**$^-$)	550
$[Pt_2(ppy)_2(p\text{-}MeC_6H_4)_2(\mu\text{-}CN)]^-$ (**8**$^-$)	536
$[Pt_2(dfppy)_2(p\text{-}MeC_6H_4)_2(\mu\text{-}CN)]^-$ (**9**$^-$)	517
